# scGET: Predicting Cell Fate Transition During Early Embryonic Development by Single-cell Graph Entropy

**DOI:** 10.1016/j.gpb.2020.11.008

**Published:** 2021-12-24

**Authors:** Jiayuan Zhong, Chongyin Han, Xuhang Zhang, Pei Chen, Rui Liu

**Affiliations:** 1 School of Mathematics, South China University of Technology, Guangzhou 510640, China; 2 School of Biology and Biological Engineering, South China University of Technology, Guangzhou 510640, China; 3 School of Computer Science and Engineering, South China University of Technology, Guangzhou 510640, China; 4 Pazhou Lab, Guangzhou 510330, China

**Keywords:** Single-cell graph entropy, Critical transition, Embryonic differentiation, Dark gene, Cell fate commitment

## Abstract

During early embryonic development, **cell fate commitment** represents a **critical transition** or “tipping point” of **embryonic differentiation**, at which there is a drastic and qualitative shift of the cell populations. In this study, we presented a computational approach, scGET, to explore the gene–gene associations based on single-cell RNA sequencing (scRNA-seq) data for critical transition prediction. Specifically, by transforming the gene expression data to the local network entropy, the **single-cell graph entropy** (SGE) value quantitatively characterizes the stability and criticality of gene regulatory networks among cell populations and thus can be employed to detect the critical signal of cell fate or lineage commitment at the single-cell level. Being applied to five scRNA-seq datasets of embryonic differentiation, scGET accurately predicts all the impending cell fate transitions. After identifying the “**dark genes**” that are non-differentially expressed genes but sensitive to the SGE value, the underlying signaling mechanisms were revealed, suggesting that the synergy of dark genes and their downstream targets may play a key role in various cell development processes. The application in all five datasets demonstrates the effectiveness of scGET in analyzing scRNA-seq data from a network perspective and its potential to track the dynamics of cell differentiation. The source code of scGET is accessible at https://github.com/zhongjiayuna/scGET_Project.

## Introduction

Complex systems may switch abruptly to a contrasting state through a critical transition [1]. In recent years, detecting critical transitions for general systems, such as ecosystem systems [2], [3], climate systems [4], financial systems [5], [6], and epidemic models [7], has drawn more and more attention. In biomedical fields, the rapid growth of single-cell datasets has shed new light on the complex mechanisms underlying cellular heterogeneity. In these single-cell experiments, the cell fate commitment represents a critical state transition or “tipping point”, at which complex systems undergo a qualitative shift. Characterizing and predicting such critical transition is crucial for patient-specific disease modeling and drug testing [8]. Recent studies have provided a plethora of statistical quantities, such as variance, correlation coefficient, and coordination of gene expression, to detect a cell fate transition of embryonic differentiation [8], [9]. However, these statistical quantities mainly focused on the analyses at the gene expression level, while single-cell RNA sequencing (scRNA-seq) may offer the opportunity to gain an insight into the cell-specific network systems. In contrast to gene expression of single-cell data, a cell-specific network is a stable form against the time and condition [10] and thus reliably characterizes the biological processes such as cell fate commitment. Such a network system is viewed as a nonlinear dynamical system with interacting variables/biomolecules, whose dynamics can be roughly divided into three stages: before-transition stage, critical stage at which cell fate commitment occurs, and after-transition stage [11], [12]. However, to characterize the dynamics of the biological system and accurately detect the tipping point or critical stage from single-cell datasets is challenging. Comparing with conventional bulk-cell information, single-cell analysis suffers from high dimensional, noisy, sparse, and heterogeneous samples.

**Figure 1:**
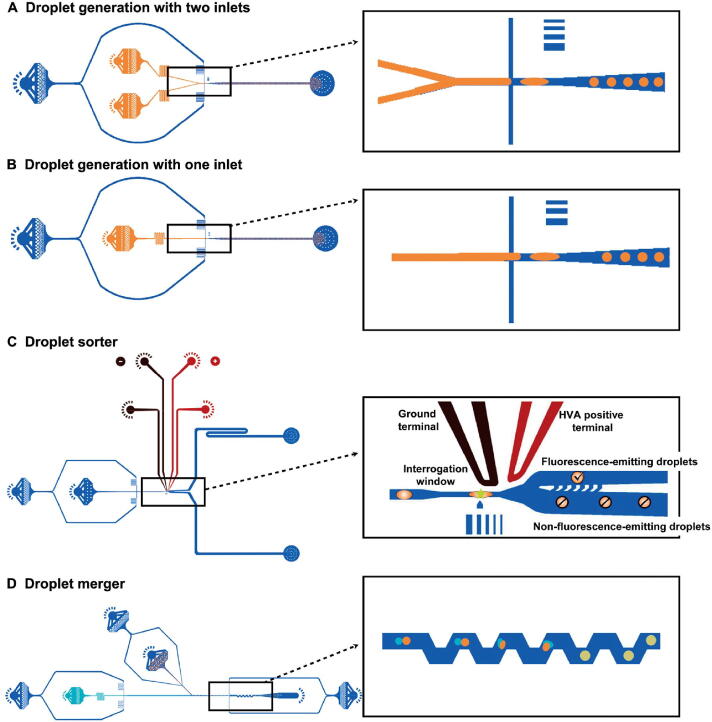
**Schematic illustration of SGE** **A.** Drawing scatter diagrams for every two genes. In the diagram, each point represents a cell, whose location is determined by the expression levels of the two genes that are mapped to the horizontal and vertical axis separately. For each gene pair $\left\{g_{i},g_{j}\right\}$, there is an edge between $g_{i}$ and $g_{j}$ in the cell-specific network of $C_{k}$ if $r_{i,j}^{\left(k\right)}$ > 0 in Equation (1). Constants $n^{\left(k\right)}\left(E_{i}\right)$ and $n^{\left(k\right)}\left(E_{j}\right)$ represent the number of the points/cells within the vertical box and the horizontal box, respectively. **B.** Constructing the cell-specific network by weight $r_{i,j}^{\left(k\right)}$ for cell $C_{k}$. Based on each local network extracted from the cell-specific network, a local SGE value is calculated from Equation (2). **C.** Illustrating signal of a critical transition by the significant increase of SGE. SGE remains low when the system is in a before-transition stage, while it increases abruptly when the system approaches to the critical stage. **D.** Identifying the dark genes. Different from traditional biomarkers based on DEGs, scGET identifies dark genes that are sensitive to the SGE value, but non-differential at the gene expression level. SGE, single-cell graph entropy; DEG, differentially expressed gene.

During the past years, many tasks of critical importance in the bioinformatics field had been addressed based on entropies, which integrate the gene expression profiles of samples with a protein–protein interaction (PPI) network. For example, network entropy provides a proxy to the elevation of the differentiation potential of a cell and has revealed key genes and signaling pathways implicated in the cancer metastasis phenotype [13], [14]. Besides, the previous studies have also explored drug sensitivity profiles in cancer cell lines, identified normal and cancer stem cell phenotypes, and quantified the differentiation potential of single cells by computing the entropy of transcriptome of a cell in the context of PPI network [15], [16], [17]. Such integration of gene expression profiles with the biomolecular network is the need of the hour, which better characterizes the dynamic changes of a biological system. Inspired by these pioneer works, we developed a computational approach from the cell-specific network viewpoint, scGET, to detect the signal of a critical transition or cell fate commitment during the embryonic differentiation process and identify non-differentially expressed genes (non-DEGs) that may play important roles in embryonic development. The utilization of single-cell graph entropy (SGE) is based on rewiring the cell-specific networks with statistical dependency, calculating a network entropy score for each localized network, as well as combining and analyzing the dynamical change of the local indices (Figure 1). Such a method can be viewed as data transformation from the “unstable” gene expression of single cells to the relatively “stable” SGE value of gene associations (Figure 1A and B). This SGE value can be analyzed by any traditional scRNA-seq algorithm for dimension reduction and cell clustering analysis by simply replacing the original gene expression levels with the local SGE values. Notably, scGET has capabilities beyond traditional expression-based methods, that is, scGET aims at exploring the dynamically differential information at a single-cell level, and thus identifying a critical stage during the progression of a biological system (Figure 1C). Specifically, we detect the signature of an imminent critical transition by a significant increase of the SGE value, which indeed reflects the dynamic change of cell heterogeneity and coordination of gene expression. scGET has been applied to five scRNA-seq embryonic differentiation datasets downloaded from the NCBI Gene Expression Omnibus (GEO) database. For these embryonic time-course differentiation datasets, the predicted cell fate transitions agree with the observation in original experiments. In these applications, it is also demonstrated, from the dynamic perspective, that scGET has a good performance in temporal clustering of cells, that is, the clustering analysis based on the SGE value accurately distinguishes the cell heterogeneity over time at single-cell resolution. Besides, in the analysis of these single-cell datasets, the proposed method identifies a few “dark genes”, which are non-differential in gene expression but sensitive to the SGE value and may play important roles in embryonic development (Figure 1D). The functional analysis suggests that the synergy of dark genes and their downstream targets may play a key role in various cell development processes. It is found that the cumulative expression of some dark genes such as growth factor *IGF1* and extracellular matrix (ECM) *LAMC2* and *COL4A1* may act as a temporal factor that triggers cell fate decisions and regulate a series of downstream molecules essential for cell proliferation and differentiation. Therefore, scGET provides a new way to analyze the scRNA-seq data and helps to track the dynamics of biological systems from the perspectives of network entropy. The successful application of scGET validated its effectiveness in the single-cell analysis.

## Method

### Theoretical basis

A cell fate transition (cell fate commitment) occurs during the dynamical process of the early embryonic differentiation [18], [19]. Generally, the dynamical process of early embryonic development can be regarded as the evolution of a nonlinear dynamical system, while the cell fate transition is viewed as a drastic or qualitative state shift at a bifurcation point [8]. Similar to disease progression [11], [20], this dynamical process is modeled as three states or stages (Figure 1C): 1) a before-transition stage with high resilience; 2) a critical stage, which is the tipping point or cell fate transition with low resilience; and 3) an after-transition stage, which is another stable state with high resilience.

In this study, the cell-specific networks are constructed based on a recently proposed statistical model [10], which provides a statistical dependency index (defined as in Equation (1)) to determine the gene associations at a single-cell level. The statistic index ranges between − 1 and 1. The positive statistical dependency value infers the statistically interacting relation between two genes, *i.e.*, there is an edge between such two genes in the cell-specific network.

### Algorithm to detect the signal of critical transition based on scGET

Given the time series of scRNA-seq data, the following algorithm is carried out to predict the critical transition.

The first step (Step 1) is to normalize the scRNA-seq data. At each time point, the logarithm log(1+x) is applied to normalize the initial gene expression matrix with M rows/genes and N columns/cells.

The second step (Step 2) is to calculate a statistical dependency index $r_{i,j}^{\left(k\right)}$. 1) We plot scatter diagrams for every two genes in a Cartesian coordinate system where the vertical and horizontal axes indicate the expression values of the two genes. For example, there are N plots in the scatter diagram for a gene pair $\left(g_{i},g_{j}\right)$ corresponding to the N cells. Each plot represents a cell whose horizontal coordinate is $E_{i}^{\left(k\right)}$ (the gene expression of $g_{i}$ in cell $C_{k}$) and the vertical coordinate is $E_{j}^{\left(k\right)}$ (the gene expression of $g_{j}$ in cell $C_{k}$) (Figure 1A). Then totally M(M-1)/2 scatter diagrams are obtained by making a scatter diagram for every two genes. 2) In the scatter diagram of genes $g_{i}$ and $g_{j}$, for cell $C_{k}$, we set two boxes near gene expression values $E_{i}^{\left(k\right)}$ and $E_{j}^{\left(k\right)}$ based on two predetermined parameters $n^{\left(k\right)}\left(E_{i}\right)=0.1N$ and $n^{\left(k\right)}\left(E_{j}\right)=0.1N$, where $n^{\left(k\right)}\left(E_{i}\right)$ and $n^{\left(k\right)}\left(E_{j}\right)$ represent the number of the points/cells in the green box of $E_{i}^{\left(k\right)}$ and the blue box of $E_{j}^{\left(k\right)}$, respectively (Figure 1A). 3) The third box is marked in red, which is the overlap of the two aforementioned boxes. Let $n^{\left(k\right)}\left(E_{i}{,E}_{j}\right)$ denote the number of points/cells in this overlapping box. 4) The statistical dependency index $r_{i,j}^{\left(k\right)}$ is designed based on the three statistics ($n^{\left(k\right)}\left(E_{i}\right)$, $n^{\left(k\right)}\left(E_{j}\right),$ and $n^{\left(k\right)}\left(E_{i}{,E}_{j}\right)$) above and defined as shown below.

(1) $$r_{i,j}^{\left(k\right)}=\frac{n^{\left(k\right)}\left(E_{i}{,E}_{j}\right)}{N}-\frac{n^{\left(k\right)}\left(E_{i}\right)}{N}\cdot \frac{n^{\left(k\right)}\left(E_{j}\right)}{N}$$

The third step (Step 3) is to construct a specific network for each cell. Based on the statistical dependency index $r_{i,j}^{\left(k\right)}$ defined in Equation (1), a cell-specific network for cell $C_{k}$ is constructed as follows. If $r_{i,j}^{\left(k\right)}$ is greater than zero, there is an edge between $g_{i}$ and $g_{j}$ in the cell $C_{k}$; otherwise, there is no edge. In this way, we construct a cell-specific network $N^{\left(k\right)}$ for cell $C_{k}$, where each edge between two genes $g_{i}$ and $g_{j}$ is decided by the dependency index $r_{i,j}^{\left(k\right)}$. The detailed flowchart and description for constructing a cell-specific network are provided in Figure S1 and Note 1 of File S1, respectively.

The fourth step (Step 4) is to extract each local network/subnetwork from the cell-specific network. Specifically, for a cell $C_{k}$, there are M local networks ${\mathrm{LN}}_{i}^{\left(k\right)}\left(i=1,2,3,\cdots ,M\right)$ corresponding to its M genes. The local network ${\mathrm{LN}}_{i}^{\left(k\right)}$ is centered at a gene $g_{i}$, whose 1st-order neighbors $\left\{g_{1}^{i},g_{2}^{i},\cdots ,g_{S}^{i}\right\}$ are the edges (Figure 1B).

The fifth step (Step 5) is to calculate the gene-specific local SGE value $H_{i}^{\left(k\right)}$ for each local network. Given a local network ${\mathrm{LN}}_{i}^{\left(k\right)}$ centered at a gene $g_{i}$, the corresponding local SGE value $H_{i}^{\left(k\right)}$ is calculated as follows.

(2) $$H_{i}^{\left(k\right)}=-\frac{1}{\log(S)}\sum_{j=1}^{S}p_{i,j}^{\left(k\right)}\log\left(p_{i,j}^{\left(k\right)}\right)$$

with

(3) $$p_{i,j}^{\left(k\right)}=\frac{r_{i,j}^{\left(k\right)}\cdot E_{j}^{\left(k\right)}}{\sum_{j=1}^{S}r_{i,j}^{\left(k\right)}\cdot E_{j}^{\left(k\right)}}$$

where $r_{i,j}^{\left(k\right)}$ represents the weight coefficient between the center gene $g_{i}$ and a neighbor $g_{j}^{i}$. The value $E_{j}^{\left(k\right)}$ represents the gene expression of a neighbor $g_{j}^{i}$ in $C_{k}$ and constant S is the number of neighbors in the local network ${\mathrm{LN}}_{i}^{\left(k\right)}$. Clearly, the local SGE value $H_{i}^{\left(k\right)}$ (Equation (2)) has been normalized to the number of nodes in a local network. After this step, the sparse gene expression matrix from the scRNA-seq data is transformed into a non-sparse graph entropy matrix (Figure 1A and B) by taking the gene association into consideration. Thus, the local SGE value $H_{i}^{\left(k\right)}$ is dependent not only on the expression of the center gene of a local network but also on the contribution from the neighboring genes.

## The sixth step (Step 6) is to calculate the cell-specific SGE value $H^{\left(k\right)}$ based on a group of genes with the highest local SGE values, *i.e.*,

(4) $$H^{\left(k\right)}=\sum_{i=1}^{T}H_{i}^{\left(k\right)},$$

where constant T is an adjustable parameter representing the number of top 5% genes with the highest local SGE values. In Equation (4), $H^{\left(k\right)}$ can be considered as the SGE value of the cell $C_{k}$ and detect the early-warning signals of the cell fate transition. At each time point *t*, the mean of the cell-specific SGE values of a certain cell population is employed as the SGE value $H_{t}$ of the dataset in the tipping-point detection, *i.e.*,

(5) $$H_{t}=\frac{1}{N}\sum_{k=1}^{M}H^{\left(k\right)}$$

where N represents the number of cells in the dataset. Figure S2 shows that different choices of T do not alter the result of identifying the tipping point.

When the system approaches to the vicinity of the critical point, the signaling genes or dynamical network biomarker (DNB) molecules exhibit obviously collective behaviors with fluctuations, leading to the property that the dependent relations of DNB members in a critical state are different from those in a before-transition state. Moreover, the local SGE value $H_{i}^{\left(k\right)}$ sharply increases when the system is near the tipping point (Figure 1C).

The seventh step (Step 7) is to identify the critical transition by the one-sample *t*-test. To quantify how well the SGE value recapitulates the critical behaviors, the one-sample *t*-test [21] is applied to determine whether there is a significant difference between the before-transition and the critical states. To determine whether the constant x is statistically different from the mean of an *n*-dimensional vector $X=(x_{1},x_{2},\cdots ,x_{n})$, the one-sample *t*-test statistic S is defined as shown below:

(6) $$S=\frac{\overset{-}{X}-x}{s/\sqrt{n}}$$

where $\overset{-}{X}$ represents the mean of vector X and constant s represents the standard deviation of the vector X. To estimate the statistical significance between $\overset{-}{X}$ and x, the P value associated with S is obtained from the *t*-distribution. There is a statistical significance between $\overset{-}{X}$ and x if P<0.05. In this study, the time point T=t is regarded as a critical point if the SGE value $H_{t}$ satisfies the following two conditions—(1) $H_{t}$ > $H_{t-1}$and (2) $H_{t}$ is significantly different $\left(P<0.05\right)$ from the prior information.

In the algorithm above, the statistical dependency index $r_{i,j}^{\left(k\right)}$ is necessary. Actually, to test how important the factor $r_{i,j}^{\left(k\right)}$ is in identifying the critical transition point, we analyzed the forms of different probabilities $p_{i,j}^{\left(k\right)}$ generated by $r_{i,j}^{\left(k\right)}\cdot E_{j}^{\left(k\right)}$ (the combination of statistical dependency index $r_{i,j}^{\left(k\right)}$ and the cell’s gene expression value $E_{j}^{\left(k\right)}$), $r_{i,j}^{\left(k\right)}$ alone, or $E_{j}^{\left(k\right)}$ alone, which is presented in Figure S3, and also illustrated in detail in Note 2 of File S1. The source code of the algorithm and related data are all publicly available at https://github.com/zhongjiayuna/scGET_Project.

### Dataset

Five unrelated real datasets have been downloaded from GEO database. These include mouse embryonic fibroblast (MEF) to neuron (MEF-to-neuron; GEO: GSE67310), neural progenitor cell (NPC) to neuron (NPC-to-neuron; GEO: GSE102066), human embryonic stem cell (hESC) to definitive endoderm cell (DEC) (hESC-to-DEC; GEO: GSE75748), mouse hepatoblast cell (MHC) to hepatocyte and cholangiocyte cell (HCC) (MHC-to-HCC; GEO: GSE90047), and mouse ESC (mESC) to mesoderm progenitor (MP) (mESC-to-MP; GEO: GSE79578).

## Results

### Detecting the signal of cell fate commitment

To demonstrate the effectiveness of scGET, the proposed method has been applied to five time-course datasets of embryonic differentiation downloaded from GEO database (http://www.ncbi.nlm.nih.gov/geo/), including MEF-to-neuron data [22], NPC-to-neuron data [23], hESC-to-DEC data [24], MHC-to-HCC data [25], and mESC-to-MP data [26]. The detailed description and sources of the datasets are provided in Note 3 of File S1. The cell-specific SGE value (Equation (4)) of each single cell was calculated according to the algorithm described in the Method section. At each time point, the mean SGE value was taken to quantitatively measure the criticality of the cell population. An SGE curve across all time points was then employed to illustrate the detection of cell fate transition. The successful detection of the cell fate transitions during embryonic cell differentiation in these five datasets validates the effectiveness and accuracy of scGET.

**Figure 2:**
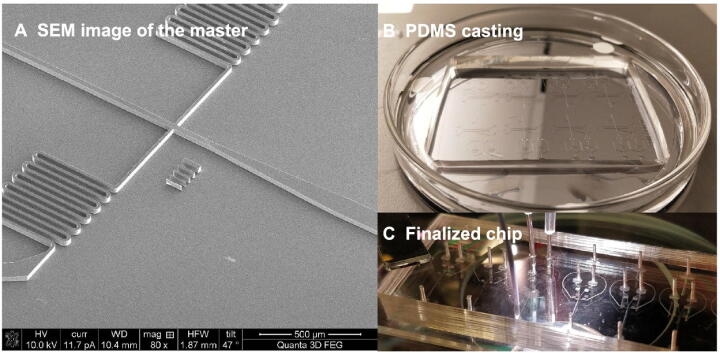
**Detecting the signal of cell fate commitment** The SGE value was calculated from Equation (5) based on MEF-to-neuron data (**A**), NPC-to-neuron data (**B**), hESC-to-DEC data (**C**), MHC-to-HCC data (**D**), and mESC-to-MP data (**E**), respectively. For each dataset, the significant increase of the SGE value (as shown as the red curve) indicates the imminent cell fate transition. Based on the top 5% genes with the highest and lowest local SGE values at the identified tipping point, t-SNE is applied to clustering cells for MEF-to-neuron data (**F**), NPC-to-neuron data (**G**), hESC-to-DEC data (**H**), MHC-to-HCC data (**D**), and mESC-to-MP data (**E**), respectively. Nodes in different colors represent cells from different time points. MEF, mouse embryonic fibroblast; NPC, neural progenitor cell; hESC, human embryonic stem cell; DEC, definitive endoderm cell; MHC, mouse hepatoblast cell; HCC, hepatocyte and cholangiocyte cell; mESC, mouse embryonic stem cell; MP, mesoderm progenitor; t-SNE, *t*-distributed stochastic neighbor embedding.

For MEF-to-neuron data, there is a significant increase of the SGE value from day 5 to day 20 (P=0.0168), as shown as the red curve in Figure 2**A**. This significant change of SGE value provides the early-warning signal to an upcoming cell fate transition after day 20. This computational result agrees with the observation in the original experiment, *i.e.*, the differentiation of mouse embryonic intermediate cells into the induced neuron occurs at day 22 [22]. Besides, to demonstrate the robustness of the proposed method in terms of the cells, the box plot of the cell-specific graph entropy was shown based on the samples of each time point. It is seen that the median values of the box plot in Figure 2A also present a clear signal for the tipping point, suggesting that the SGE value is featured with high robustness against the sample noise. Therefore, the signature of a critical transition from embryonic fibroblasts to neurons is identified by SGE at the single-cell resolution of the cell populations.

When applied to NPC-to-neuron data, *i.e.*, a 30-day time-course differentiation experiment of human neural progenitor cells into mature neurons (Figure 2B), there is a significant difference at day 1 (P=0.0362), suggesting a cell fate transition thereafter. This signal also coincides with the observation in the original experiment, that is, the cells at day 1 were the least heterogeneous, and after day 1 the transcriptional heterogeneity increased, reaching the largest heterogeneity among the neurons at day 30 eventually [23]. In addition, the median values of the box plot of the SGE value in Figure 2B also demonstrate the robust performance of scGET in detecting the early warning signal of a qualitative state transition.

For hESC-to-DEC data, the significant difference of the SGE value (P=0.0196) appears at 36 h (Figure 2C), which indicates an imminent cell fate transition after 36 h. Indeed, the differentiation induction into definite endoderm (DE) at 72 h, and the differentiation trajectory toward a DE fate commitment after 36 h, have been recorded before [15], [24], validating the SGE signals. The robustness of scGET in predicting the critical transition of the differentiation trajectory toward a DE fate can be shown by the median SGE values of the box plot.

As shown in Figure 2D, for MHC-to-HCC data, there is a significant difference at embryonic day 12.5 (E12.5) (P=7.3076E-05), after which hepatoblast-to-hepatocyte and cholangiocyte transition occurs [25]. Moreover, the median values of the box plot of SGE value in Figure 2D stably exhibit an obvious signal at the tipping point (E12.5), which demonstrates that scGET accurately predicts the cell fate transition for embryonic time-course differentiation.

scGET has been applied to mESC-to-MP data, which is obtained from an experiment of a retinoic acid (RA)-driven differentiation of pluripotent mESC to lineage commitment [26]. It is seen from Figure 2E, the mean SGE value reaches significant difference at 24 h (P=0.0288), signaling an upcoming critical transition after 24 h. Actually, there are cells exiting from pluripotency between 24 h and 48 h of RA exposure and then differentiating into endoderm around 48 h [26]. Further, the median values of the box plot of the SGE value in Figure 2E also indicate that the 24 h is a tipping point.

Besides, the data transformation from the gene expression matrix to the SGE matrix not only helps to detect the critical transitions of embryonic development but provides a way to perform temporal clustering analysis on cells during a biological process and thus explore the dynamic information of cell populations. The *t*-distributed stochastic neighbor embedding (t-SNE) is applied to carry out dimension reduction analysis and visualization [27]. A group of genes were selected, including the top 5% genes with the highest local SGE values and top 5% genes with the lowest local SGE values at the identified tipping point. We perform the clustering analysis over the selected genes using local SGE values (Equation (2)). For these five datasets of embryonic differentiation, as shown in Figure 2F–J, the clustering analysis based on the gene-specific local SGE values can distinguish the state of cells at different stages or time points. In addition, the heatmap of the local SGE values for the selected genes are stratified by the before-transition, the critical, and the after-transition states (see Figure S4 for details). Therefore, scGET performs well in cell clustering.

There is a pitfall in clustering analysis, that is, if we were to select features using a particular method on a set of samples, then subsequent clustering of the samples over these features should yield better segregation of the phenotype compared to features that were not selected in the same way [28]. Therefore, we carry out the comparison of clustering performance between the expression levels of DEGs and the local SGE values (Equation (2)) of the top high- and low-entropy genes. As shown in Figure S5, the local SGE values of top high- and low-entropy genes perform as well as the expression levels of DEGs in cell clustering, and the clustering details are also illustrated in Note 4 of File S1. Compared to the expression of DEGs, the SGE of signaling genes has better performance in terms of both accuracy and signal significance (Figure S6 and Note 5 of File S1).

### Inferring the dynamic evolution of gene regulatory networks

**Figure 3:**
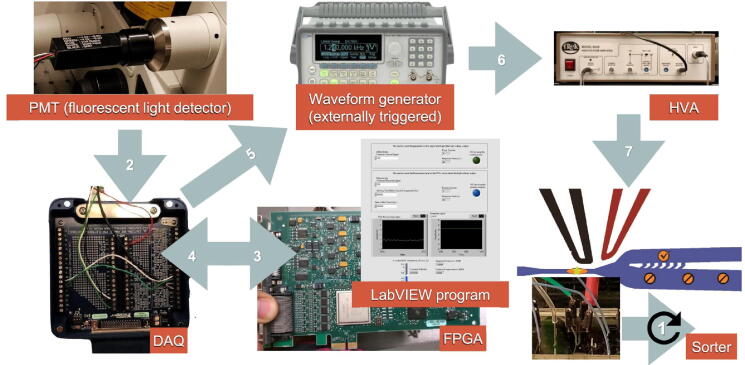
**Dynamic evolution of gene regulatory networks** Key gene regulatory networks were reconstructed using scGET for the signaling genes (top 5% genes with the largest local SGE value) based on scRNA-seq data. Color of each node represents the mean local SGE value (Equation (2)) and the color of each edge represents the statistical dependency index (r in Equation (1)). **A.** The dynamical evolution of gene regulatory networks for the MEF-to-neuron data. **B.** The dynamical evolution of gene regulatory networks for the MHC-to-HCC data. **C.** The dynamical evolution of gene regulatory networks for the hESC-to-DEC data. The landscape of local SGE values illustrates the dynamic evolution of network entropy in a global view for MEF-to-neuron data (**D**), MHC-to-HCC data (**E**), and hESC-to-DEC data (**F**), respectively.

At the identified transition point, the group of top 5% genes with the largest local SGE values (Equation (2)) were taken as the signaling genes for further functional and biological analysis. These signaling genes can be regarded as the DNB and may be highly associated with cell fate commitment during embryonic development. First, the signaling genes were mapped to the PPI network, from which the maximal connected subgraph was taken to study the dynamical network evolution. For MEF-to-neuron data, we show the dynamical evolution of signaling genes at the network level (Figure 3**A**). It is seen that a notable change of the network structure occurs at day 20, indicating an upcoming cell fate transition. The landscape of the local SGE value is illustrated in Figure 3D, and it is seen that the local SGE values of the signaling genes abruptly increase in a collective manner around day 20.

For MHC-to-HCC data (Figure 3B), there is an obvious change in the network structure at E12.5, betokening the cell fate transitions of the differentiation into HCC after E12.5 [25]. The overall dynamics of the signaling-gene network across all 7 time points are presented in Figure S7A. Therefore, the network signature of a critical transition during embryonic cell differentiation is illustrated, which may benefit the understanding of molecular associations among cell populations. Moreover, the landscape of local SGE values was presented in Figure 3E for a global view. It is found that the peak of local SGE values for signaling genes appeared at E12.5.

For hESC-to-DEC data, there is a drastic change in the network structure at 36 h (Figure 3C), indicating the cell fate transitions of the differentiation induction into DE at 72 h [24]. The dynamical evolution of the PPI network across all 6 time points is provided in Figure S7B. A description for the overall dynamic evolution of gene regulatory networks for MHC-to-HCC and hESC-to-DEC data is provided in Note 6 of File S1. Moreover, the landscape of local SGE value is presented in Figure 3F, showing that the peak local SGE of signaling genes appears at 36 h. Clearly, by exploring the dynamical change of gene associations, SGE offers an insight into critical transition during embryonic differentiation from the perspective of network dynamics.

We analyzed and discussed the topology of the built cell-specific networks. For example, from the MEF-to-neuron data, 405 cell-specific networks have been built. As shown in Table S1, it is found that the node degree distribution of 392 cell-specific networks follows the power law, *i.e.*, 96.5% of networks are scale-free (also see Note 7 of File S1 for details). Specifically, across the time points from day 0 to day 22, the overall network topology doesn’t change during the differentiation process, although the mean degree varies notably (from 2.75 at day 5 to 3.37 at day 20) at the identified tipping point (day 20), which is in accordance with the results above (Figure S8).

### Identifying the dark genes

**Figure 4:**
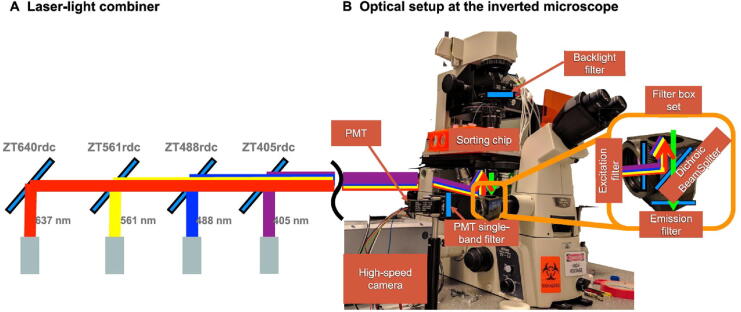
**The dark genes sensitive to SGE** The local SGE values (top) and gene expression levels (bottom) of dark genes for MEF-to-neuron data (**A**), MHC-to-HCC data (**B**), and hESC-to-DEC data (**C**) are provided.

In the biomedical field, DEGs are important in finding new biomarkers, regulators, and drug targets. However, some non-DEGs may also be involved in essential biological processes and should not be ignored. Previous studies have shown that such genes are enriched in key functional pathways and perform well in prognosis [12], and may play biological roles in endothelial cells [10]. During the analysis of the aforementioned single-cell datasets, some genes were discovered as the “dark genes”. These genes are non-differential in expression, but sensitive to SGE, that is, their local SGE values (Equation (2)) are significantly different (*P* < 0.05; *t*-test) between the critical point and non-critical points, but their expression levels change little. We performed the differential SGE analysis on the five embryonic time-course differentiation datasets. The change of SGE values and that of gene expression were compared based on the signaling genes (top 5% genes with the highest local SGE values). Figure 4 shows some randomly selected “dark genes” during MEF-to-neuron, MHC-to-HCC, and hESC-to-DEC processes. Other dark genes for MEF-to-neuron, MHC-to-HCC, and hESC-to-DEC processes were presented in Figure S9, Figure S10, and Figure S11, respectively, and dark genes of mESC-to-MP and NPC-to-neuron processes are provided in Figure S12 and Figure S13, respectively. These results show that for the dark genes, there are no significant differences at the gene expression level, but significant changes (*P* < 0.05; *t*-test) are observed at the SGE level. Some dark genes have been reported to be associated with embryonic development, suggesting that these genes may play important roles in embryonic development. The “dark genes” associated with embryonic development for MEF-to-neuron (Table S2), MHC-to-HCC (Table S3), and hESC-to-DEC (Table S4) processes are provided.

Among the dark genes with differential SGE values, 6 common signaling genes (CSGs) were identified for human embryo development from NPC-to-neuron and hESC-to-DEC data (Figure S14A) and 14 for mouse embryo development from the other three datasets (Figure S14B). To explore their function in embryo development, we performed the Reactome and KEGG pathway enrichment analysis for these CSGs.

For NPC-to-neuron data and hESC-to-DEC data, it has been confirmed that common dark genes, such as *LCOR* and *HLTF* (Figure 4C), play a relatively important role in embryonic differentiation. *LCOR*, an important molecule in the phosphatidylinositol signaling system, is a hub in the regulation of *TNFR1* signaling. The activation of *TNFR1* can further trigger multiple signal transduction pathways, such as the NF-κB signaling pathway, to control inflammation, cell proliferation, survival, or cell death. Therefore, multiple signal transduction pathways related to dark genes are triggered to induce inflammation, cell proliferation, survival, or cell death [29], [30], [31]. Besides, *LCOR* and *HLTF* together act as the Raf/MAP kinase cascade element in the Ras-Raf-MEK-ERK pathway to regulate the downstream MAPK1/MAPK3 signaling by directly activating MAP2K and MAPK, while MAPK3 and MAPK1 are phosphorylated by MAP2K1 and MAP2K2, thus responding to a wide range of extracellular stimuli, and promoting differentiation, proliferation, cell motility, cell survival, and some other important cellular behaviors [32], [33].

In addition, *LCOR* participates in T-cell factor (TCF) dependent signaling in response to the Wnt signal together with *MGA*. The Wnt pathway is one of the most important signaling pathways for cell proliferation, in which the binding of Wnt ligands to frizzled protein and lipoprotein receptor-related protein receptors leads to the destruction of complex inactivation, stabilization and nuclear translocation of β-catenin, and subsequent activation of TCF-dependent transcription [34], [35]. Transcriptional activation in response to Wnt signaling controls cell fate, stem cell proliferation, and self-renewal, as well as promotes tumorigenesis [34], [35], [36].

As an important transcription factor, *HLTF* is a key gene in both helicase and E3 ubiquitin ligase activities. We have noticed that it is directly involved in Ras activation upon Ca^2+^ influx through the N-methyl-D-aspartate receptor (NMDAR) [37]. Ras catalyzes its effector substrate to regulate a series of functions related to cell growth, differentiation, and apoptosis. Besides, *HLTF*, together with *MAG*, also plays important roles in the cell cycle. As described in the literature [23], *HLTF* is directly involved in the neurobiological process of negative regulation of NMDAR mediated neuronal transmission, which might also be one of the key regulators of brain/spinal neuron differentiation after 24 hours. It should be noted that these genes also play important roles in the signaling pathways related to cell proliferation. For example, *LCOR* and *HLTF* play a direct role in controlling the downstream MAPK pathway when they participate in the Raf/MAP kinase cascade signal cascade process. This kinase cascade, as a downstream effector of FLT3 signaling, communicates FLT3 signaling with the MAPK pathway. Beyond that, Raf/MAP kinase cascade is also important in cAMP responsive element binding protein 1 phosphorylation through NMDAR mediated activation of Ras signaling, thus leading to cell proliferation.

Among the 14 CSGs (as shown in Figure S14B) across MHC-to-HCC, MEF-to-neuron, and mESC-to-MP datasets, it has been seen that some genes, including *Polr2d*, *Atp6v1b2*, and *Cttn* (Figure 4A), are involved in mouse embryonic differentiation. *Polr2d* directly participates in RNA polymerase II (RNAPII) transcription initiation as the main component of RNAPII. The formation of an open complex exposes the template strand to the catalytic center of RNAPII. This promotes the formation of the first phosphodiester bond, initiating the transcription [38]. The initiation of transcription is the main regulatory point of gene expression. As well-known, in the absence of transcription, the development of early embryonic cells generally depends on the inherited mRNAs [39]. Together with the initiation of transcription, the embryonic cell enters the autologous development process. This is consistent with the pluripotent withdrawal process mentioned in a previous study [25], showing the essential role of DNA-directed RNAPII subunit RPB4 (POLR2D) in this process. Besides, v-ATPase subunit B2 (ATP6V1B2) is involved in the amino acid-activated mTOR receptor pathway, participating in a series of regulations including processing upstream amino acid stimulation signals, transmitting to the regulator, and then activating the downstream mTOR effector pathway [40]. The mTOR regulates neuronal proliferation, survival, growth, and function, which is crucial for the developmental process. Clear, deregulation of mTOR at any stage of development may have harmful consequences [41]. Thus, *Atp6v1b2* is of the potential function for cell fate determination, which is also shown in original experiment [22]. Meanwhile, *Cttn* is a component of cell tight junction, responding to extracellular pressure and stimulating actin assembly downstream. The actin assembly dynamics is strictly controlled by time and space [42], while the actin-assembled cytoskeleton has various physiological and pathological functions for cell migration, differentiation, embryonic development [43]. Therefore, *Cttn* may play an important role in embryonic development by regulating actin assembly.

### The underlying signaling mechanisms revealed by dark genes

**Figure 5:**
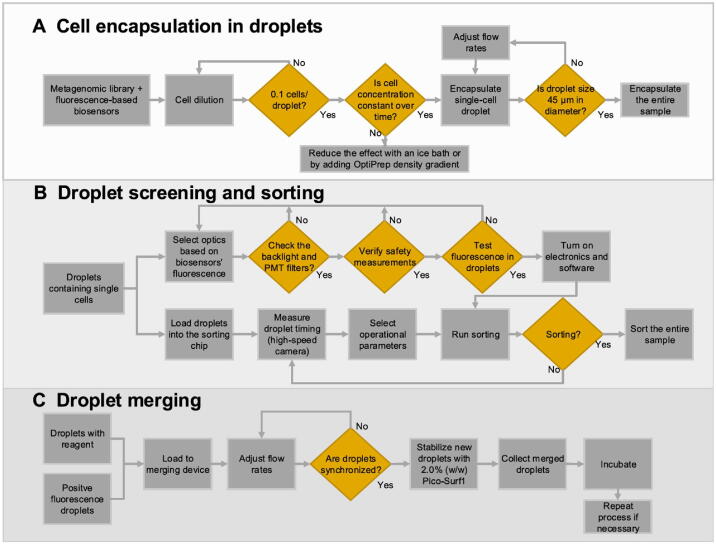
**Regulatory mechanisms of embryo development revealed by the dark genes** **A.** Dynamic changes of human CSG-associated network composed of 6 CSGs and their 175 first-order neighboring DEGs for hESC-to-DEC transition. **B.** Dynamical changes of CSG-associated network for MEF-to-neuron transition during mouse embryo development. **C.** KEGG pathway enrichment analysis for the dark genes (on the right) and their 175 neighboring DEGs (on the left) in hESC-to-DEC process. **D.** Switching dynamics of DEGs before and after critical point induced by upstream dark genes. Dark genes refer to the genes with non-differential expression (*P* ≥ 0.05; *t*-test) but differential SGE value (*P* < 0.05). CSG, common signaling gene.

To further analyze the regulation mechanisms underlying embryo development at the network level, the functional analysis was carried out on the PPI subnetworks of dark genes, which consist of the CSGs and their 1st-order neighbors from the PPI network. Thus, such a subnetwork is a CSG-associated network. For hESC-to-DEC process, the human CSG-associated network contains 6 CSGs and 175 1st-order DEG neighbors (Figure 5**A**). Out of the 14 CSGs across the mouse scRNA-seq datasets, there are 7 CSGs which can be mapped to the mouse PPI network. Thus, for MEF-to-neuron process, the mouse CSG-associated network contains 7 CSGs and 100 1st-order DEG neighbors (Figure 5B). It is seen that there is a significant “overturn” of gene expression for the networks after the critical transition, *i.e.*, gene expression changes significantly (*P* < 0.05; *t*-test) either from low to high, or *vice versa*.

The KEGG pathway enrichment analysis is performed to investigate the underlying mechanism of human dark genes and their neighboring DEGs (Figure 5C and D). Figure 5C shows that the dark genes are enriched in pathways closely related to embryo development. For instance, the MAPK signaling pathway regulates cell fate decisions in ESCs by controlling the proportion of cells differentiating along lineage [44]. Moreover, the PI3K/AKT signaling pathway regulates cell proliferation and differentiation in multiple cell types [45], [46].

Figure 5D shows the underlying mechanism revealed by the functional analysis on dark genes and their first-order neighbors. It is seen that the growth factors such as *IGF1* and ECM such as *LAMC2* and *COL4A1* are upstream regulators that drive cells into developmental critical transition during cell differentiation. Although the expression levels of dark genes change little during the critical transition, there is a significant overturn (*P* < 0.05; *t*-test) of the expression for some of their downstream genes. It is noticed that there is a signal chain that responds to the dark genes in the MAPK and PI3K/Akt signaling pathways, both of which are important for cell proliferation. In the MAPK pathway, an identified dark gene, *MAPK9*, is the key gene of the c-Jun N-terminal kinase subclass pathway and may induce a variety of upstream signals to cause cell proliferation and differentiation [47]. In addition, in PIK3/Akt signaling pathway, the upregulation of *IGF1R*, *SOS1*, and *SOS2* will activate Ras and further activate *RPS6KA3*, which may cause the mitogenic effect [48]. The downstream signal response caused by dark genes has a close relationship with the process of cell proliferation and differentiation. The accumulation of sustained expression of related genes in these pathways from 0 h to 36 h plays an important role in promoting proliferation and differentiation, which is consistent with the findings reported in the literature [24] that the identified tipping point may be an important time point to guide the development of pluripotent stem cells to DE.

## Discussion

Predicting a cell fate or lineage transition for cell differentiation is a task of biological and clinical importance [9]. Understanding such cell fate commitment may help to construct individual-specific disease models and design therapies with great specificity for complex diseases relevant to cell differentiation [49]. Most of the existing methods applied in analyzing scRNA-seq data are based on gene expression and its statistical quantities. However, gene expression levels of scRNA-Seq data are sometimes too fluctuating to characterize the dynamics of the biological processes [10]. In this study, from a cell-specific network viewpoint, we developed scGET to explore the dynamic information of gene–gene associations from scRNA-seq data and thus to construct the cell-specific networks in single-cell populations. Node degree in most cell-specific networks follows the power law, suggesting that the built networks are (or approximately are) scale-free. By transforming the sparse gene expression matrix from the scRNA-seq data into a non-sparse graph entropy matrix, scGET offers a computational insight into the network dynamics at the single-cell level. scGET has been applied to five scRNA-seq datasets and identified the critical stage or tipping point of the impending cell fate transition during early embryonic development. For instance, the significant change of the SGE value indicates the critical point (day 20) in the MEF-to-neuron data before the differentiation into induced neurons, the critical point (36 h) in the hESC-to-DEC data prior to the differentiation induction into DE, and the tipping point (E12.5) in the MHC-to-HCC data before the differentiation into hepatocytes and cholangiocytes. These results show that SGE also performs well in cell clustering of temporal information.

Besides, scGET helps to uncover the dark genes which are non-differential in their expression but sensitive to SGE. Such non-differential genes were often ignored by the traditional differential gene expression analyses. However, some non-differential genes may also be involved in the key biological activities of cells and play important roles in embryonic development [50], [51]. As illustrated by functional analysis, the up-regulated expression of some key genes in MAPK and PI3K/Akt signaling pathways, both of which are essential for cell proliferation and differentiation [52], [53], results from the synergy of dark genes and their downstream targets. Thus, some dark genes such as *IGF1* encoding growth factor and *LAMC2* and *COL4A1* encoding ECM are identified as upstream regulators for cell proliferation and may also be involved in other development processes.

Notably, scGET is model-free, that is, the SGE strategy requires neither feature selection nor model/parameter training. In summary, SGE opens a new way to predict a cell fate transition at the single-cell level, which is helpful in tracking cell heterogeneity and elucidating the molecular mechanism underlying embryonic cell differentiation by combining with statistics-based and dynamics-based data science [54], [55].

## Data availability

The source code of scGET is publicly available at https://github.com/zhongjiayuna/scGET_Project.

## Competing interests

The authors declare that they have no conflict of interest.

### CRediT authorship contribution statement

**Jiayuan Zhong:** Conceptualization, Formal analysis, Software, Methodology, Visualization, Writing – original draft, Writing – review & editing. **Chongyin Han:** Data curation, Formal analysis, Software, Visualization, Writing – original draft. **Xuhang Zhang:** Data curation, Visualization. **Pei Chen:** . **Rui Liu:** Conceptualization, Supervision, Methodology, Writing – original draft, Writing – review & editing, Project administration.
