# Supplementary material for: scGET: Predicting Cell Fate Transition During Early Embryonic Development by Single-cell Graph Entropy
Source: Genomics Proteomics Bioinformatics. 2021 Dec 24;19(3):461–74. doi: 10.1016/j.gpb.2020.11.008 (PMC8864248; doi:10.1016/j.gpb.2020.11.008)
Supplement: Supplementary File S1 [file mmc1.docx]

**File S1 Additional information of scGET**

**Note 1 Building the cell-specific network**

The process of constructing a cell-specific network is shown as following (see Figure S1 for detail). (i) we make scatter diagrams for every two genes, where each point represents a cell, and the vertical- and horizontal-axes are the expression values of the two genes in the $N$ cells. (ii) Near the cell $C_{k}$ (the point with red color), we make the light green and blue box to represent the neighborhood of $E_{i}^{(k)}$ and $E_{j}^{(k)}$ respectively. In our work, the neighborhood of the light green and blue box is drawn based on the predetermined number of cells ${(n}^{\left( k \right)}\left( E_{i} \right)=n^{\left( k \right)}(E_{j})=0.1N)$, where the $n^{\left( k \right)}(E_{i})$ and $n^{\left( k \right)}\left( E_{j} \right)$ represent the number of the points (cells) within the green box, blue box, respectively. (iii) The overlapping of two boxes (the light green and blue box) is the light red box, which represents the neighborhood of ($E_{i}^{(k)}$,$E_{j}^{(k)}$). The value of $n^{\left( k \right)}(E_{i},E_{j})$ can be obtained by counting the points (cells) in the red box. (iv) If the statistical dependency index *i.e.*,$r_{i,j}^{(k)}$ is greater than zero, there is an edge between $g_{i}$ and $g_{j}$ in the cell $C_{k}$, otherwise, there is no edge. In this way, we construct a cell-specific network $N^{(k)}$ for cell $C_{k}$.

**Note 2 Performance of three types of SGE scores generated from** $\boldsymbol{r}_{\boldsymbol{i,j}}^{\boldsymbol{(k)}}$*****$\boldsymbol{E}_{\boldsymbol{j}}^{\boldsymbol{(k)}}$**,** $\boldsymbol{r}_{\boldsymbol{i,j}}^{\boldsymbol{(k)}}$**, and** $\boldsymbol{E}_{\boldsymbol{j}}^{\boldsymbol{(k)}}$

To test how important the factor $r_{i,j}^{(k)}$ is in identifying the critical transition point, we calculated the local SGE scores

$H_{i}^{(k)}= -\frac{1}{\log(S)}\sum_{j=1}^{S} p_{i,j}^{(k)}\log(p_{i,j}^{(k)})$, (1)

which were calculated and tested in the single-cell RNA sequencing (scRNA-seq) embryonic differentiation datasets based on the following three different probability $p_{i,j}^{(k)}$, respectively.

(i) The probability in Equation (1) is generated from $r_{i,j}^{\left( k \right)}\cdot E_{j}^{\left( k \right)}$ (the combination of statistical dependency index $r_{i,j}^{(k)}$ and the cell’s gene expression value $E_{j}^{(k)}$), *i.e.*,

$p_{i,j}^{(k)}=\frac{r_{i,j}^{(k)}\cdot E_{j}^{(k)}}{\sum_{l\in N(i)} r_{i, l}^{(k)}\cdot E_{l}^{(k)}}$ , (2)

where $N\left( i \right)$represents the neighbors of the center gene $g_{i}.$

(ii) The probability in Equation (1) is generated from only the statistical dependency index $r_{i,j}^{(k)}$, *i.e.*,

$p_{i,j}^{(k)}=\frac{r_{i,j}^{(k)}}{\sum_{l\in N(i)} r_{i, l}^{(k)}}$ , (3)

where $N\left( i \right)$represents the neighbors of the center gene $g_{i}$.

(iii) The probability in Equation (1) is generated from just the cell's gene expression $E_{j}^{(k)}$, *i.e.*,

$p_{i,j}^{(k)}=\frac{E_{j}^{(k)}}{\sum_{l\in N(i)} E_{l}^{(k)}}$ , (4)

where $N\left( i \right)$represents the neighbors of the center gene $g_{i}.$ Comparing the SGE scores (Equation (1)) respectively from $r_{i,j}^{\left( k \right)}\cdot E_{j}^{\left( k \right)}$ (Equation (2)), just $r_{i,j}^{(k)}$ alone (Equation (3)), and just $E_{j}^{(k)}$ alone (Equation (4)), there are the following results.

First, for some datasets, the SGE score from $r_{i,j}^{\left( k \right)}\cdot E_{j}^{\left( k \right)}$ or from $r_{i,j}^{(k)}$ can detect the critical transition points, while that from $E_{j}^{\left( k \right)}$ fails. For the NPC-to-neuron data (Figures S3A−C), the SGE score from $r_{i,j}^{\left( k \right)}\cdot E_{j}^{\left( k \right)}$ or from $r_{i,j}^{(k)}$ indicates the tipping point by the significant increase ($P=0.0362$ for the $(r_{i,j}^{\left( k \right)}\cdot E_{j}^{\left( k \right)})$-based SGE; $P=0.0424$ for the $r_{i,j}^{(k)}$-based SGE, with the threshold/level of significance $P=0.05$), but that from simply just $E_{j}^{(k)}$ fails ($P=0.0772$ for the $E_{j}^{(k)}$-based SGE, with the threshold $P=0.05$). Another example is the mESC-to-MP data shown as in Figures S3D−F. The SGE score from $r_{i,j}^{\left( k \right)}\cdot E_{j}^{\left( k \right)}$ or from $r_{i,j}^{(k)}$ provides a qualified signal ($P=0.0288$ for the $(r_{i,j}^{\left( k \right)}\cdot E_{j}^{\left( k \right)})$-based SGE; $P=0.0407$ for the $r_{i,j}^{(k)}$-based SGE, with the threshold $P=0.05$), but that from simply just $E_{j}^{(k)}$ fails ($P=0.0552$ for the $E_{j}^{(k)}$-based SGE, with the threshold $P=0.05$).

Second, although the SGE scores from $r_{i,j}^{\left( k \right)}\cdot E_{j}^{\left( k \right)}$, $r_{i,j}^{(k)}$, and $E_{j}^{\left( k \right)}$ all indicate the tipping point, the signal from the $(r_{i,j}^{\left( k \right)}\cdot E_{j}^{\left( k \right)})$-based SGE score is the most significant. In Figures S3G−I, the SGE signal yielding from using $r_{i,j}^{\left( k \right)}\cdot E_{j}^{\left( k \right)}$ or just $r_{i,j}^{(k)}$ is more significant ($P=0.0196$ for the $(r_{i,j}^{\left( k \right)}\cdot E_{j}^{\left( k \right)})$-based SGE score; $P=0.0199$ for the $r_{i,j}^{(k)}$-based SGE score) than that from using just $E_{j}^{(k)}$ ($P=0.0380$ for the $E_{j}^{(k)}$-based SGE score). A similar result was shown in Figures S3J−L that the $(r_{i,j}^{\left( k \right)}\cdot E_{j}^{\left( k \right)})$-based SGE signal ($P=7.3076E-05$) and the $r_{i,j}^{(k)}$-based SGE signal ($P=0.0016$) are more significant than the $E_{j}^{(k)}$-based SGE signal ($P=0.0279$).

Third, the SGE signal from $r_{i,j}^{\left( k \right)}\cdot E_{j}^{\left( k \right)}$ better agrees with the observation from the original experiment. For MEF-to-neuron data as shown in Figures S3M−O, the tipping point identified by SGE scores from $r_{i,j}^{\left( k \right)}\cdot E_{j}^{\left( k \right)}$ or just $r_{i,j}^{(k)}$ is day 20 ($P=0.0168$ for the $(r_{i,j}^{\left( k \right)}\cdot E_{j}^{\left( k \right)})$-based SGE score; $P=0.0228$ for the $r_{i,j}^{(k)}$-based SGE score), while the tipping point identified by the $E_{j}^{(k)}$-based SGE score can be roughly regarded as appearing at day 5 ($P=0.0435$ for the $E_{j}^{(k)}$-based SGE score). In the original experiment, the differentiation of mouse embryonic intermediate cells into induced neuron occurs at day 22 [22], immediately after the identified tipping point (day 20) by the $(r_{i,j}^{\left( k \right)}\cdot E_{j}^{\left( k \right)})$-based SGE score.

Therefore, the *r*-factor ($r_{i,j}^{(k)}$) plays an essential role in the proposed scGET, while the cell’s gene expression value $E_{j}^{(k)}$ is also important. Therefore, in this study, we combined $r_{i,j}^{(k)}$ with $E_{j}^{(k)}$ (*i.e.*, $r_{i,j}^{\left( k \right)}\cdot E_{j}^{\left( k \right)}$) to generate the SGE score, which has a better performance in analyzing the dynamic changes of cell-specific networks than using $r_{i,j}^{(k)}$ alone or $E_{j}^{(k)}$ alone.

**Note 3 Description of the five datasets**

To demonstrate the performance of the proposed computational method, we applied scGET to five scRNA-Seq datasets from Gene Expression Omnibus (GEO) database (http://www.ncbi.nlm.nih.gov/geo/). The detailed description and sources of the datasets are listed as follows.

MEF-to-neuron data [22] is generated from a reprogramming from mouse embryonic fibroblasts (MEF) to induced neuronal cells. There are 405 single cells and eleven cell types in total. MEF was induced at day 0 (73 cells). Samples are also taken at day 2 (128 cells), day 5 (55 cells), day 20 (33 cells), and day 22 (116 cells) after induction. The normalized data were derived from GEO under accession number GSE67310.

NPC-to-neuron data [23] presents a non-directed differentiation over a 30-day period of neural progenitor cells (NPCs) into developing neurons. There are 483 single-cell samples and six cell types in total. Cell numbers are captured at day 0 (80 cells), day 1 (78 cells), day 5 (85 cells), day 7 (80 cells), day 10 (79 cells), and day 30 (81 cells). The normalized data were obtained from GEO under accession number GSE102066.

hESC-to-DEC data [24] describes a time-course of human embryonic stem cells (hESCs) from the pluripotent state though a mesendoderm intermediate to definitive endoderm cells (DECs). This dataset includes 758 single cells and six cell types. A total of 758 cells are taken and profiled by scRNA-seq at 0 h (92 cells), 12 h (102 cells), 24 h (66 cells), 36 h (172 cells), 72 h (138 cells), and 96 h (188 cells). The normalized data were downloaded from GEO under accession number GSE75748.

MHC-to-HCC data [25] is generated from a study on the differentiation of mouse hepatoblasts cells (MHCs) into hepatocytes and cholangiocytes cells (HCCs). After quality control, a total of 447 cells with 7 time points are obtained during embryonic development, which includes 54 single cells at embryonic day 10.5 (E10.5), 70 at E11.5, 41 at E12.5, 65 at E13.5, 70 at 14.5, 77 at 15.5 and 70 at E17.5. The normalized data could be derived from GEO under accession number GSE90047.

mESC-to-MP data [26] describes retinoic acid-driven mouse embryonic stem cells (mESCs) differentiation from pluripotency to lineage commitment. A total of 339 single-cell samples are taken at 4 time points during mouse embryonic differentiation, which consists of 82 cells at 0 h, 86 cells at 12 h, 89 cells at 24 h, and 82 cells at 48 h. The normalized data were available from GEO under accession number GSE79578

**Note 4 Discussion on clustering performance between the expression levels of DEGs and SGE values of signaling genes**

We selected the top 5% most significant DEGs at the critical point and performed clustering analysis of the cells over these genes using expression values, that is, the *t*-distributed stochastic neighbor embedding [27] was applied to the dimension-reduction analysis and visualization for these genes. For MEF-to-neuron, MHC-to-HCC, hESC-to-DEC, NPC-to-neuron, and mESC-to-MP data, as shown in Figures S5A, C, E, G, and I, the clustering analysis over DEGs can distinguish the state of cells at different time points based on the expression. The DEGs work well in clustering analysis using expression values. In our study, for each cell in the tipping point, the genes with the top 5% largest local SGE value were selected. Then the selected genes were ranked by their frequency. The top 5% highest frequency genes were viewed as top high-entropy genes. The top 5% low-entropy genes were obtained using a similar procedure. We carried out the clustering analysis on this group of selected genes composed of the top high- and low-entropy genes. It can be seen from Figures S5B, D, F, H, and J that selected genes also have a good performance on clustering analysis using the SGE value. In summary, the local SGE values of the top high- and low-entropy genes perform as well as the expressions of DEGs in cell clustering

**Note 5 Discussion on predicting critical transitions between the SGE values of signaling genes and expression levels of DEGs**

The top DEGs were selected at each time point. Based on the DEGs, the expressions were applied to describing the dynamic changes of the cells. Specifically, we selected the DEGs to analyze the dynamic changes in terms of gene expression. At each time point, the mean of the top 5% most significant DEG expression is taken to predict any possible transition points. For five scRNA-seq embryonic differentiation datasets, as shown in the blue curves in Supplementary Figure S6, the expressions of DEGs illustrate the dynamic changes of some datasets. However, from the perspective of both accuracy and signal significance, the mean expression of DEGs performs worse than the results of the SGE values (the red curves in Figure S6). For one thing, for MEF-to-neuron data, NPC-to-neuron data, hESC-to-DEC data, and MHC-to-HCC data, the signal yielding from the SGE value is stronger than that from the expression of DEGs (Figures S6A−H). For another, for mESC-to-MP data, DEG expression fails to detect the signal of a critical transition (Figure S6J).

**Note 6 Description for the overall dynamic evolution of gene regulatory networks for MHC-to-HCC and hESC-to-DEC data**

At the identified transition point, we selected the top 5% genes with the largest local SGE values as the signaling genes. These signaling genes can be regarded as the dynamical network biomarker and may be highly associated with cell fate commitment during embryonic development. First, the signaling genes were mapped to the PPI network, from which the maximal connected subgraph was taken to study the dynamical network evolution. In such networks, each edge between genes $g_{i}$ and $g_{j}$ was reweighted by the mean value of the statistical dependency index $r_{i,j}^{(k)}$ (Equation (1)) across cells $C_{k}$ at a certain stage. For MHC-to-HCC data, to show the dynamical evolution of these signaling genes at the network level, the dynamical evolution of the network composed by signaling gene across all 7 time points is as shown in Figure S7A. There is an obvious change in the network structure at E12.5, signaling the cell fate transitions of the differentiation into hepatocytes and cholangiocytes after embryonic day 12.5 (E12.5) [25]. For hESC-to-DEC data, the whole dynamics of the signaling-gene network across all 6 time points are presented in Figure S7B, and there is an obvious change in the network structure at 36h, signaling the cell fate transitions of the differentiation induction into definite endoderm at 72 h [24].

**Note 7 Topological features of cell-specific networks**

For MEF-to-neuron data, we have analyzed the topology of the built cell-specific networks according to the definition of a scale-free network. In a scale-free network, the degree of the nodes obeys the power law [S1, S2]: the fraction of nodes with degree $k$ is proportional to $k^{-\gamma}$:

$P\left( k \right)\sim k^{-\gamma}$ (5)

with exponent $\gamma$ in the range $\gamma>2$.

For each cell in MEF-to-Neurons data, the exponent $\gamma$ of its cell-specific network was shown in the following Table S1. The degree distribution follows a power law (the exponent $\gamma>2$) for these 405 cell-specific networks, with 96.5% (392 scale-free networks and 13 other networks).

At each time point, the mean exponent $\gamma$ was calculated to show how the topology of scale-free cell-specific networks changes during differentiation. The degree distribution for these five time points was shown in Figure S8. It is seen that there is a significant change of the degree distribution at day 20 (the critical point), that is, at day 20 the degree of most nodes is around $k=40$, while at other time points, the degree of most nodes below $k=20$. In summary, the degree of the nodes in most cell-specific networks follows the power law, inferring that the built networks are (or approximately are) scale-free. Besides, the overall topology of such cell-specific networks doesn’t change during the differentiation process, though the mean degree may vary from time to time.

**References**

[S1] Goh KI, Kahng B, Kim D.. Universal behavior of load distribution in scale-free networks. Phys Rev Lett 2001;87:278701.

[S2] Jäger G, Hofer C, Kapeller M, Füllsack M. Hidden early-warning signals in scale-free networks. PLoS One 2017;12:e0189853.
