# Supplementary material for: scGET: Predicting Cell Fate Transition During Early Embryonic Development by Single-cell Graph Entropy
Source: Genomics Proteomics Bioinformatics. 2021 Dec 24;19(3):461–74. doi: 10.1016/j.gpb.2020.11.008 (PMC8864248; doi:10.1016/j.gpb.2020.11.008)
Supplement: Supplementary Figure S1 [file mmc2.pdf]

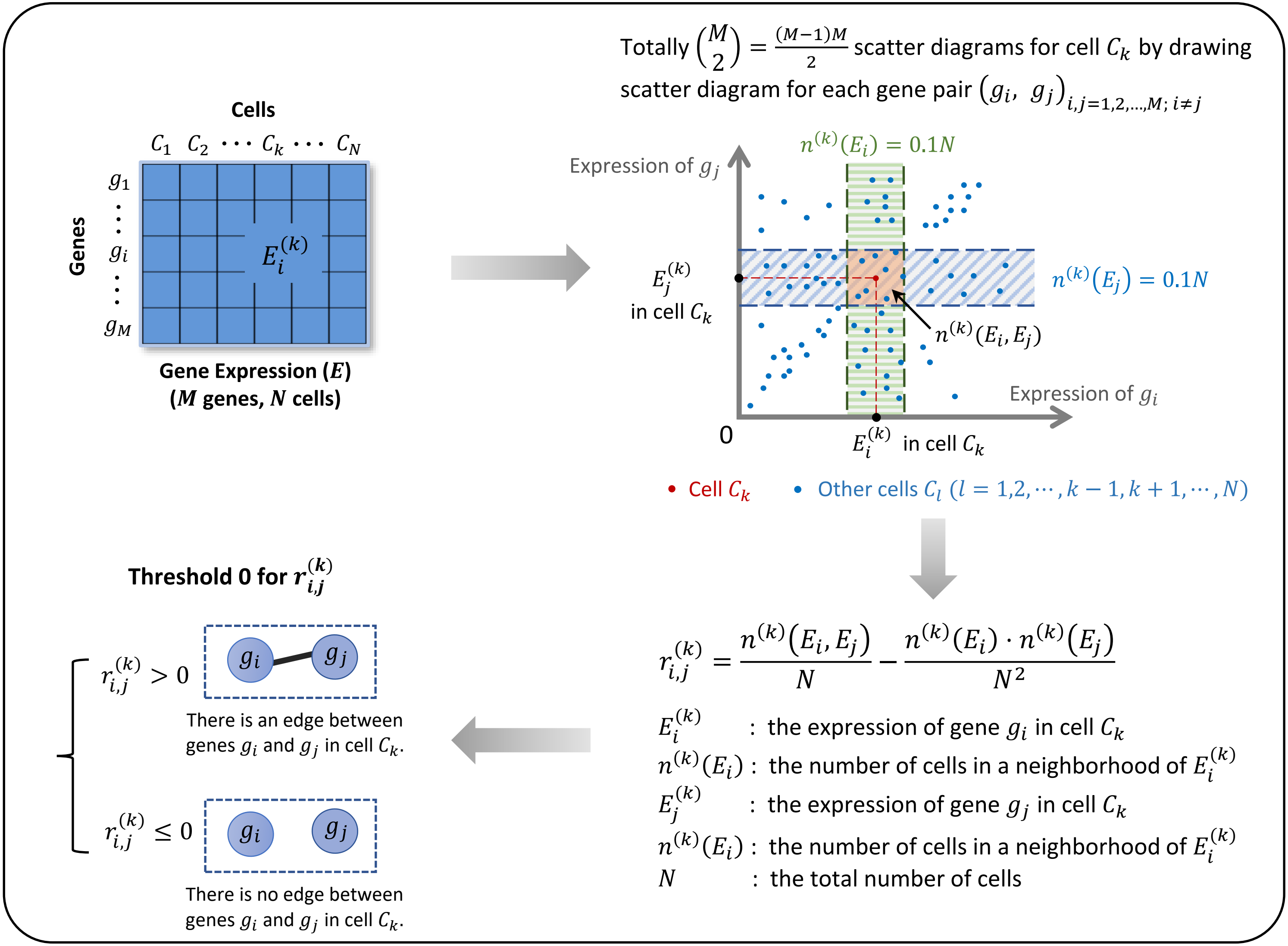

Totally $\binom{M}{2} = \frac{(M-1)M}{2}$ scatter diagrams for cell C_k by drawing scatter diagram for each gene pair $(g_i,\ g_j)_{i,j=1,2,\ldots,M;\ i\neq j}$
Cells
C_1 C_2 $\cdots$ C_k $\cdots$ C_N
Genes
g_1 $\vdots$ g_i $\vdots$ g_M
$E_i^{(k)}$
Gene Expression (E)
(M genes, N cells)
$n^{(k)}(E_i) = 0.1N$
Expression of g_j
$E_j^{(k)}$ in cell C_k
$n^{(k)}(E_j) = 0.1N$
$n^{(k)}(E_i, E_j)$
Expression of g_i
0
$E_i^{(k)}$ in cell C_k
Cell C_k
Other cells C_l $(l = 1,2,\cdots,k-1,k+1,\cdots,N)$
$r_{i,j}^{(k)} = \frac{n^{(k)}(E_i, E_j)}{N} - \frac{n^{(k)}(E_i)\cdot n^{(k)}(E_j)}{N^2}$
$E_i^{(k)}$: the expression of gene g_i in cell C_k
$n^{(k)}(E_i)$: the number of cells in a neighborhood of $E_i^{(k)}$
$E_j^{(k)}$: the expression of gene g_j in cell C_k
$n^{(k)}(E_i)$: the number of cells in a neighborhood of $E_i^{(k)}$
N : the total number of cells
Threshold 0 for $r_{i,j}^{(k)}$
$r_{i,j}^{(k)} > 0$
g_i g_j
There is an edge between genes g_i and g_j in cell C_k.
$r_{i,j}^{(k)} \leq 0$
g_i g_j
There is no edge between genes g_i and g_j in cell C_k.
