# Supplementary figures and images for: scGET: Predicting Cell Fate Transition During Early Embryonic Development by Single-cell Graph Entropy
Source: Genomics Proteomics Bioinformatics. 2021 Dec 24;19(3):461–74. doi: 10.1016/j.gpb.2020.11.008 (PMC8864248; doi:10.1016/j.gpb.2020.11.008)

## MEF-to-neuron data

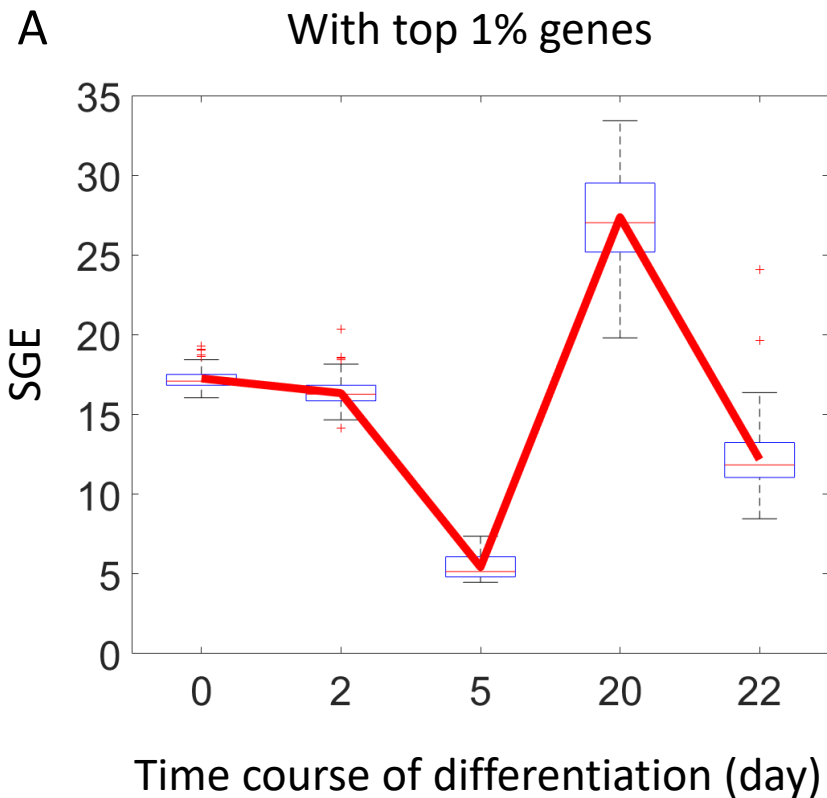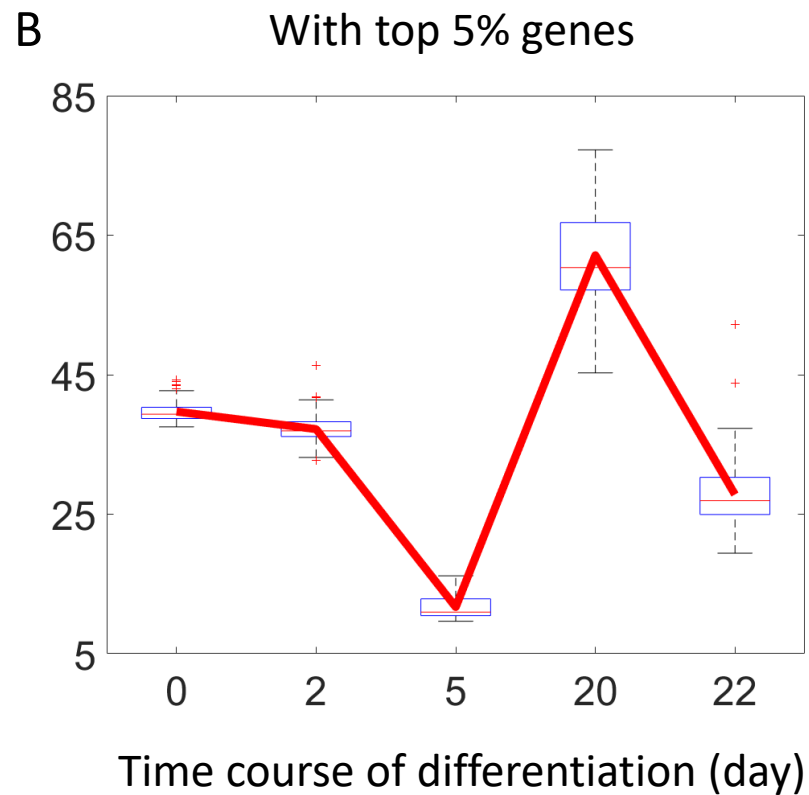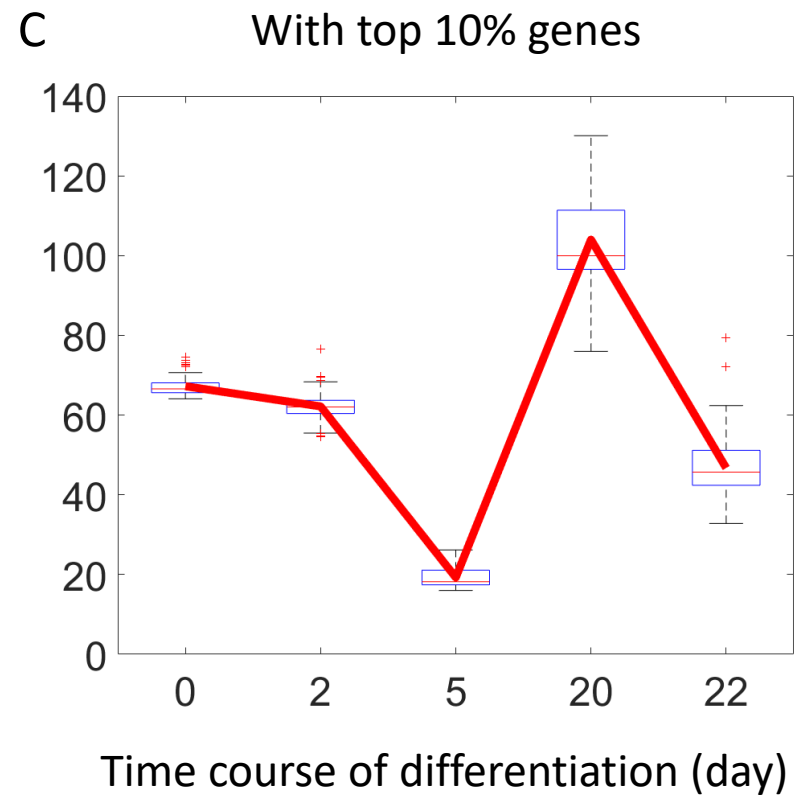

Supplement: Supplementary Figure S2 [file mmc3.pdf]

## NPC-to-neuron

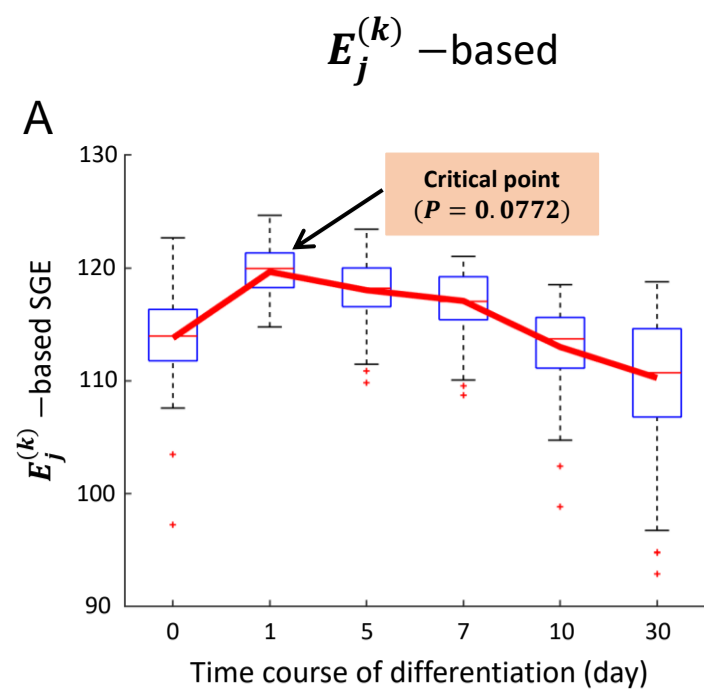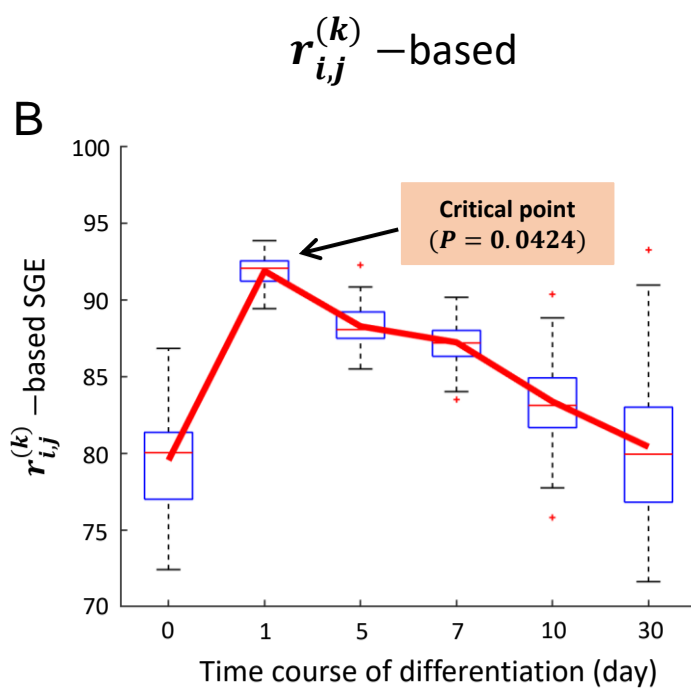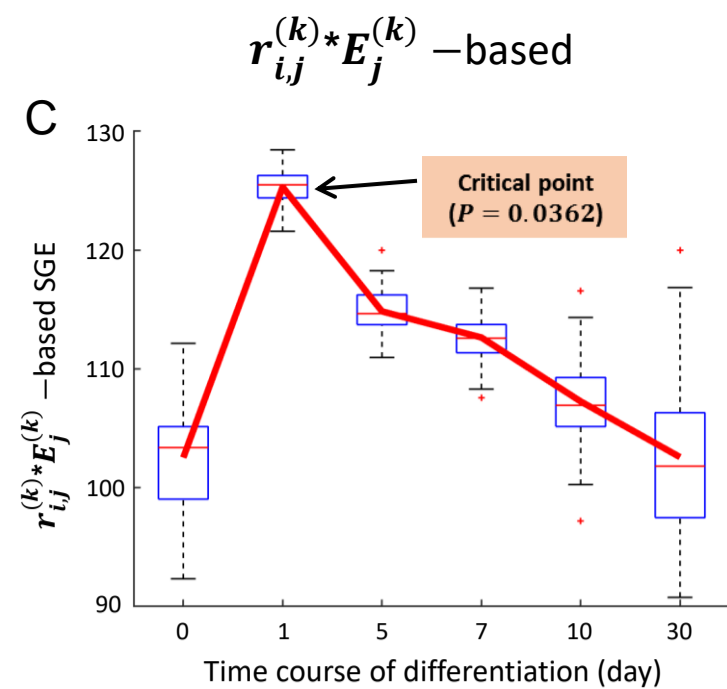

## mESC-to-MP

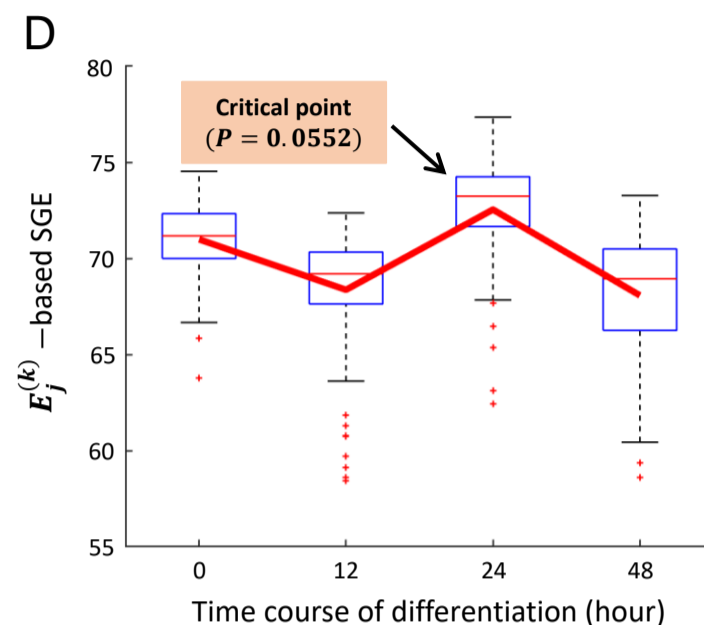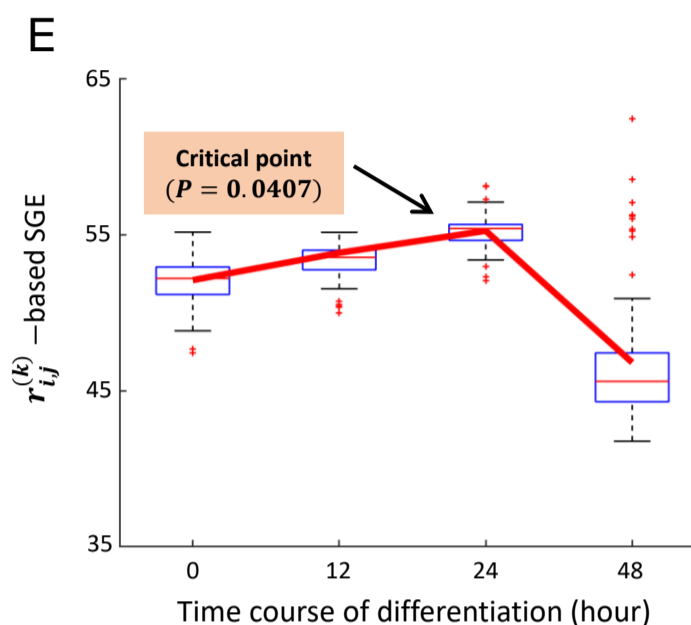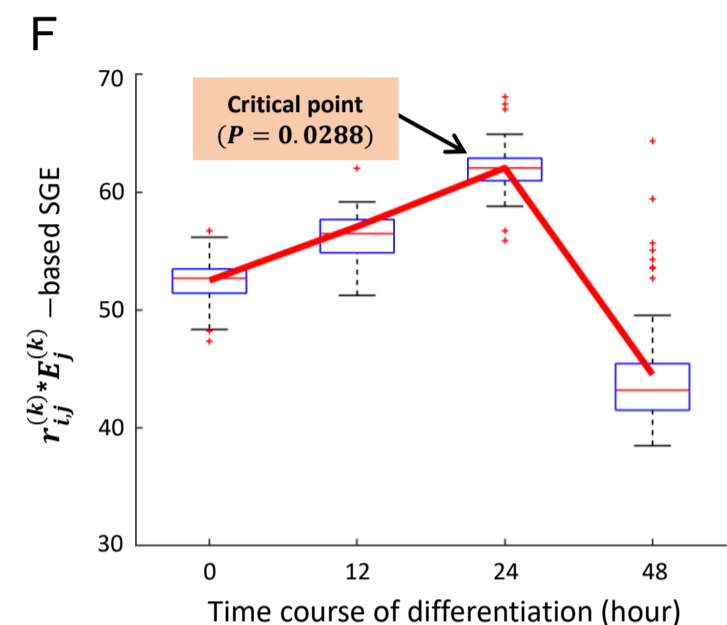

## hESC-to-DEC

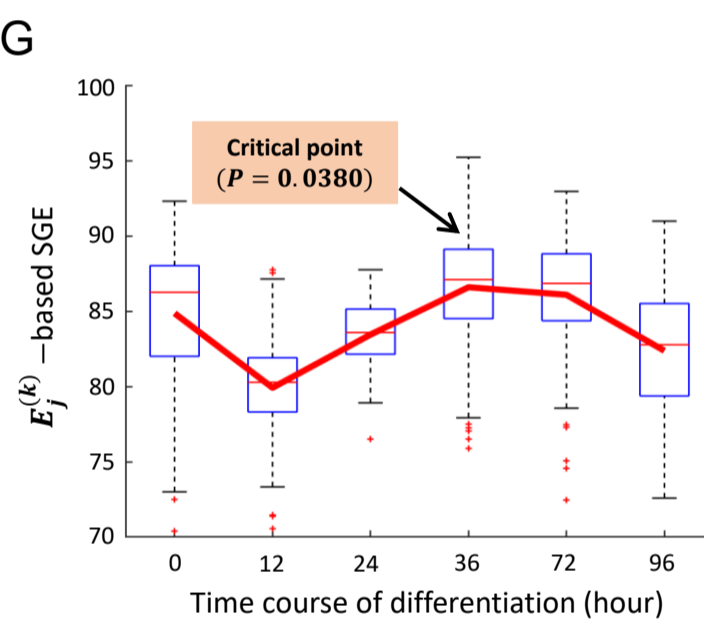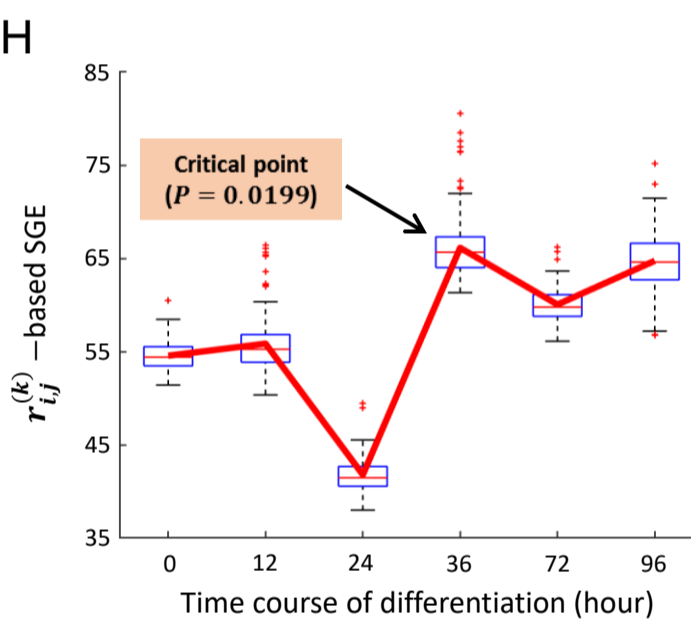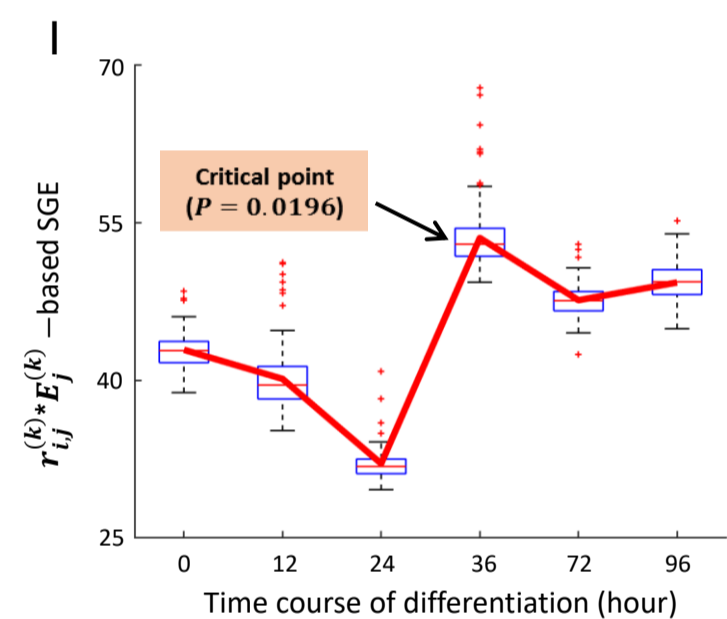

## MHC-to-HCC

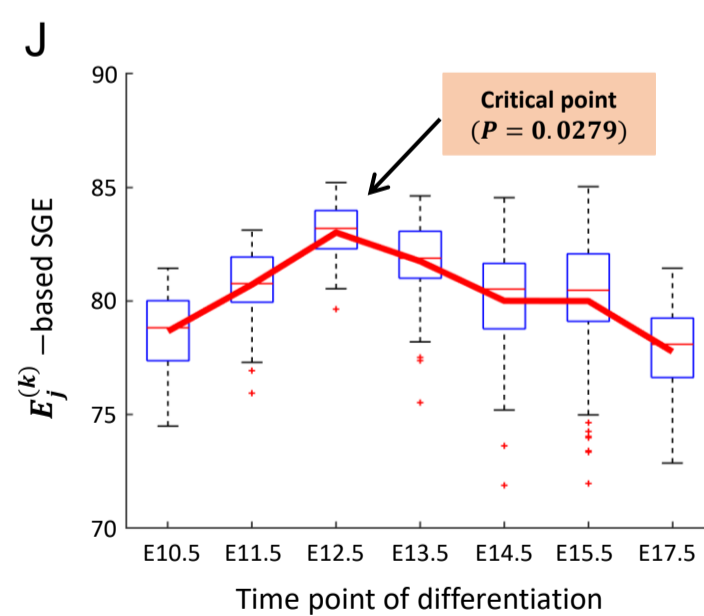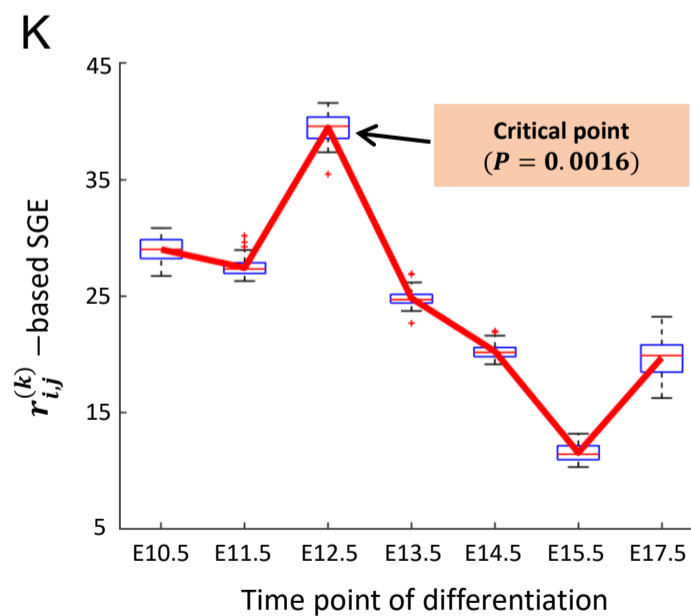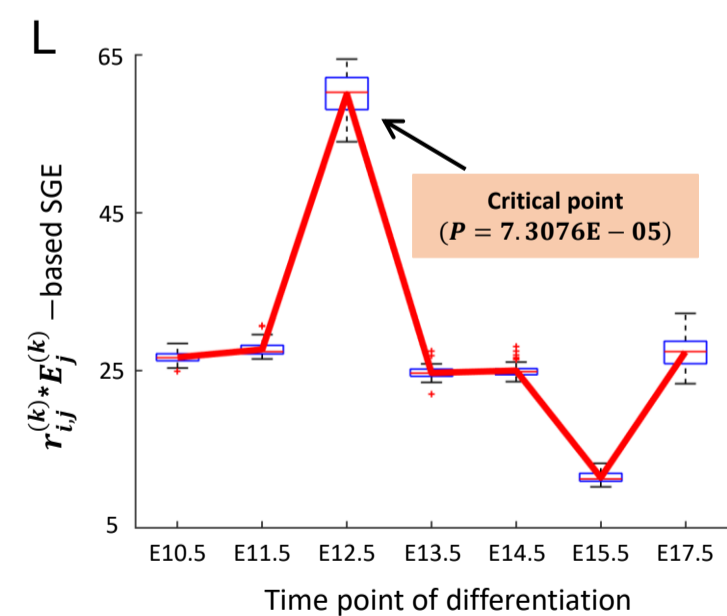

## MEF-to-neuron

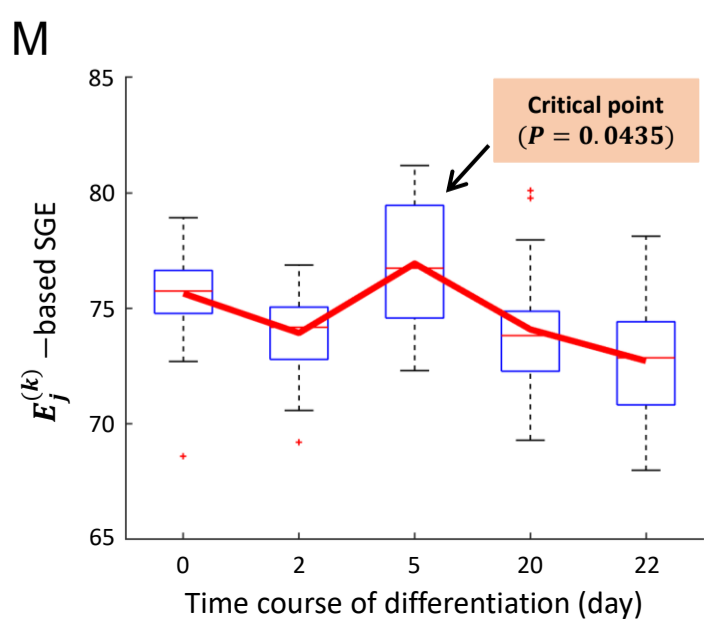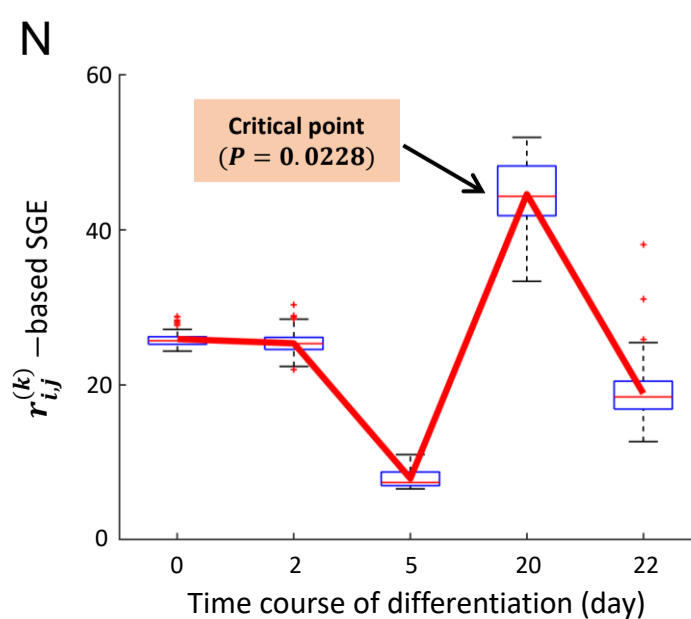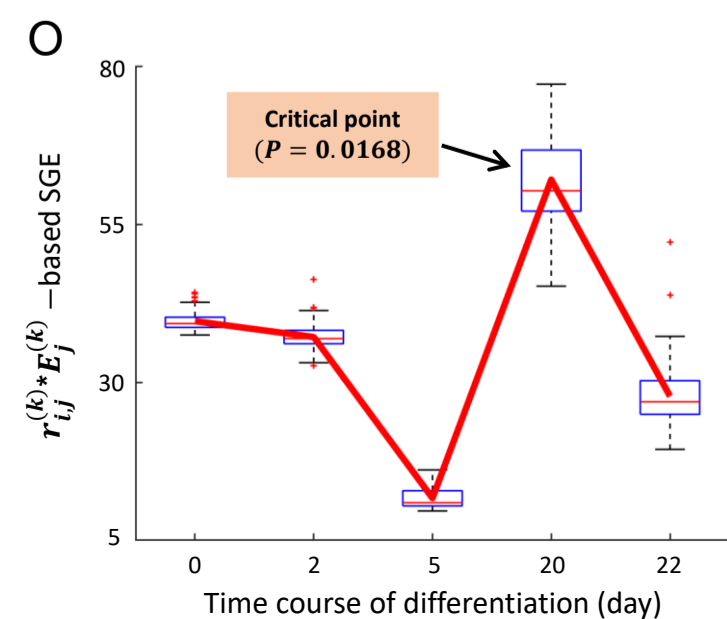

Supplement: Supplementary Figure S3 [file mmc4.pdf]

A

hESC-to-DEC data

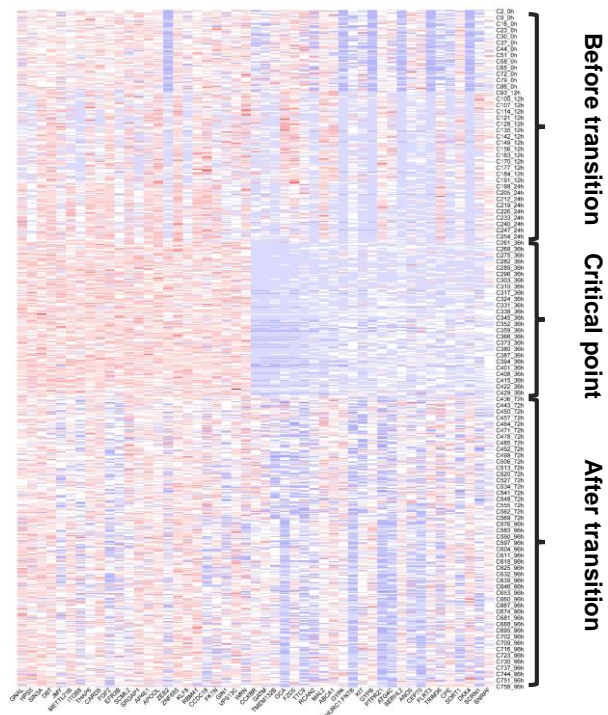

B

MEF-to-neuron data

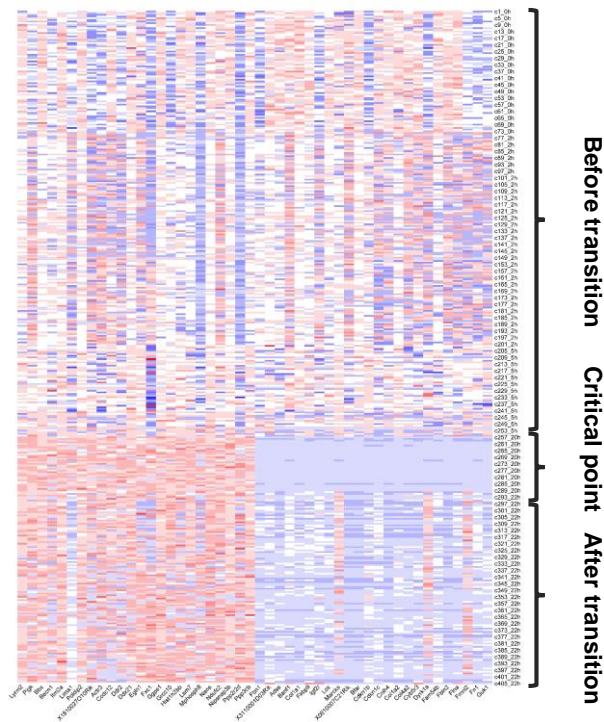

C

mESC-to-MP data

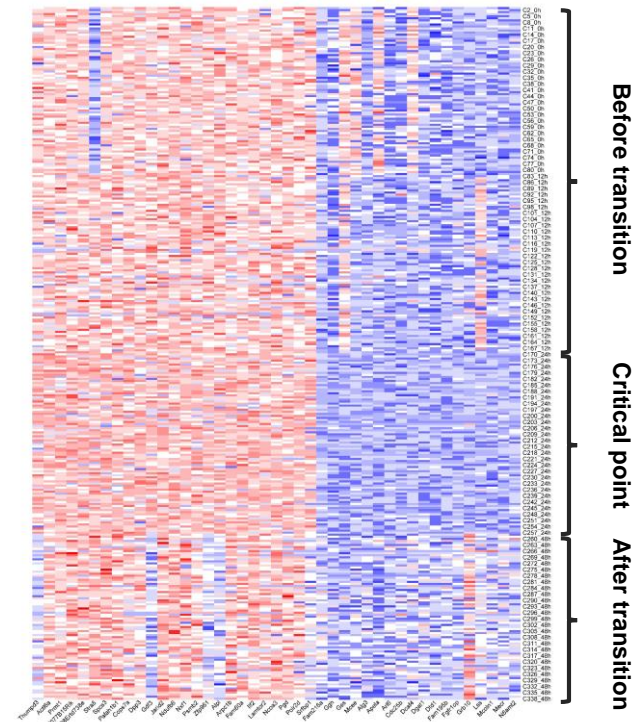

D

MHC-to-HCC data

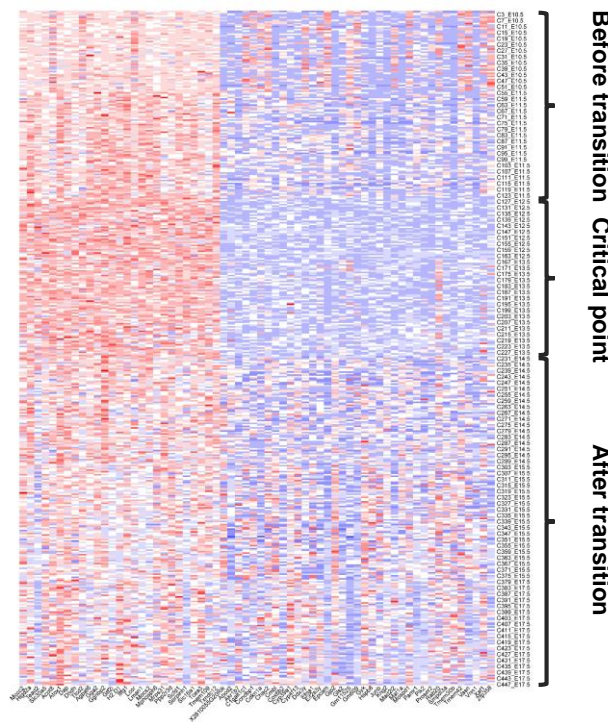

E

NPC-to-neuron data

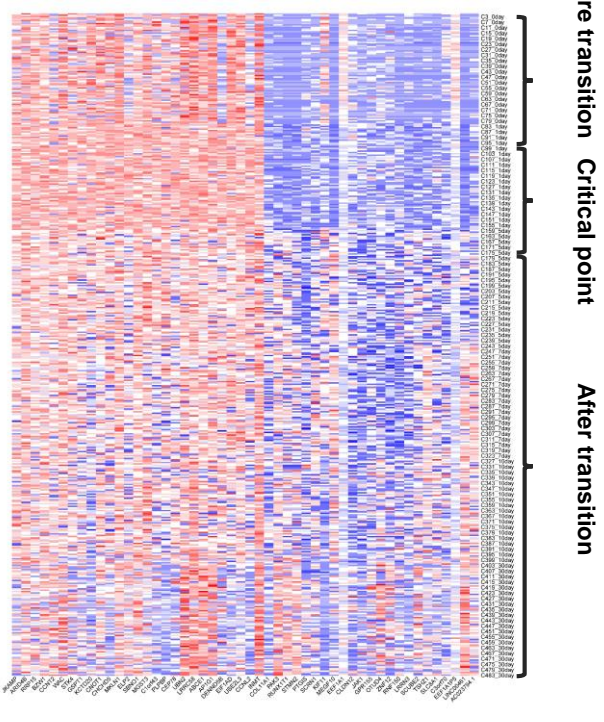

A

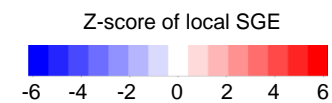

B

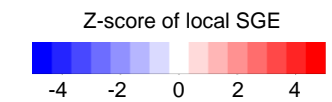

C

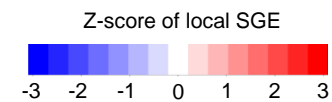

D

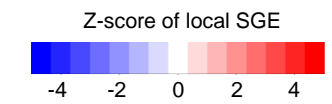

E

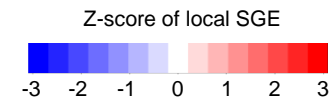

Supplement: Supplementary Figure S4 [file mmc5.pdf]

Based on expression of DEGs

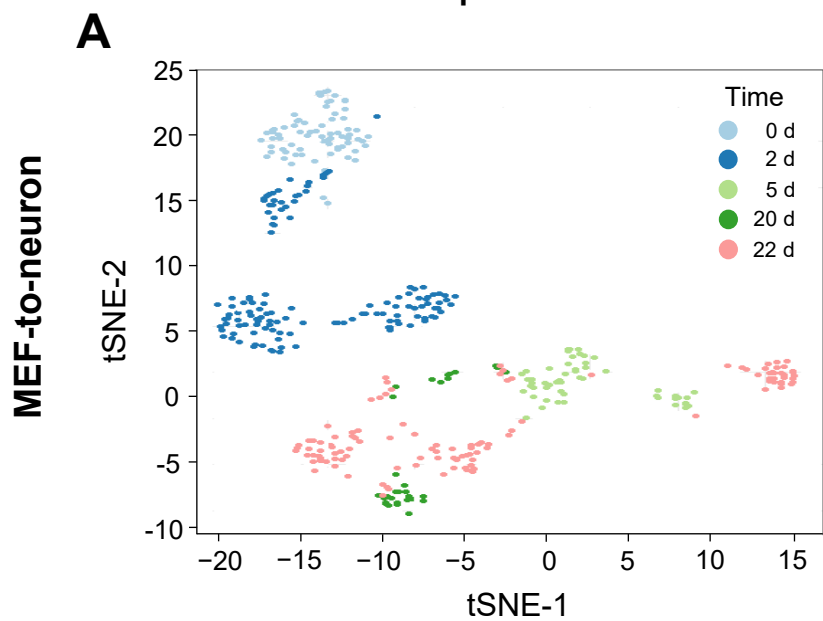

Based on local SGE of selected genes

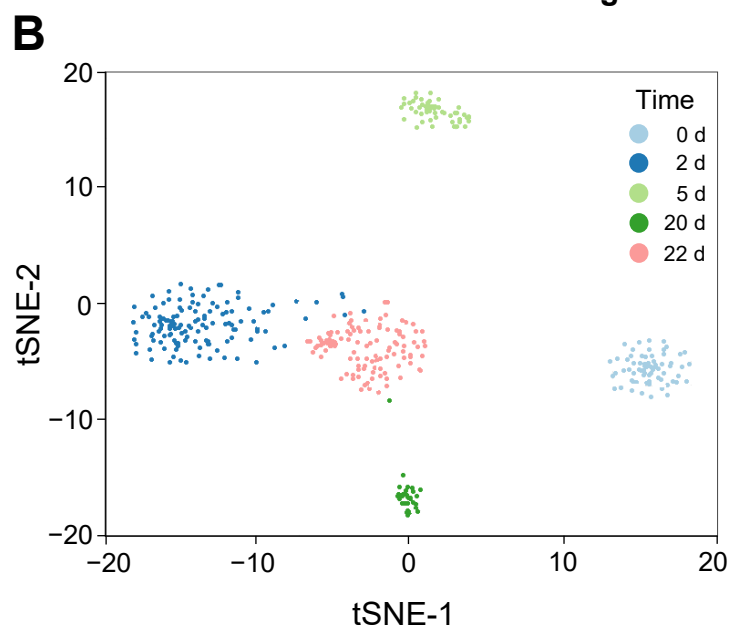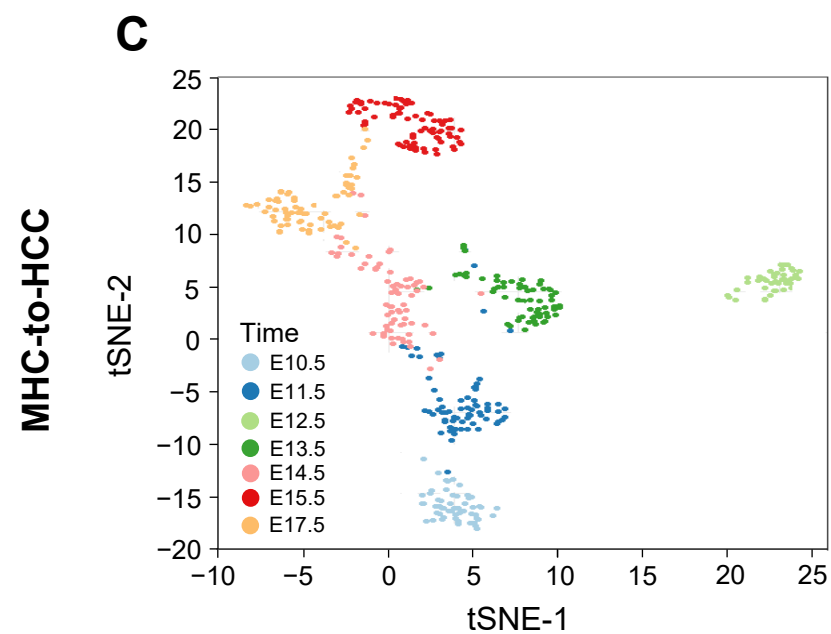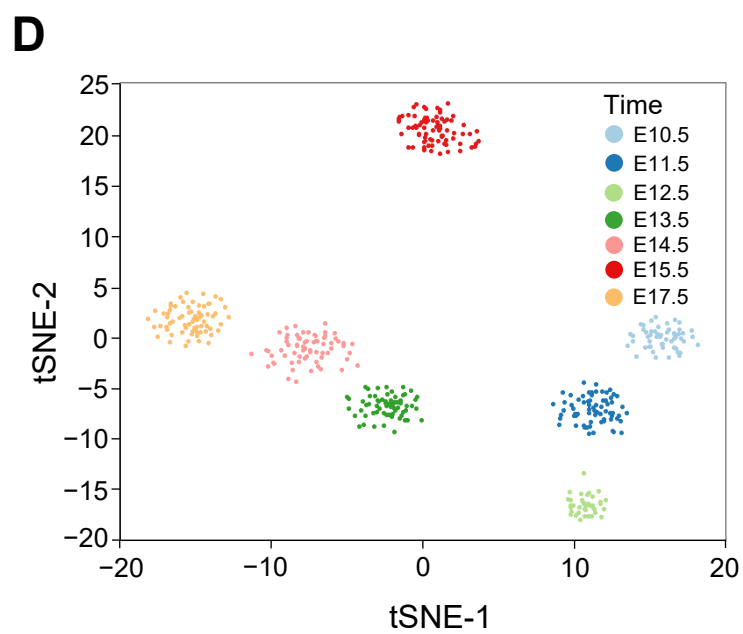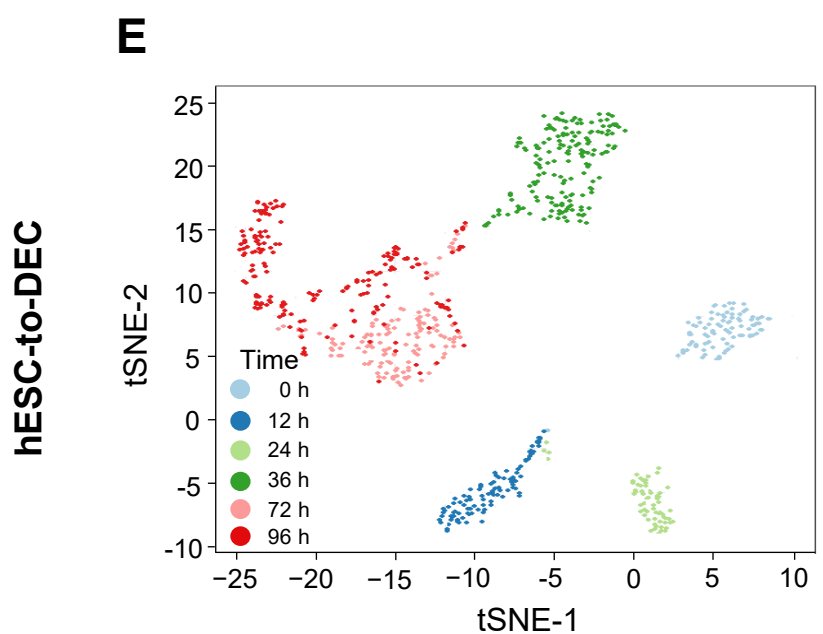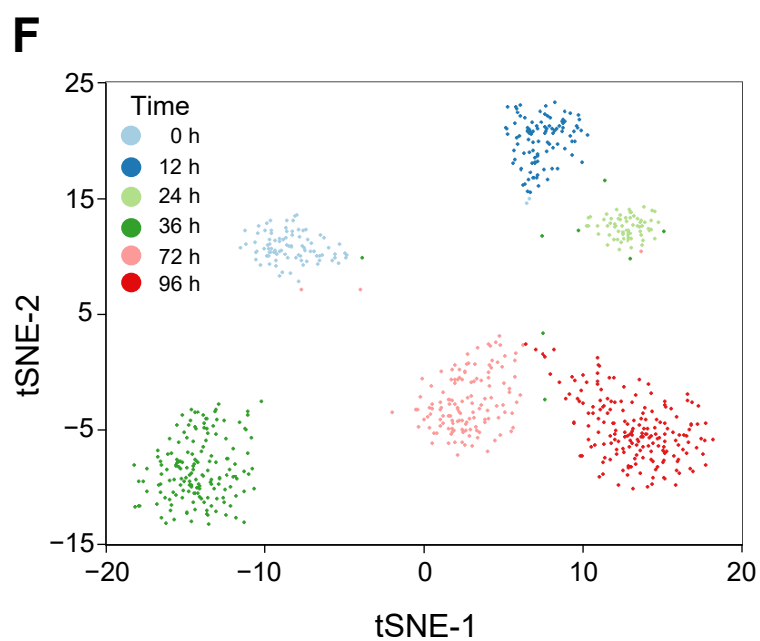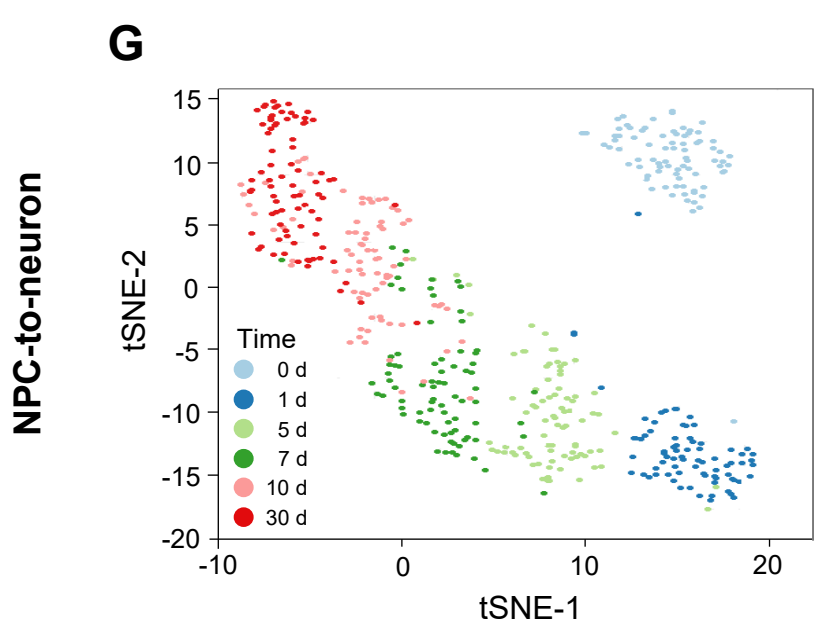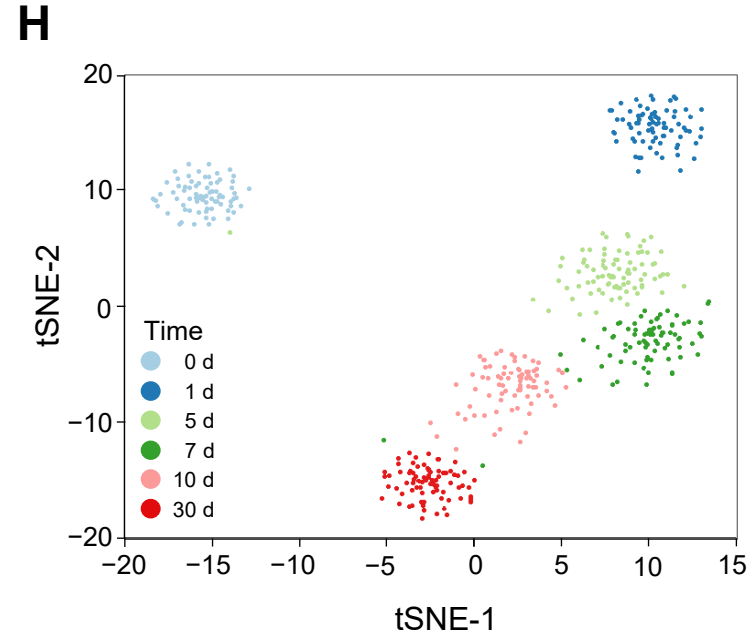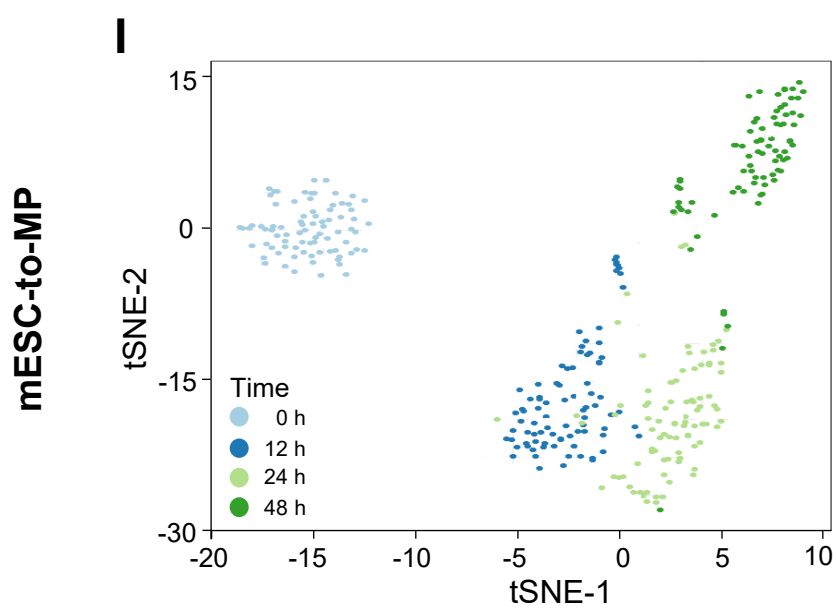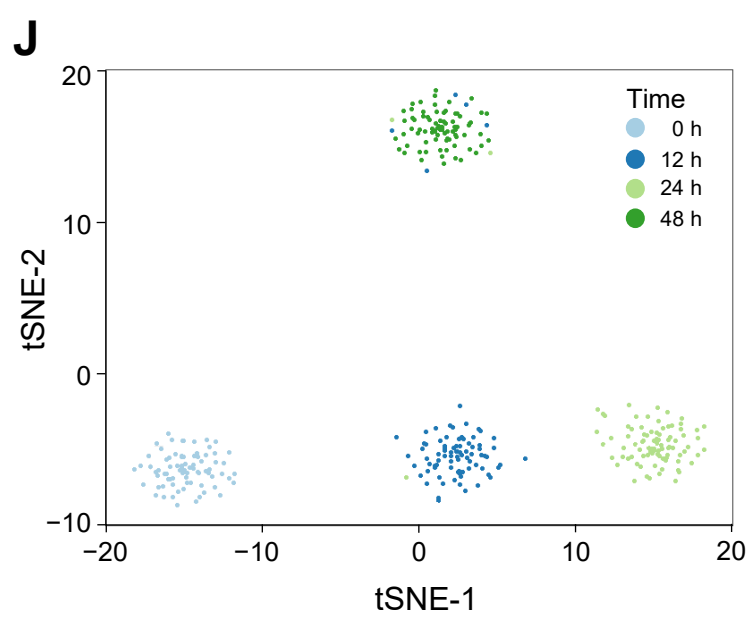

Supplement: Supplementary Figure S5 [file mmc6.pdf]

**A** Network change of MHC-to-HCC

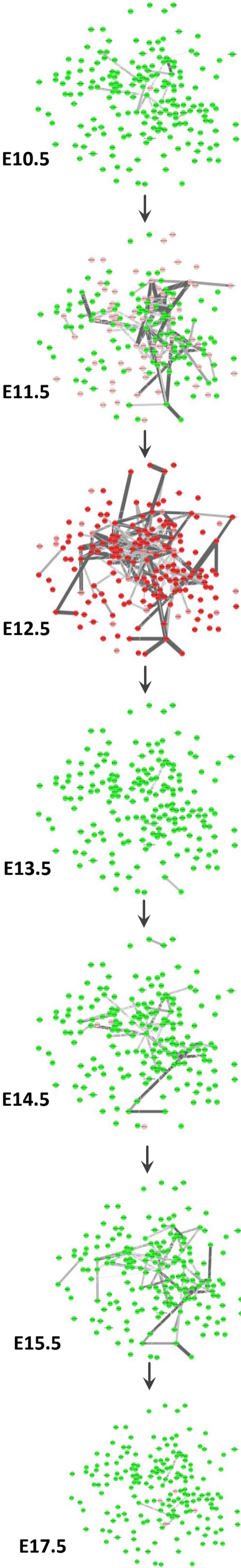

**B** Network change of hESC-to-DEC

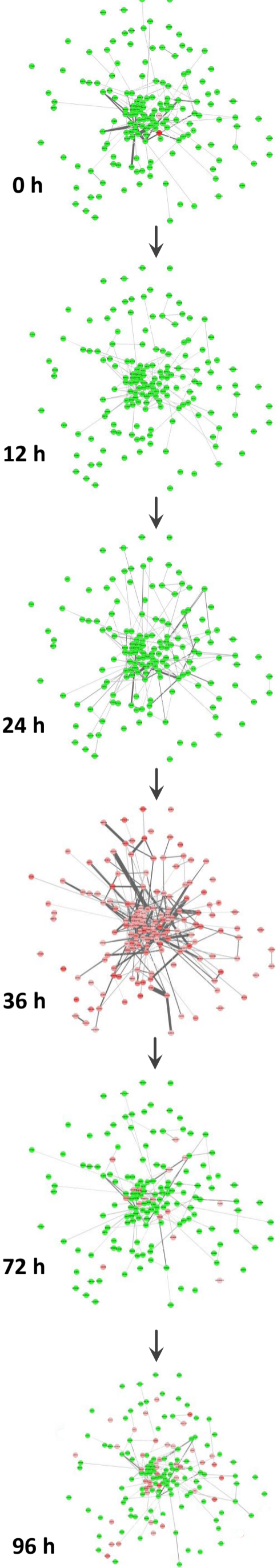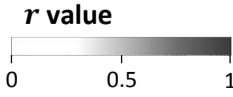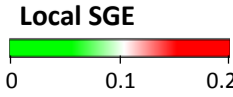

Supplement: Supplementary Figure S7 [file mmc9.pdf]

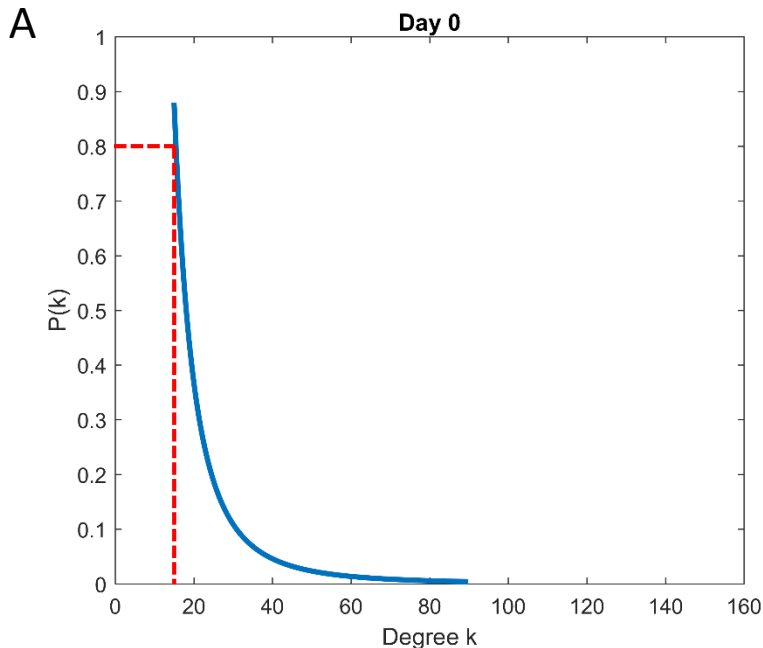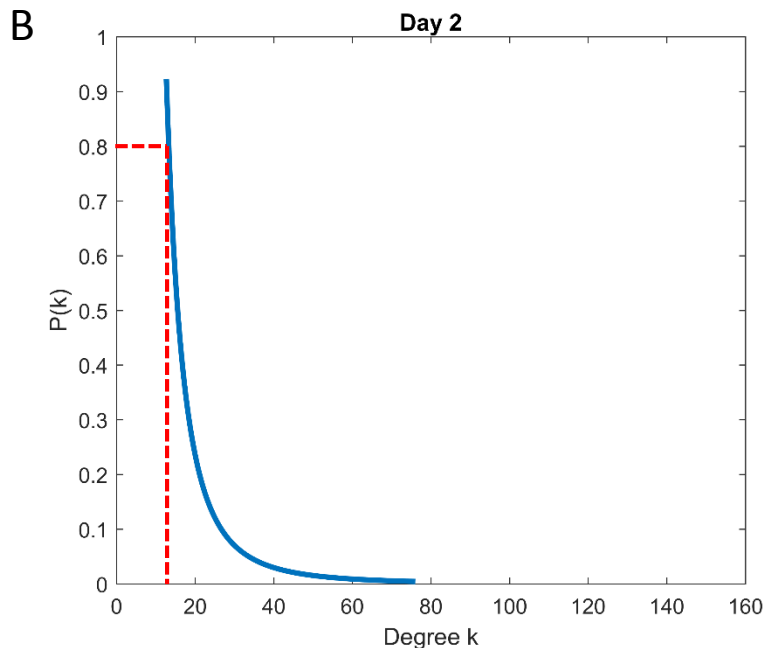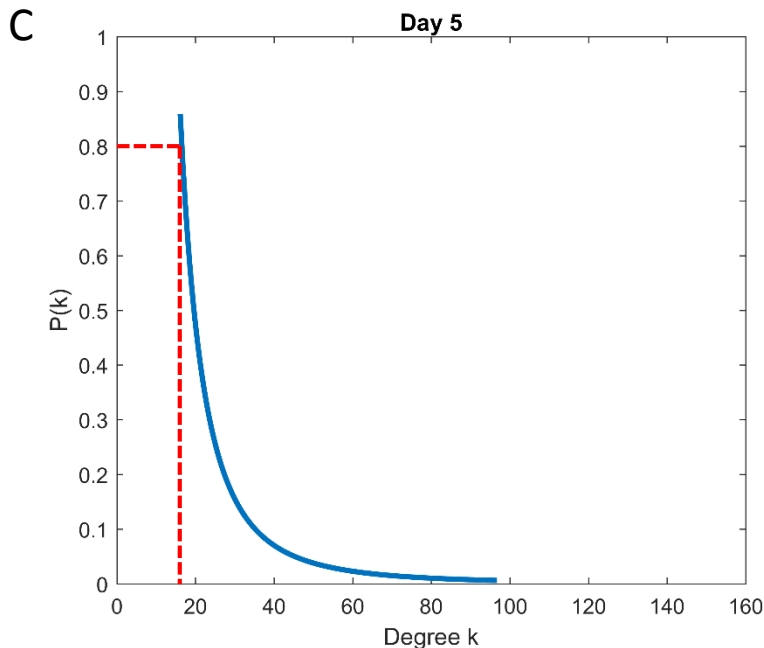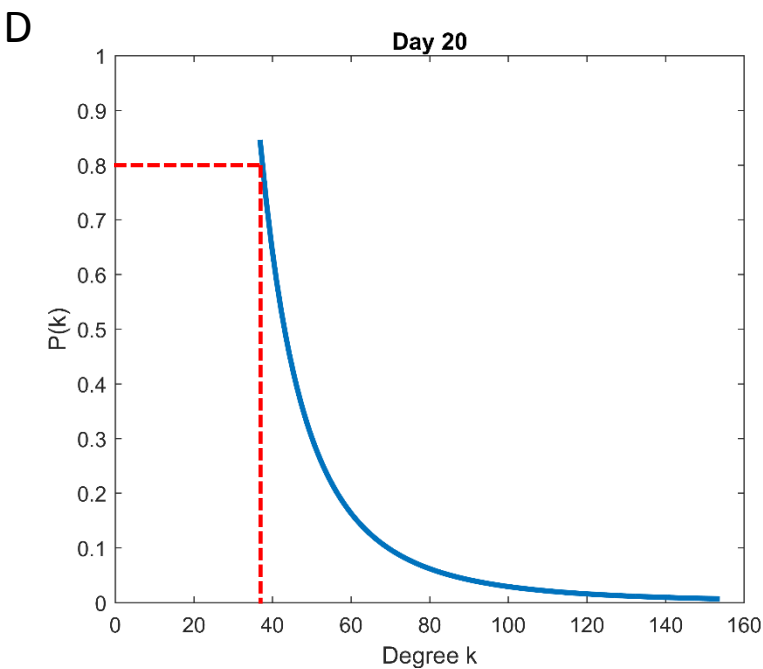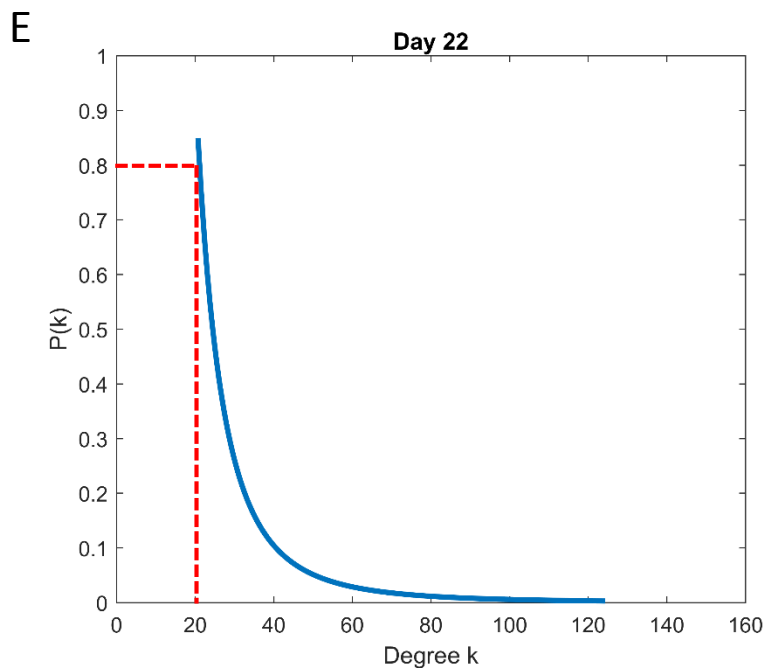

| Time   | Average exponent $\gamma$ |
|--------|---------------------------|
| Day 0  | 3.0041                    |
| Day 2  | 2.9858                    |
| Day 5  | 2.7525                    |
| Day 20 | 3.3735                    |
| Day 22 | 3.1879                    |

Supplement: Supplementary Figure S8 [file mmc10.pdf]

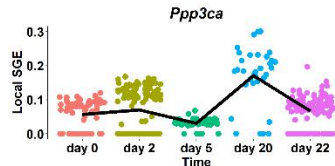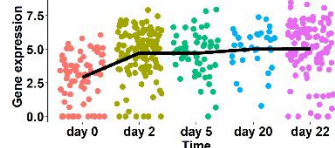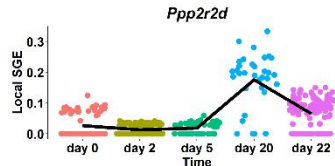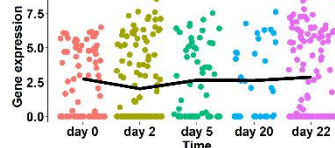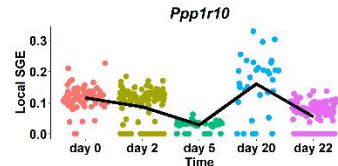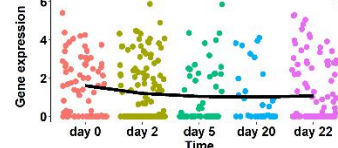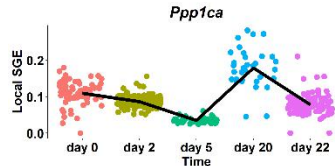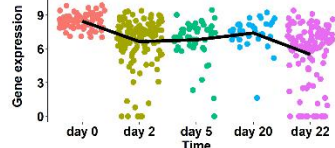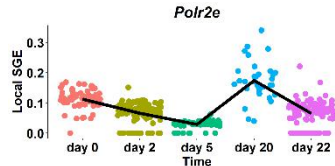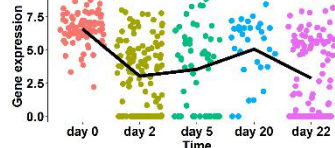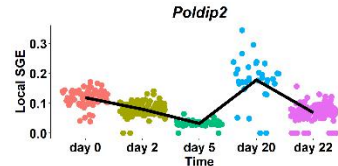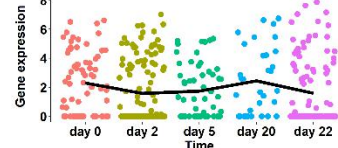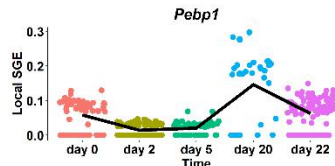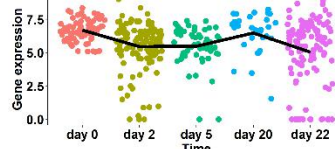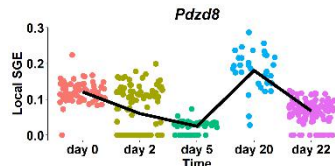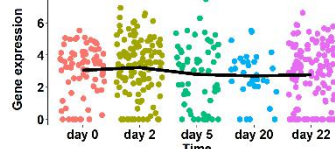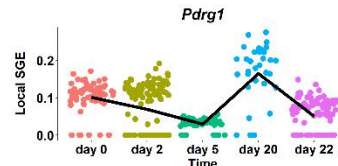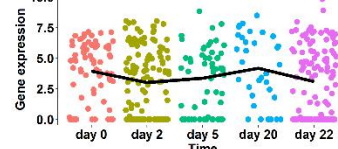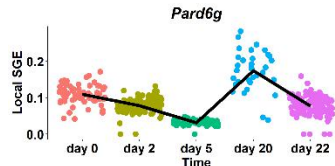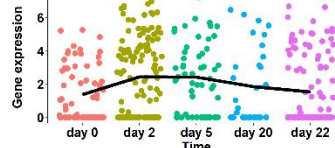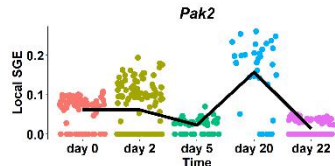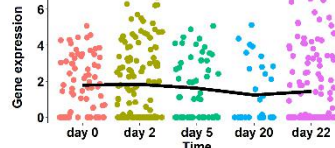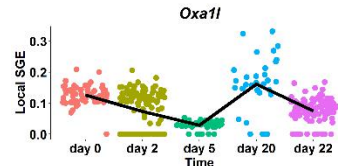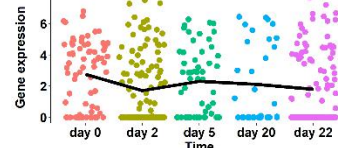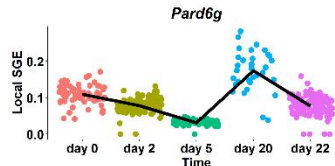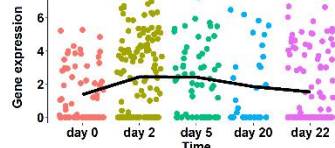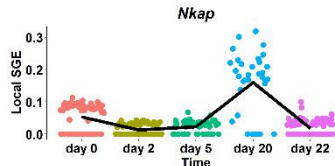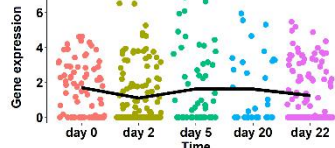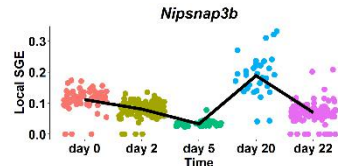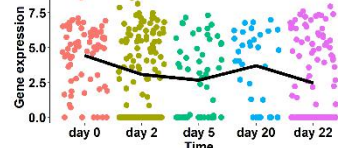

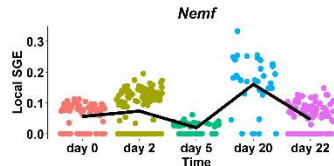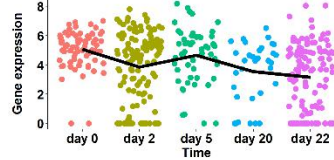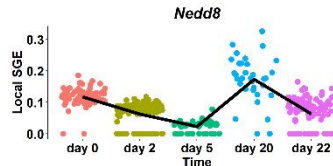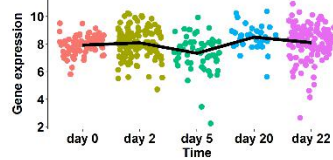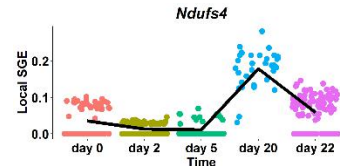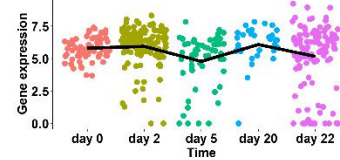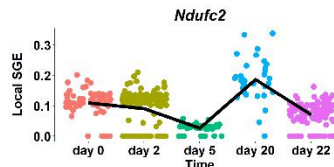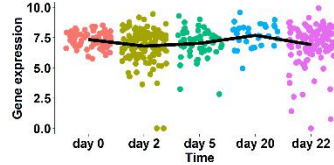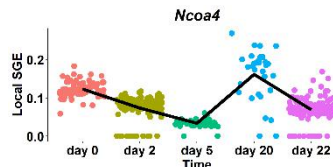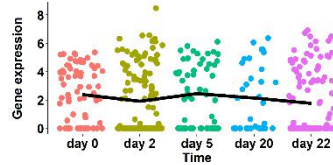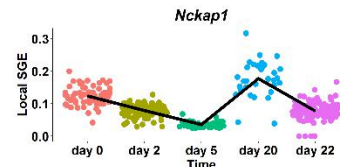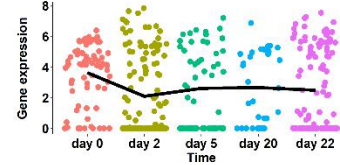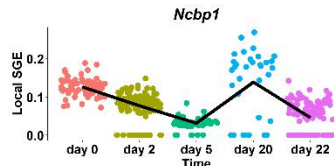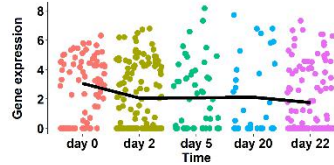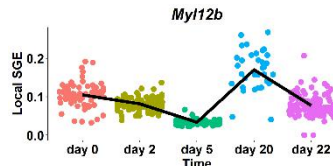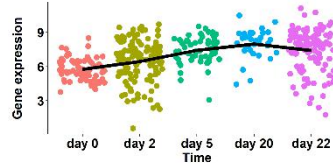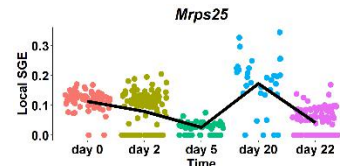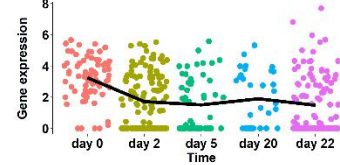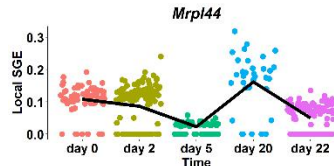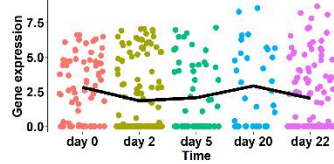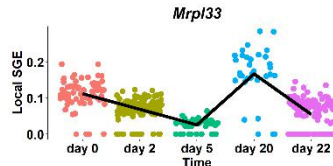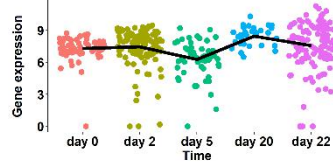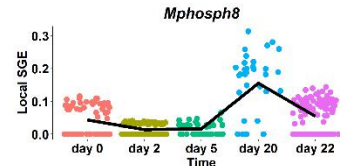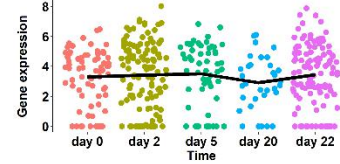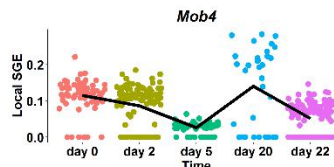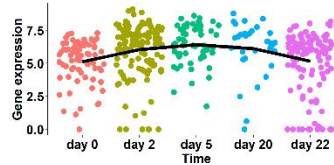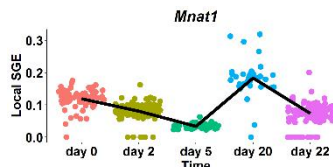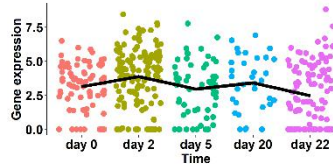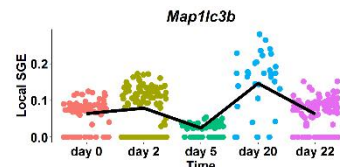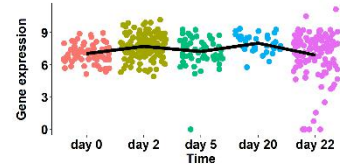

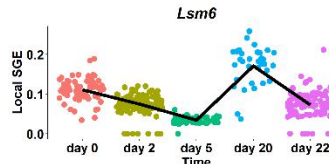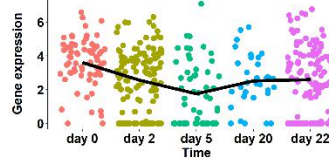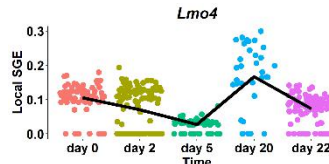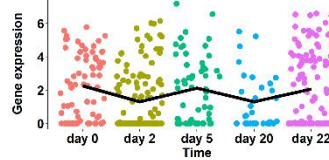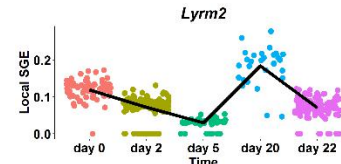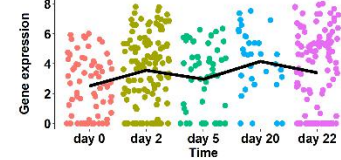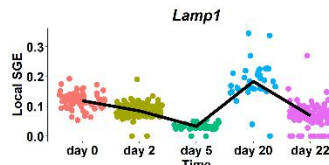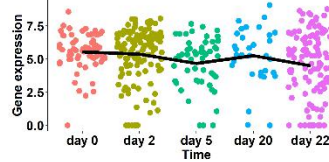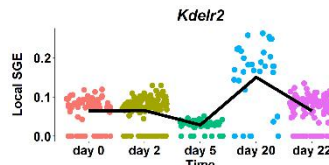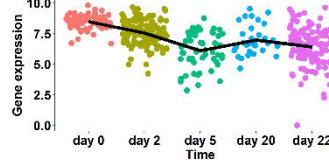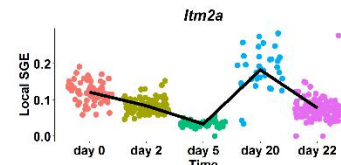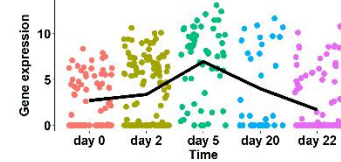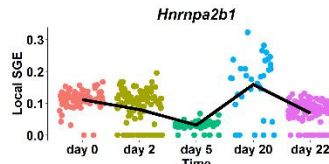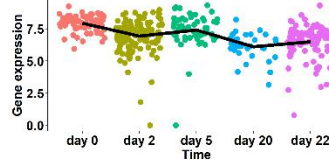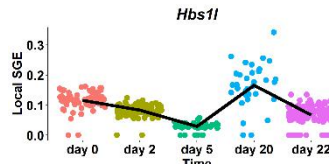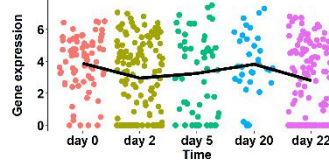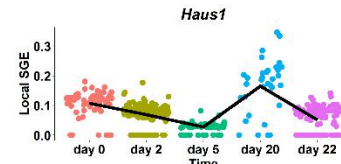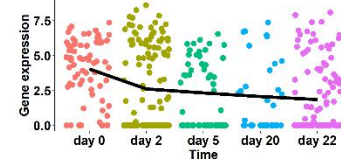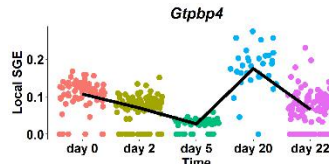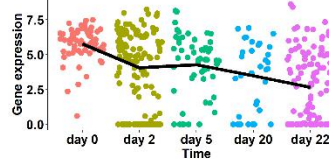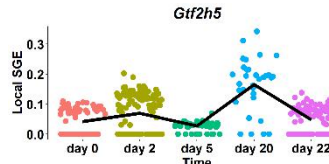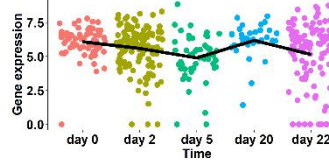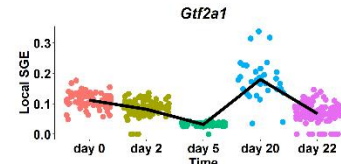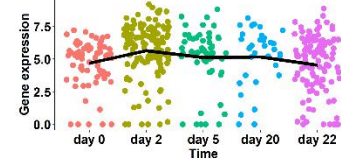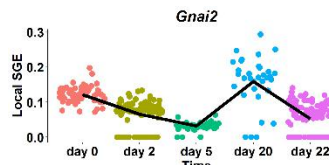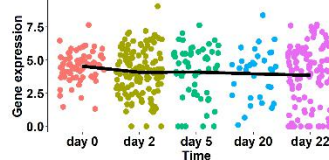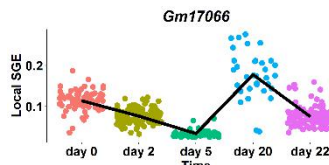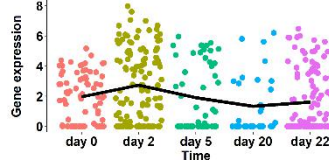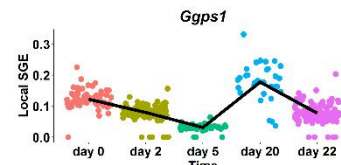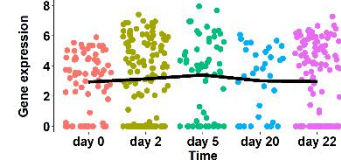

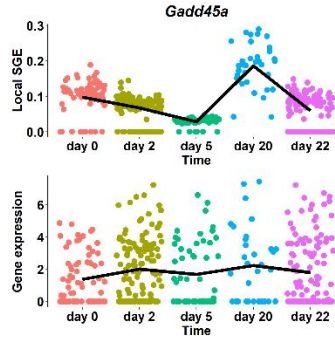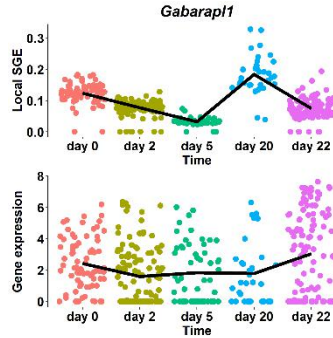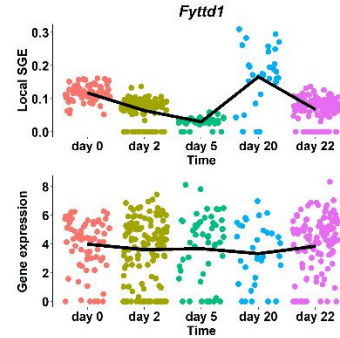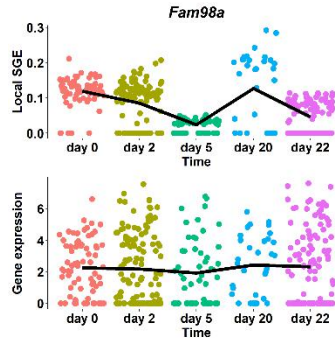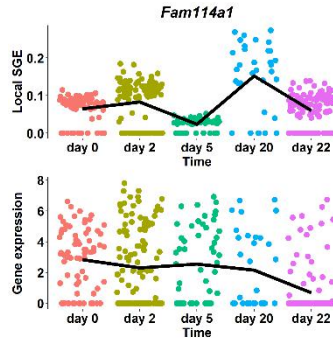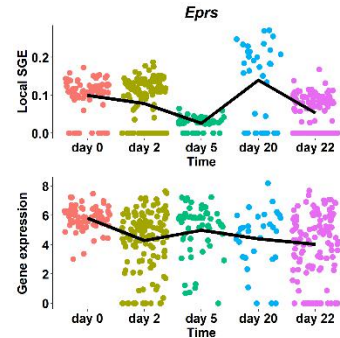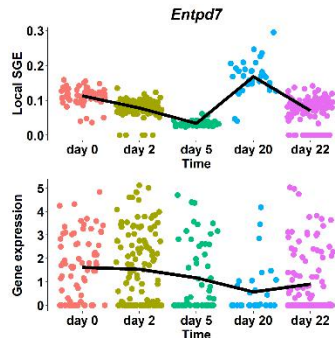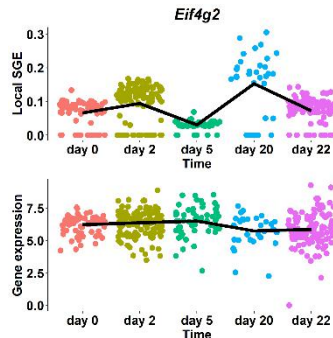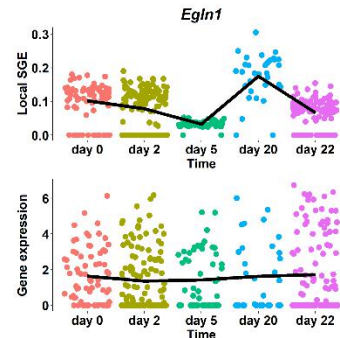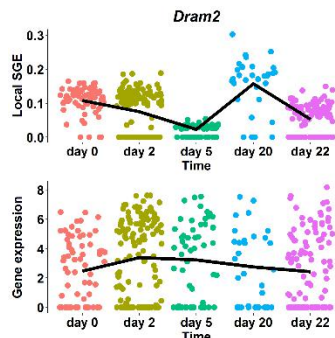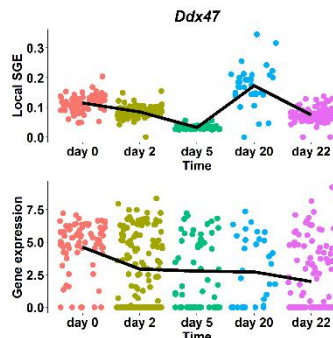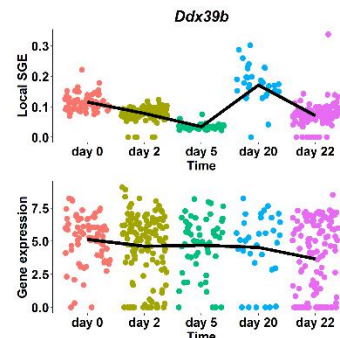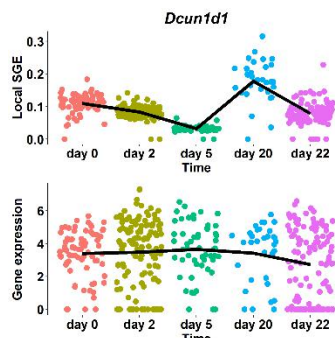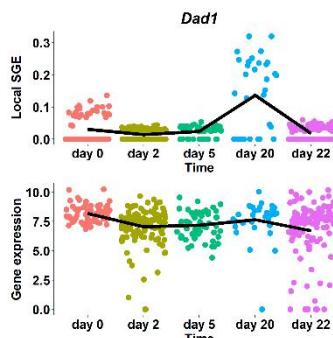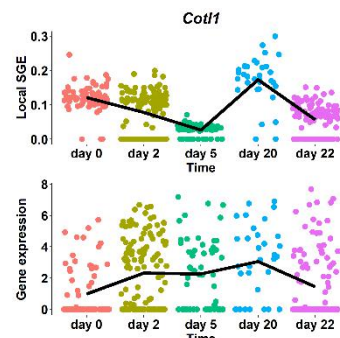

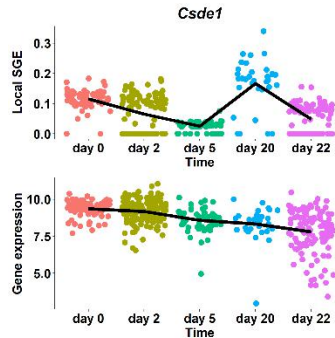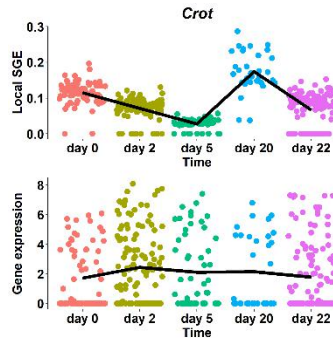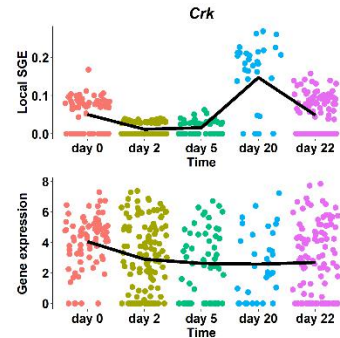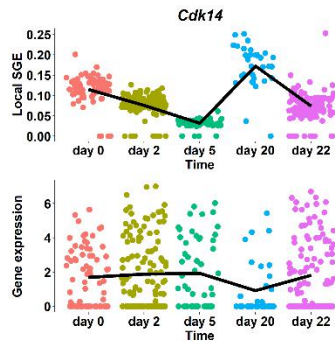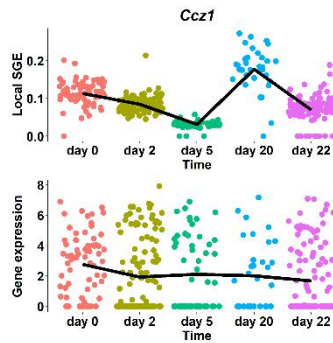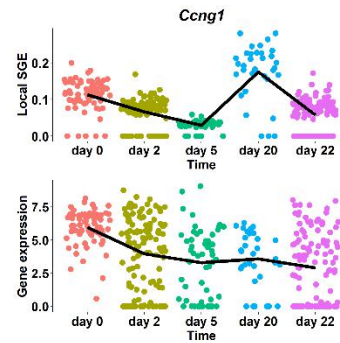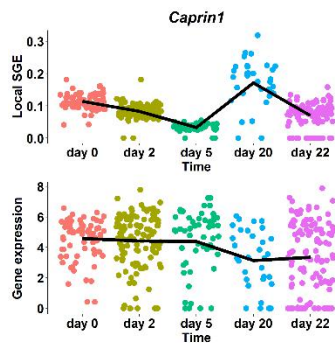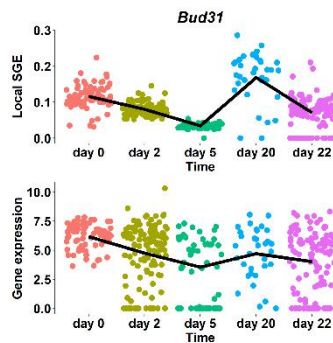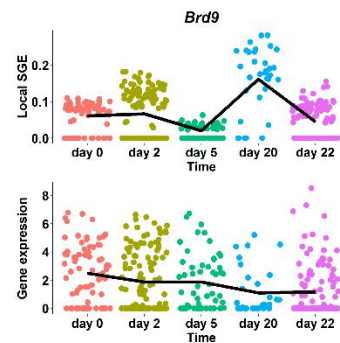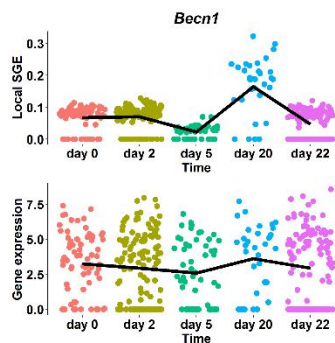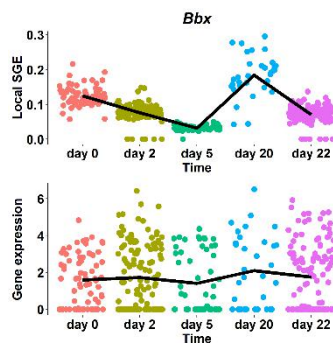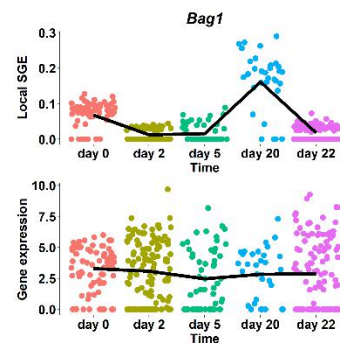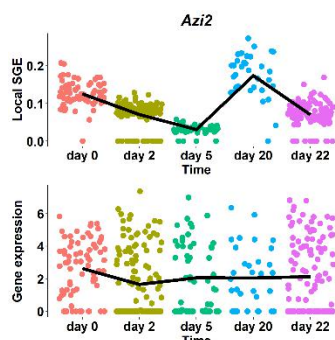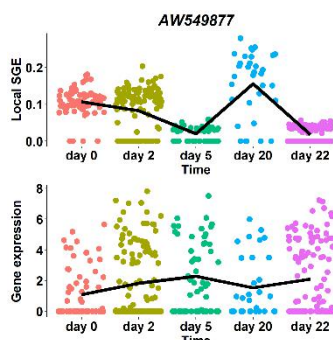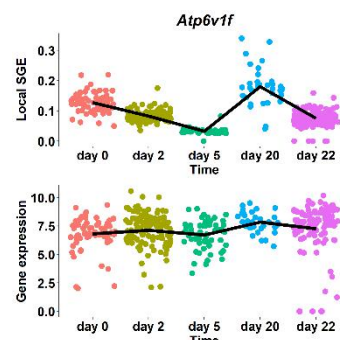

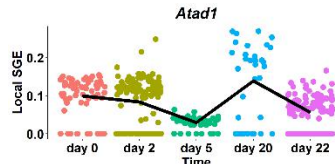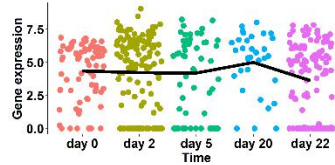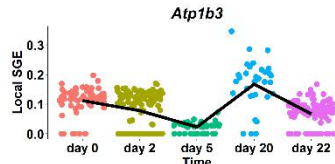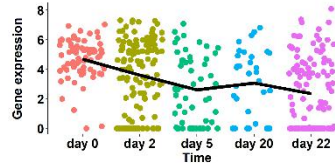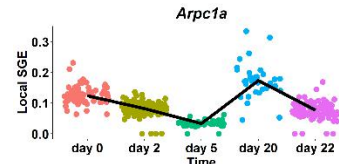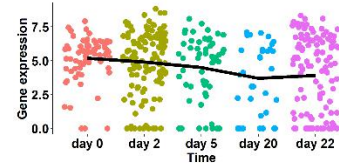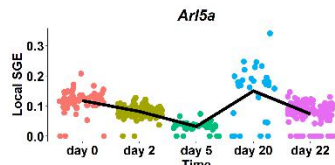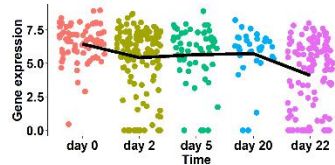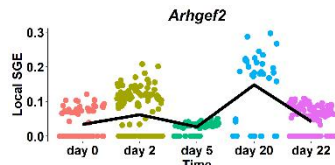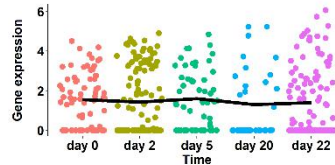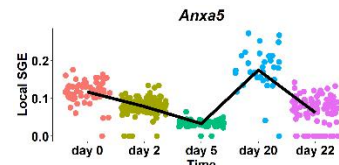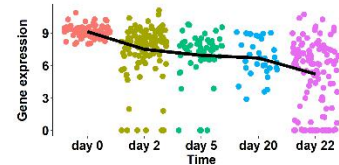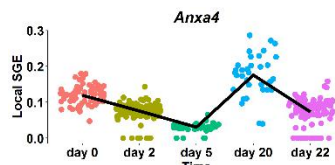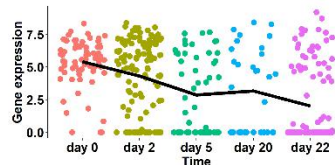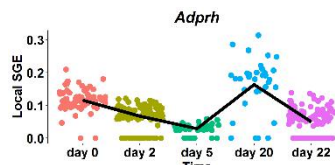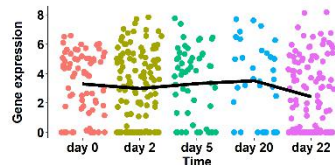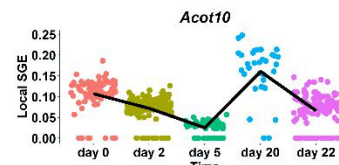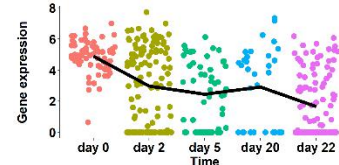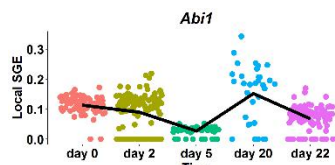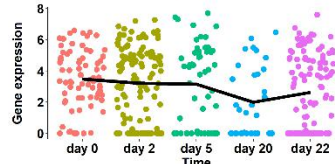

Supplement: Supplementary Figure S9 [file mmc11.pdf]

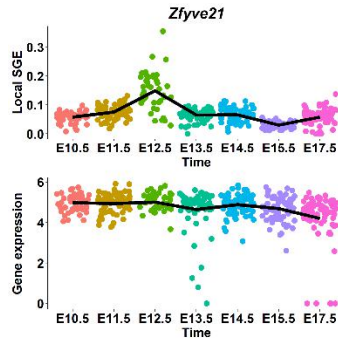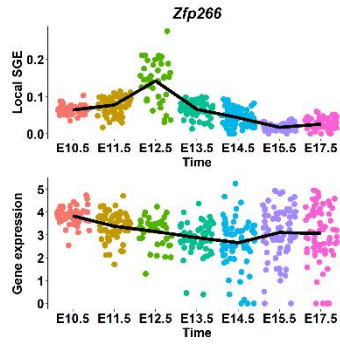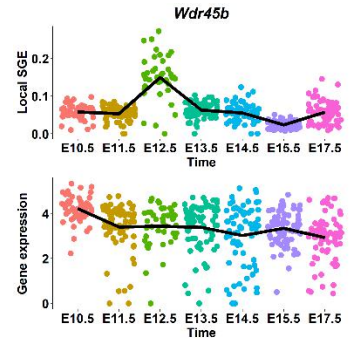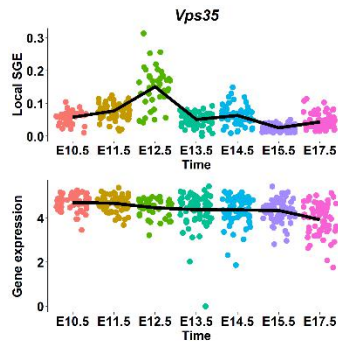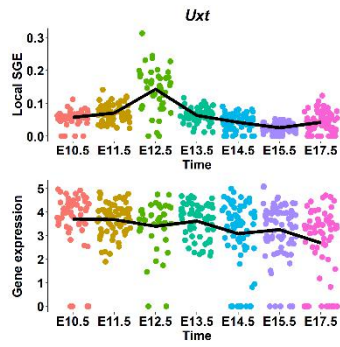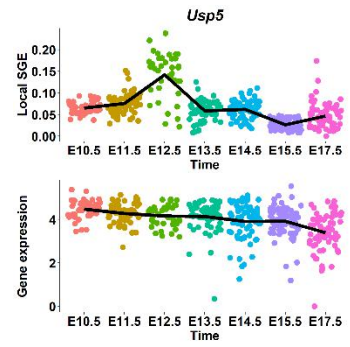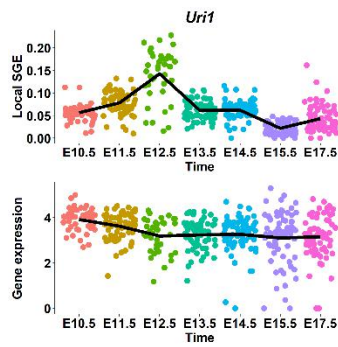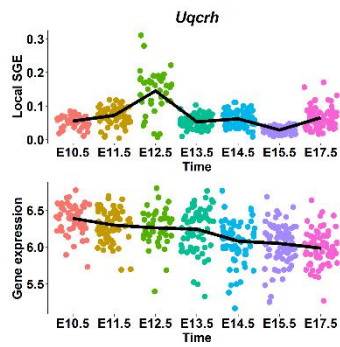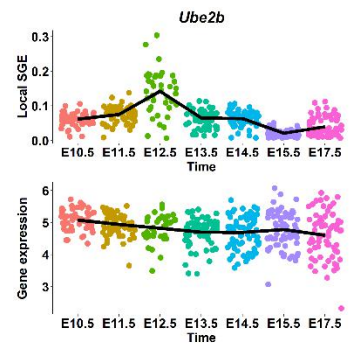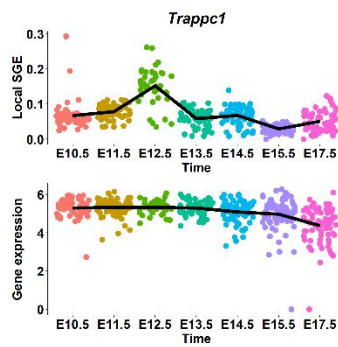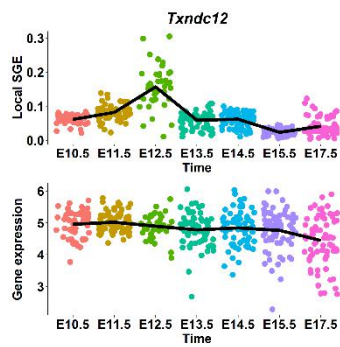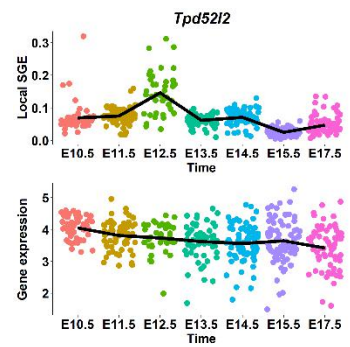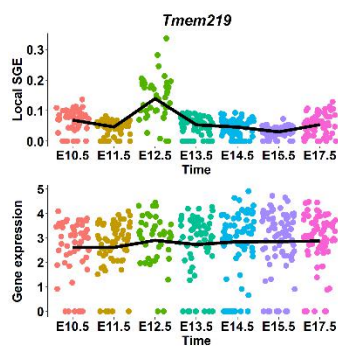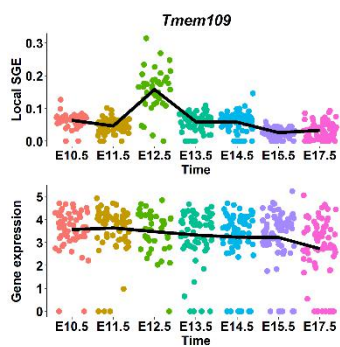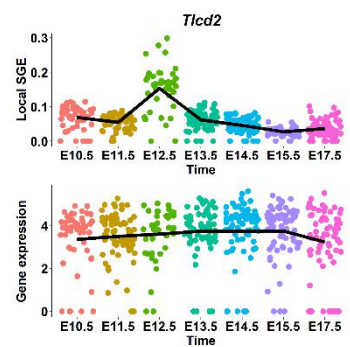

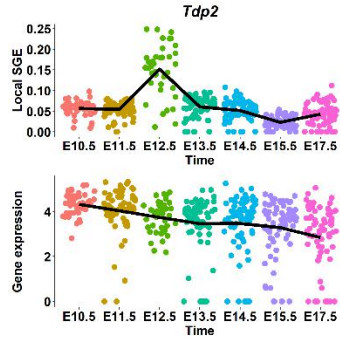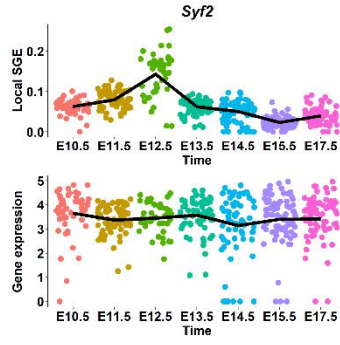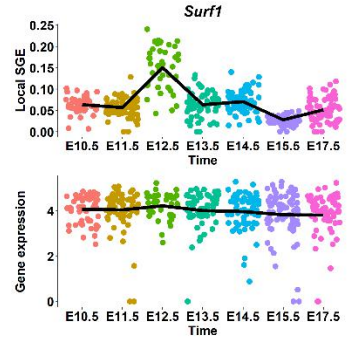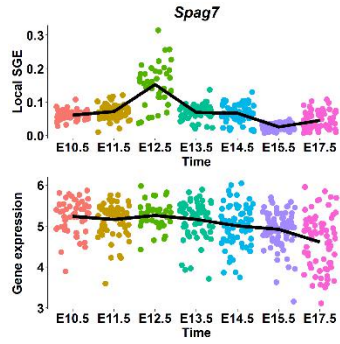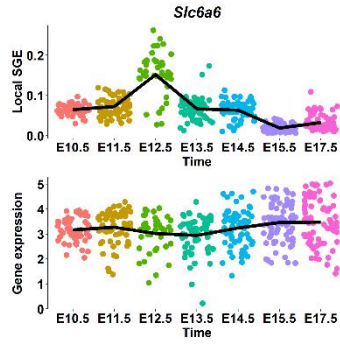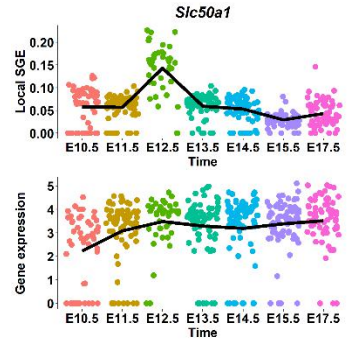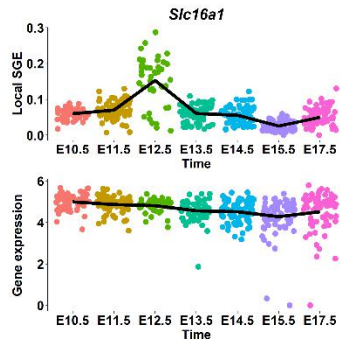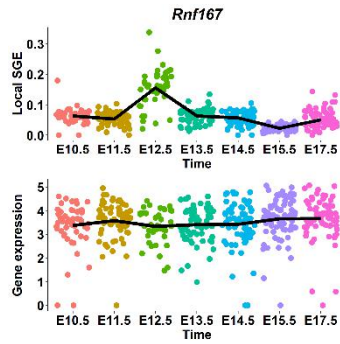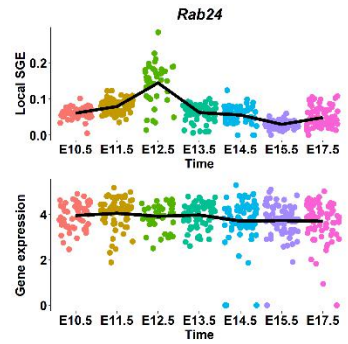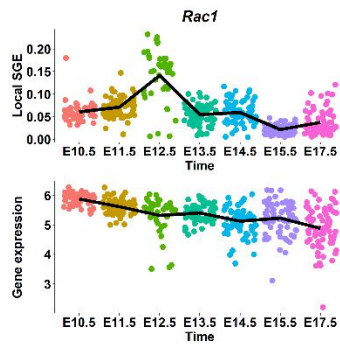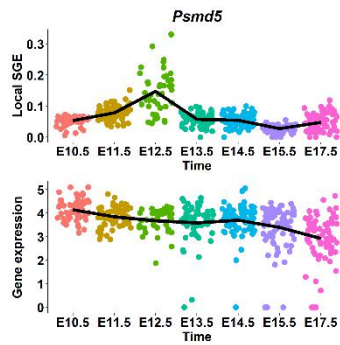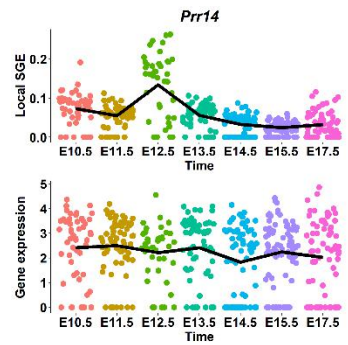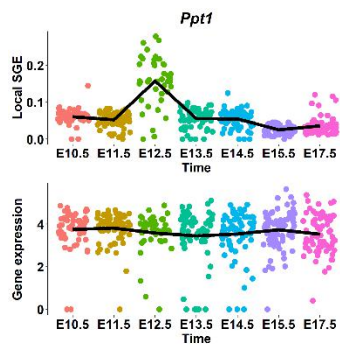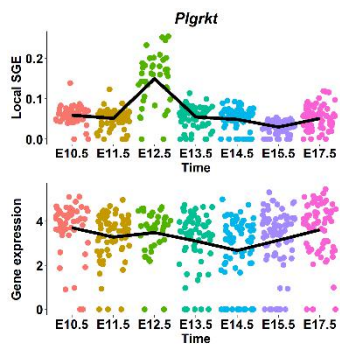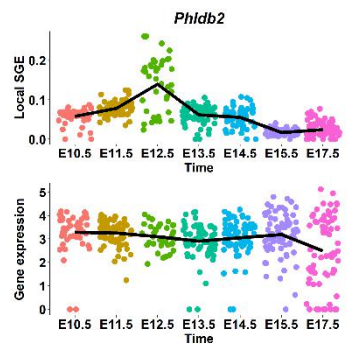

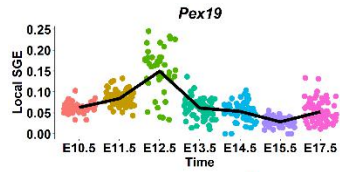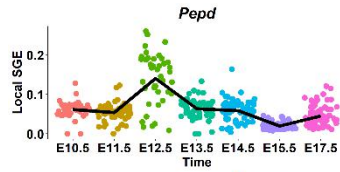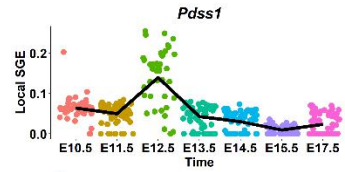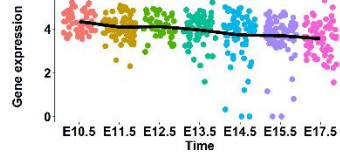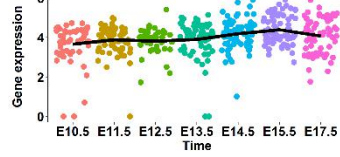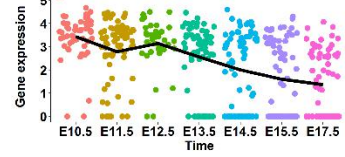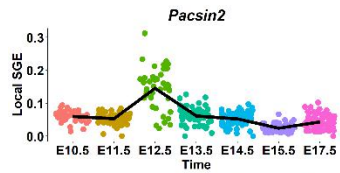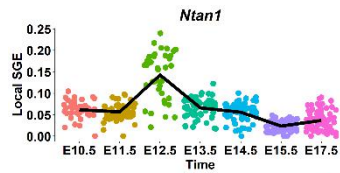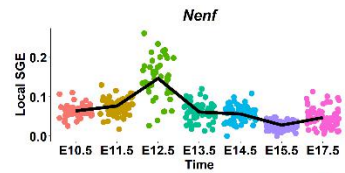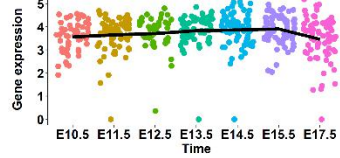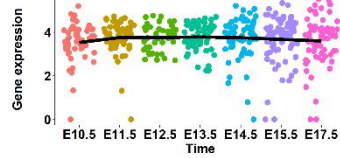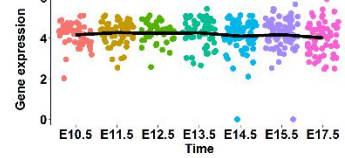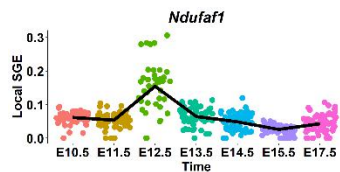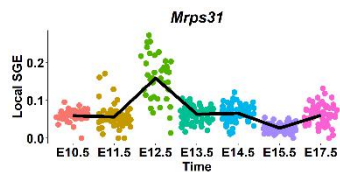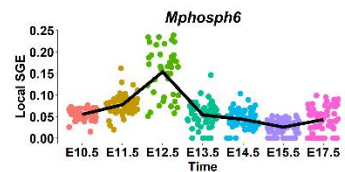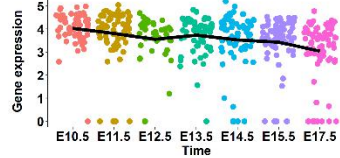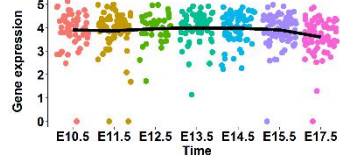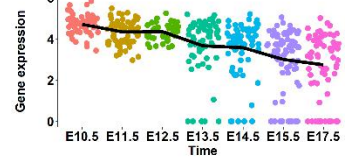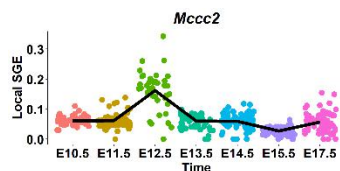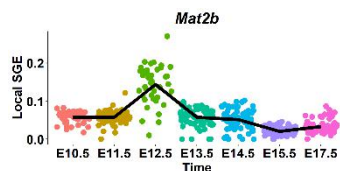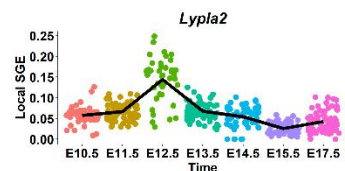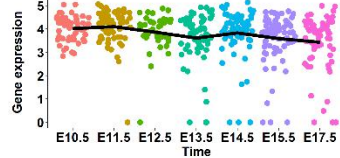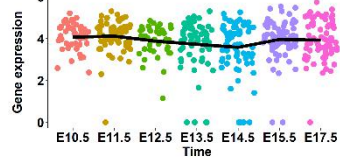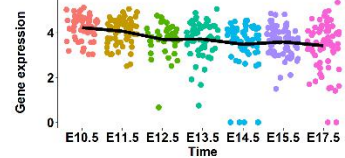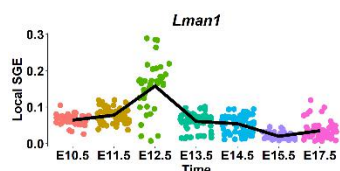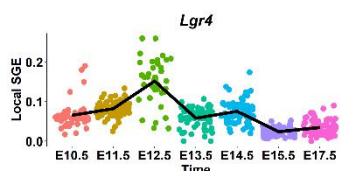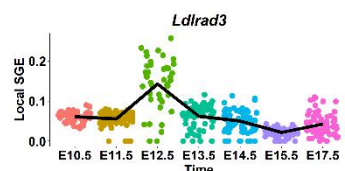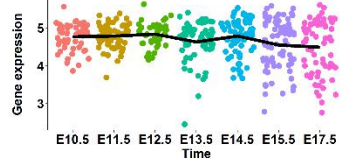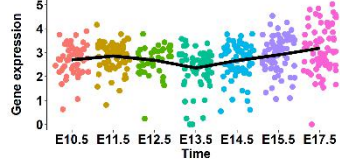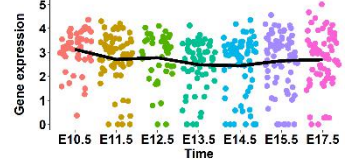

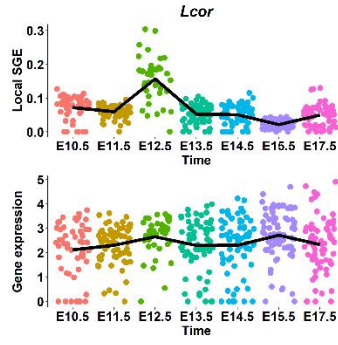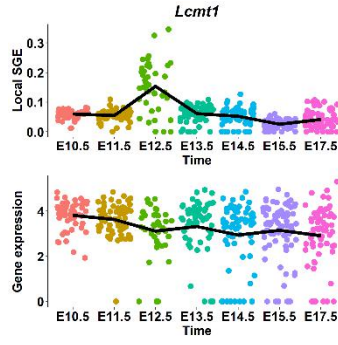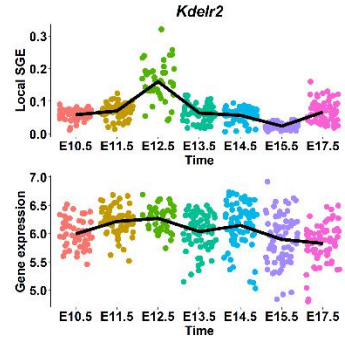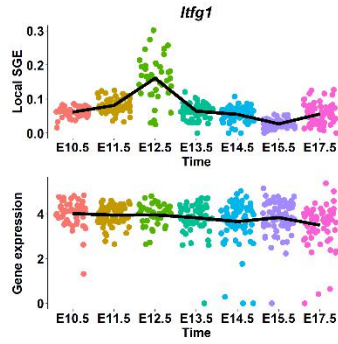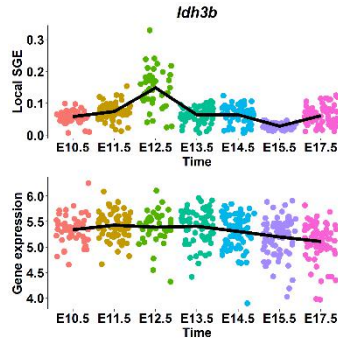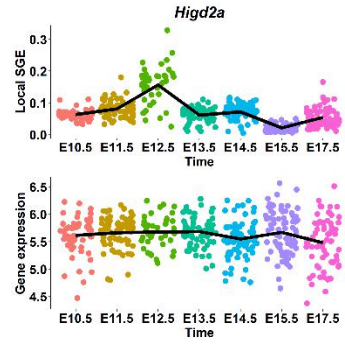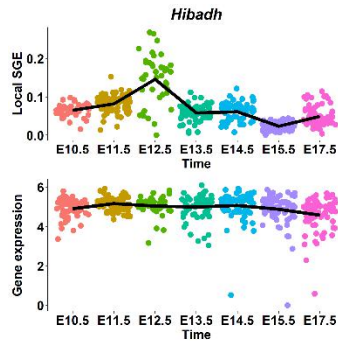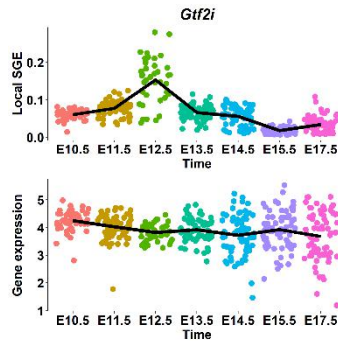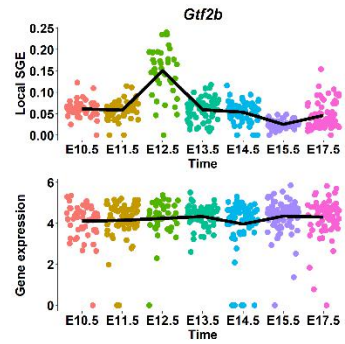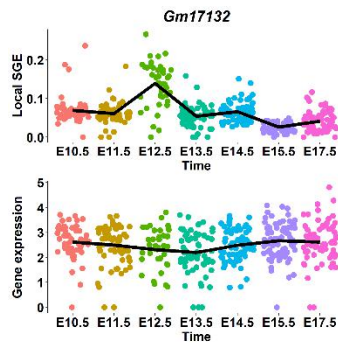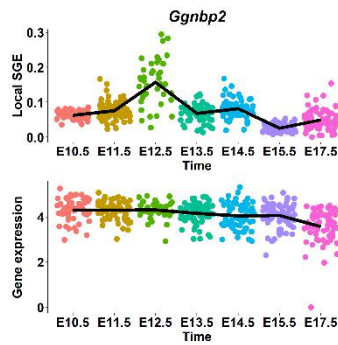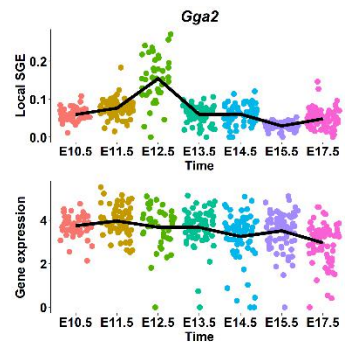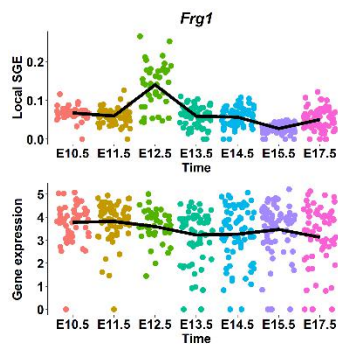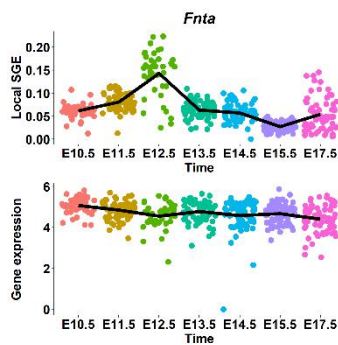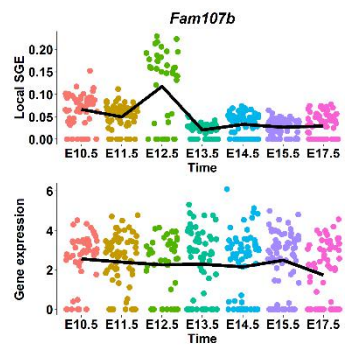

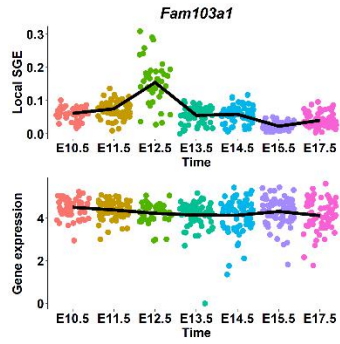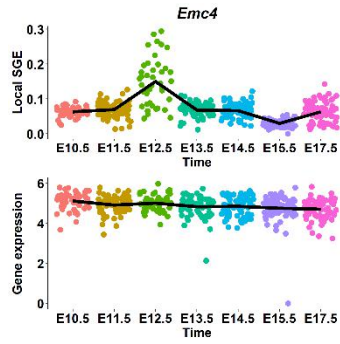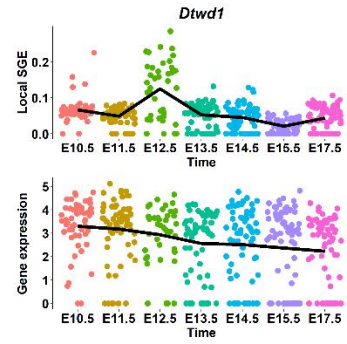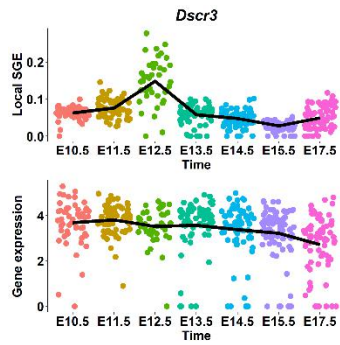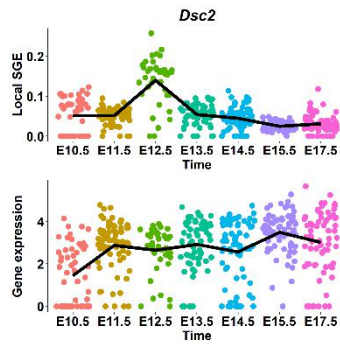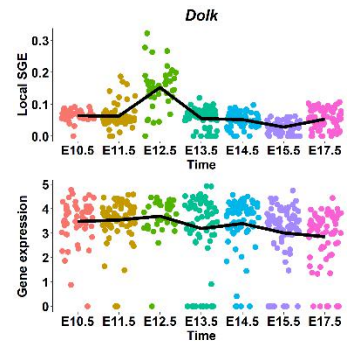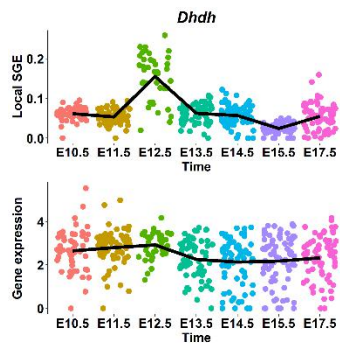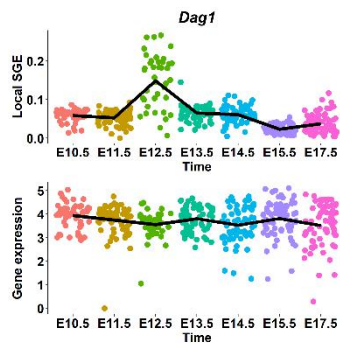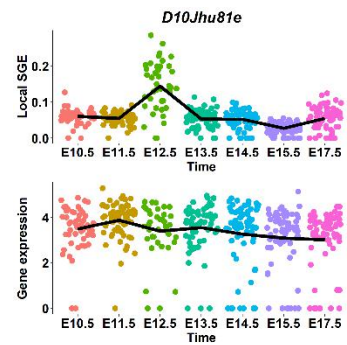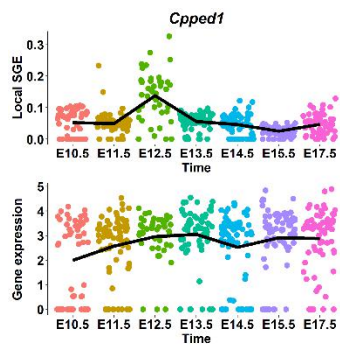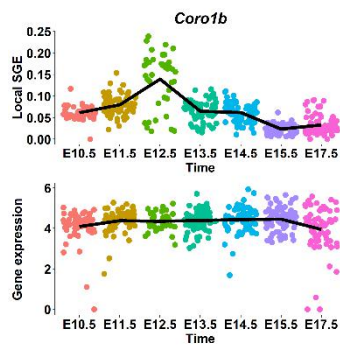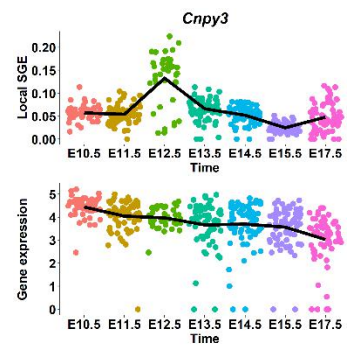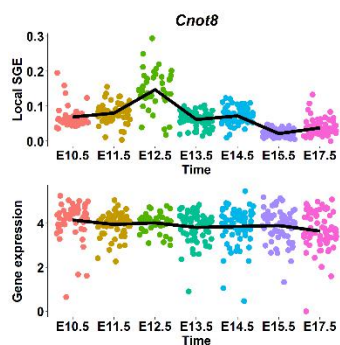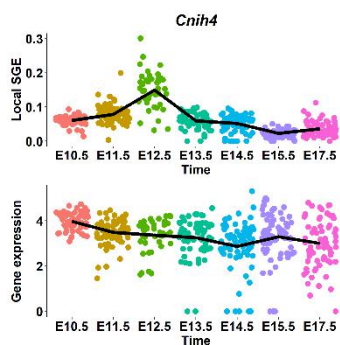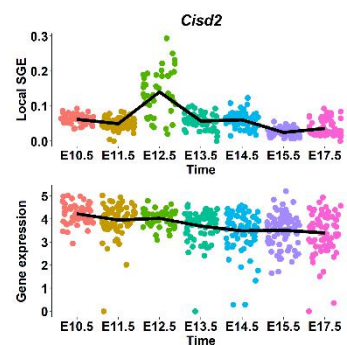

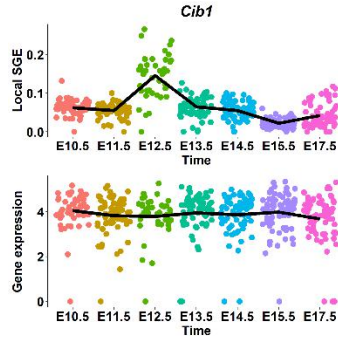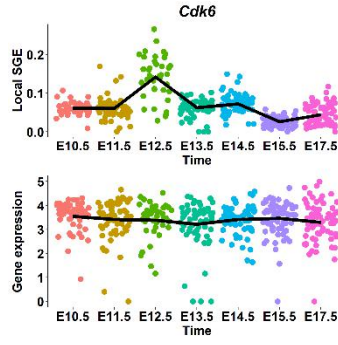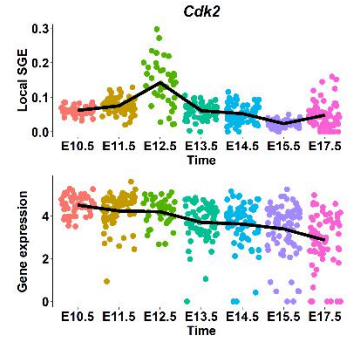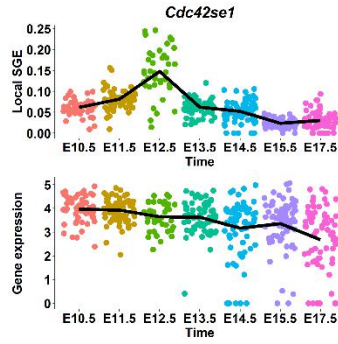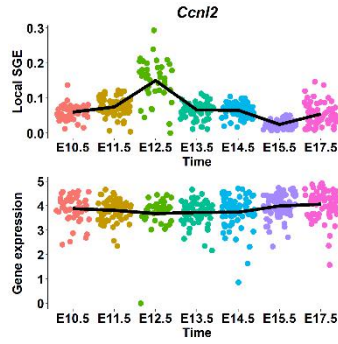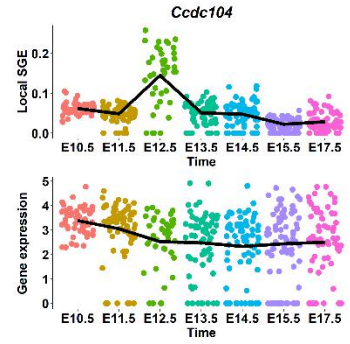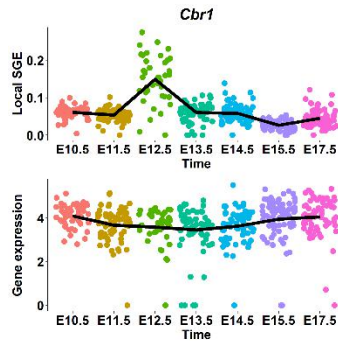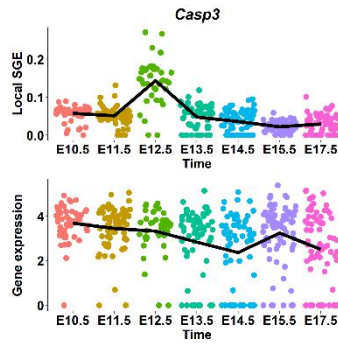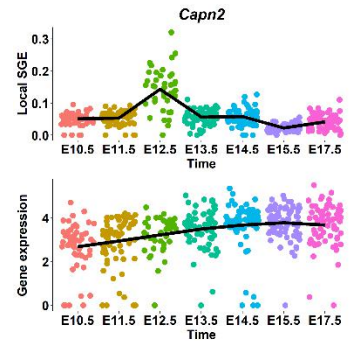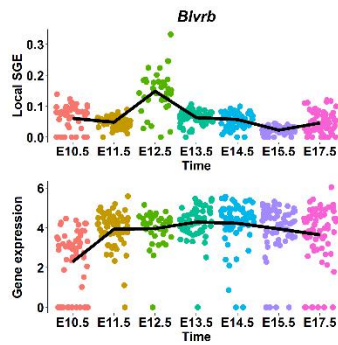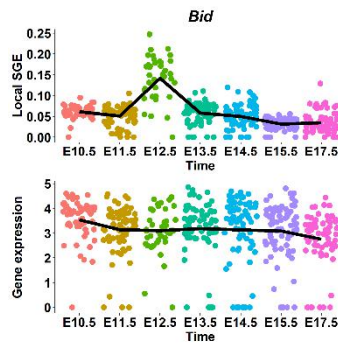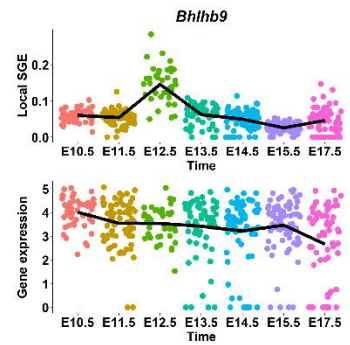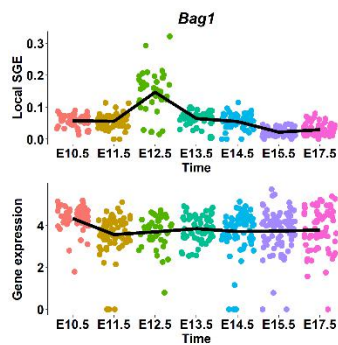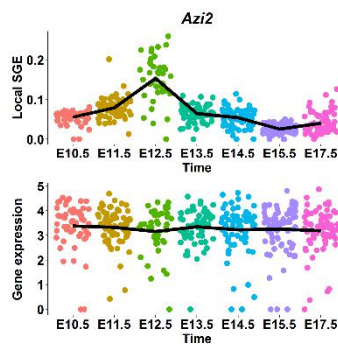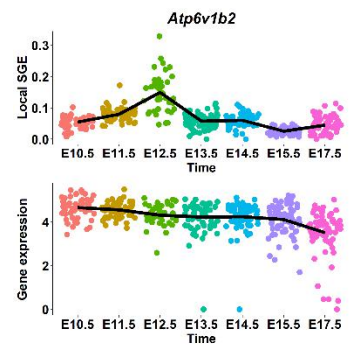

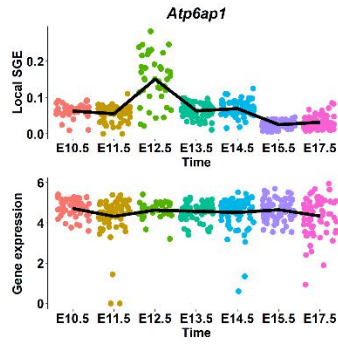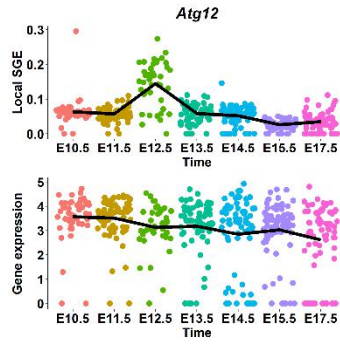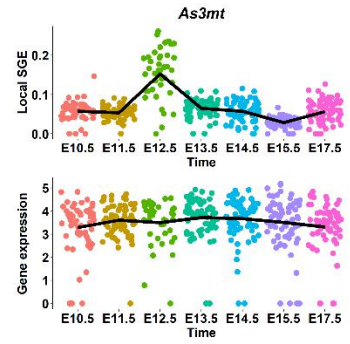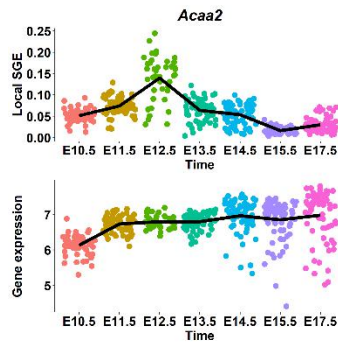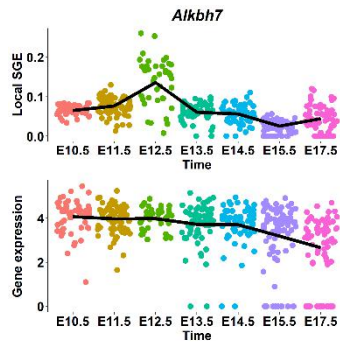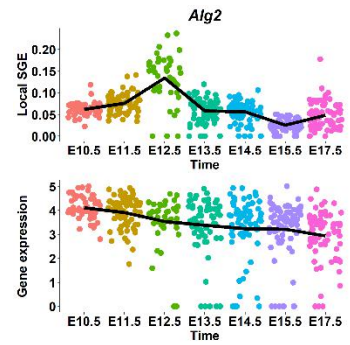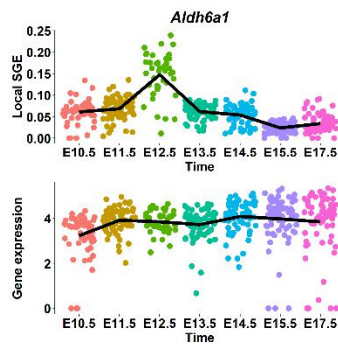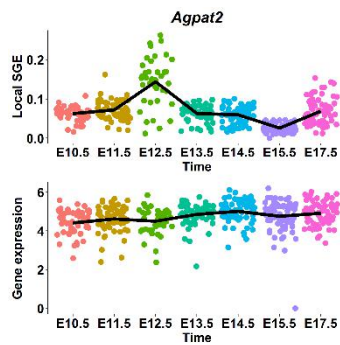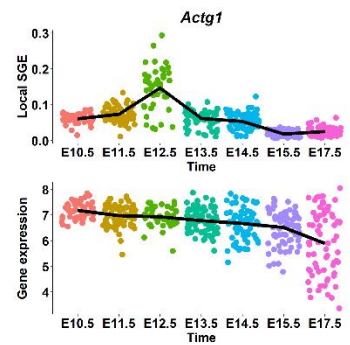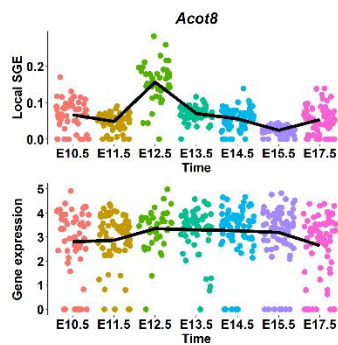

Supplement: Supplementary Figure S10 [file mmc12.pdf]

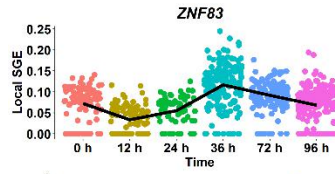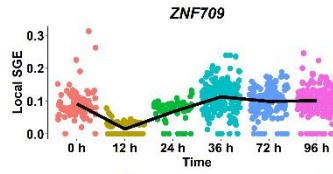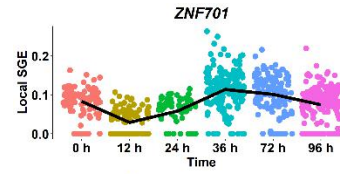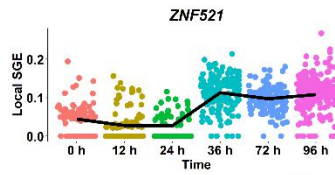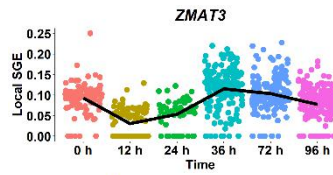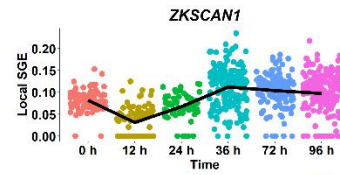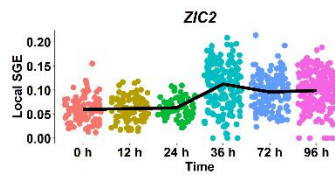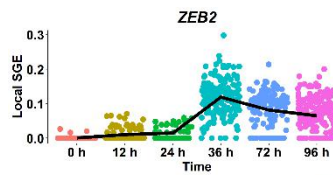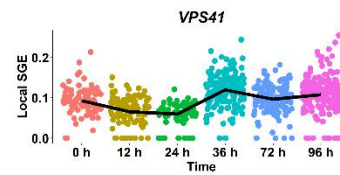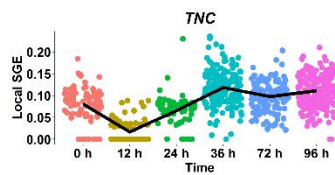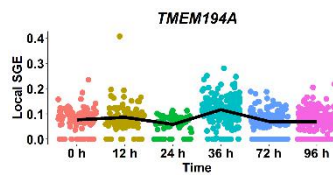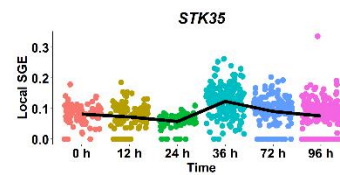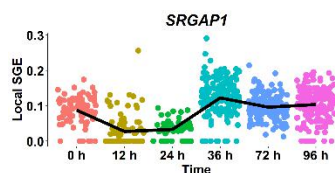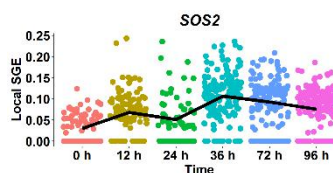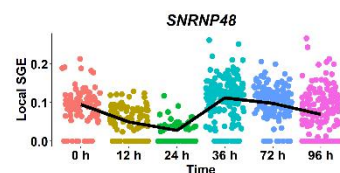

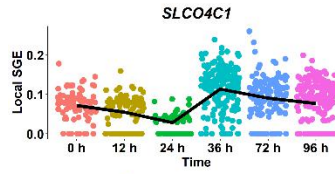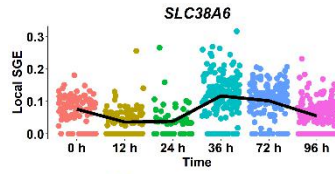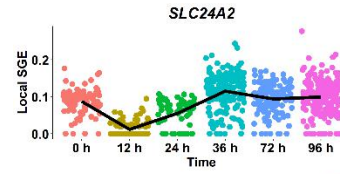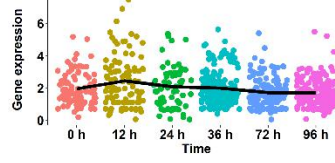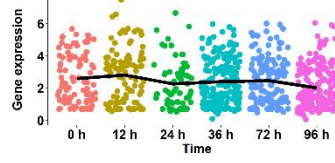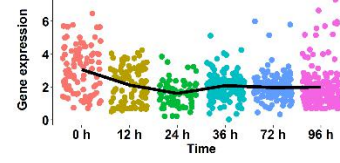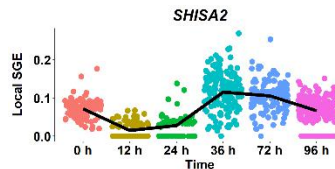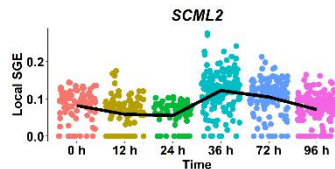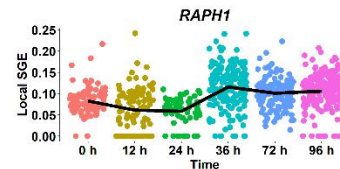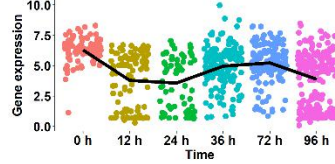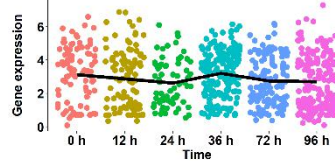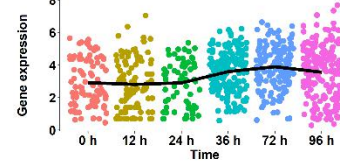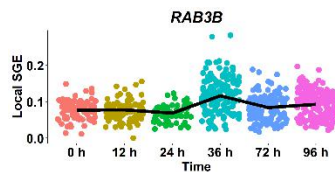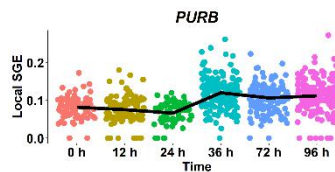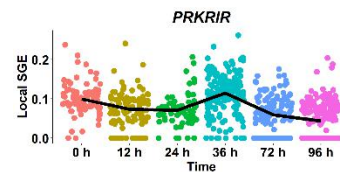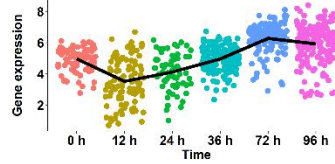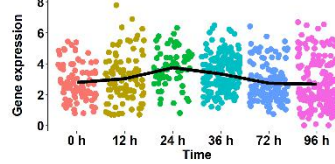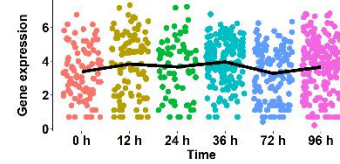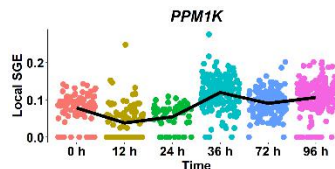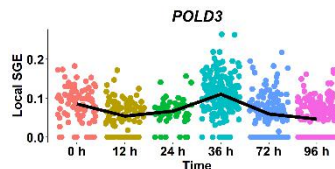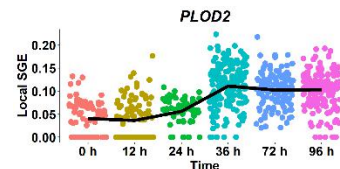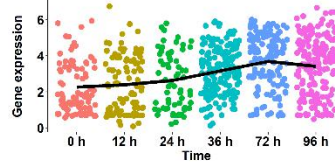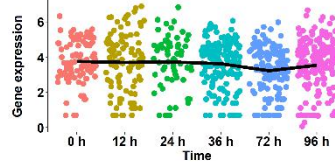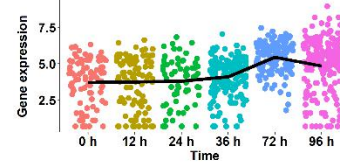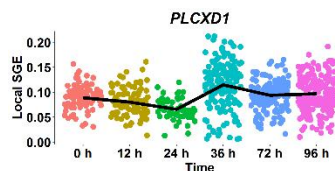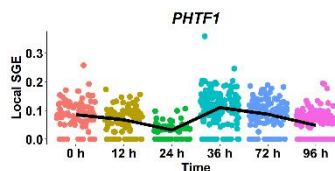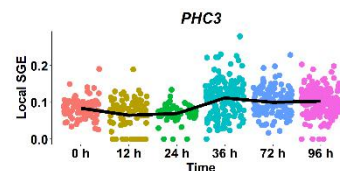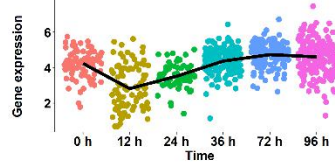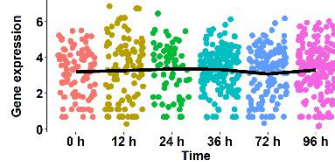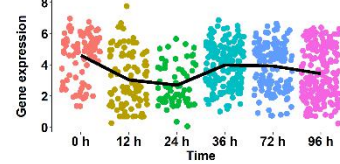

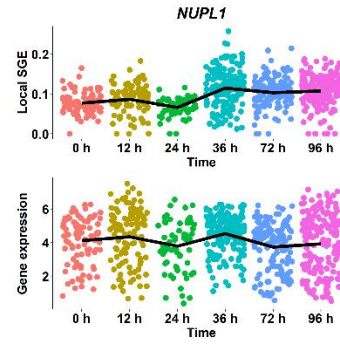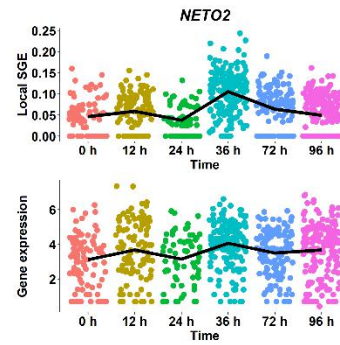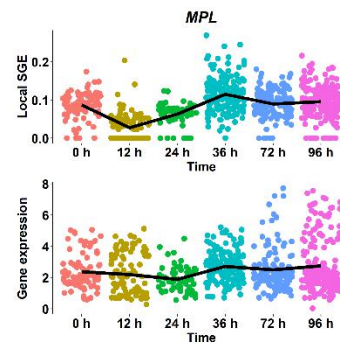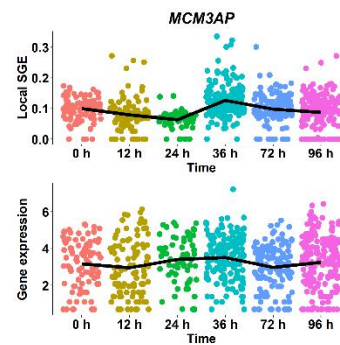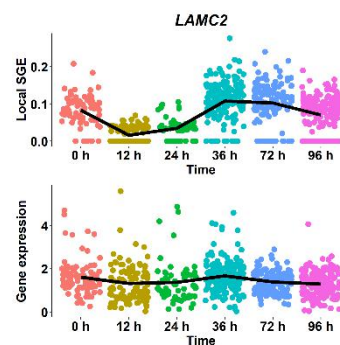

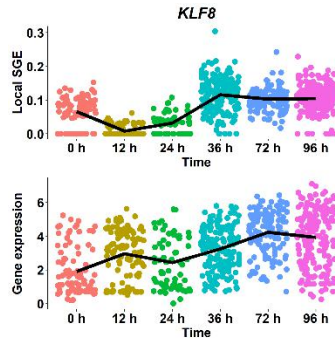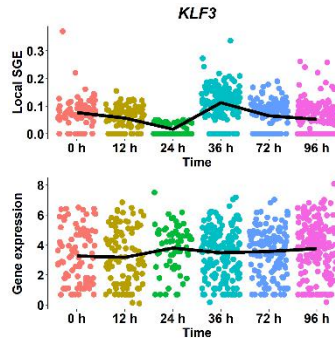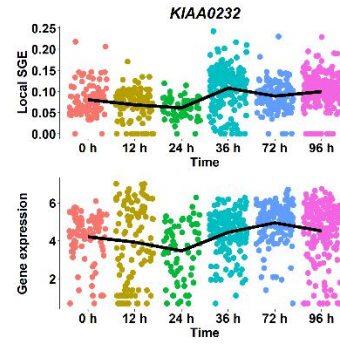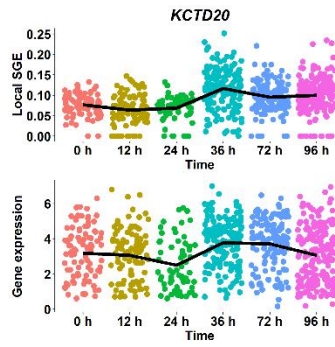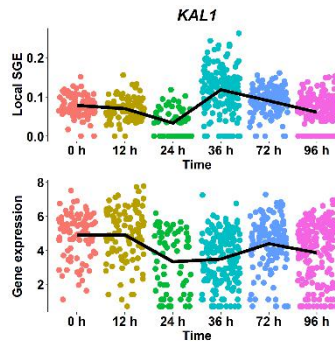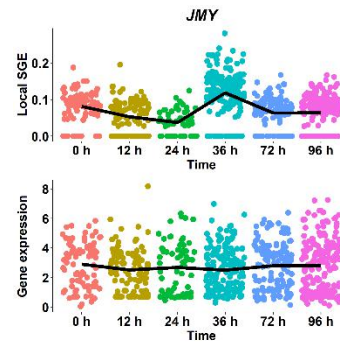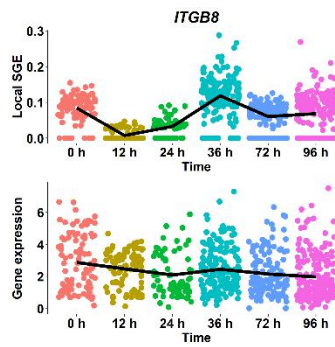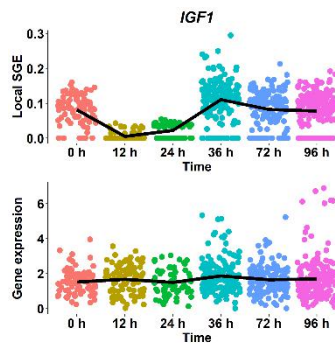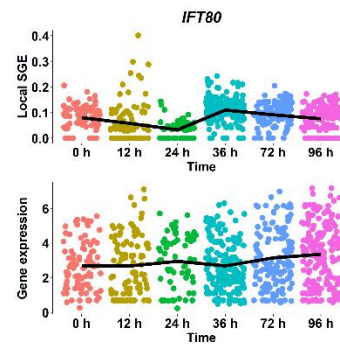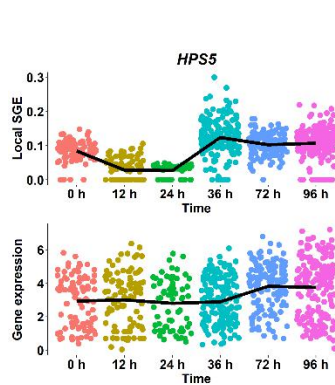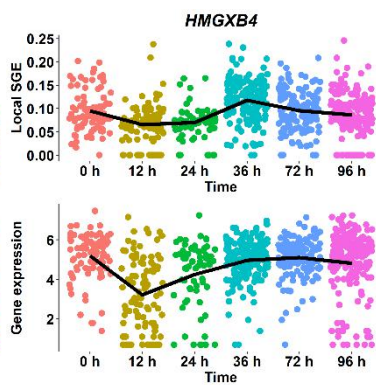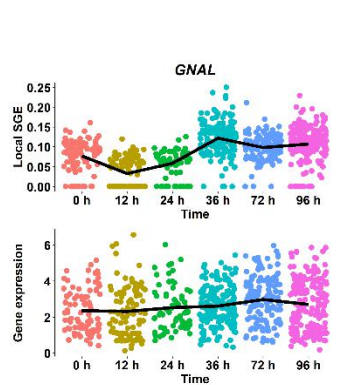

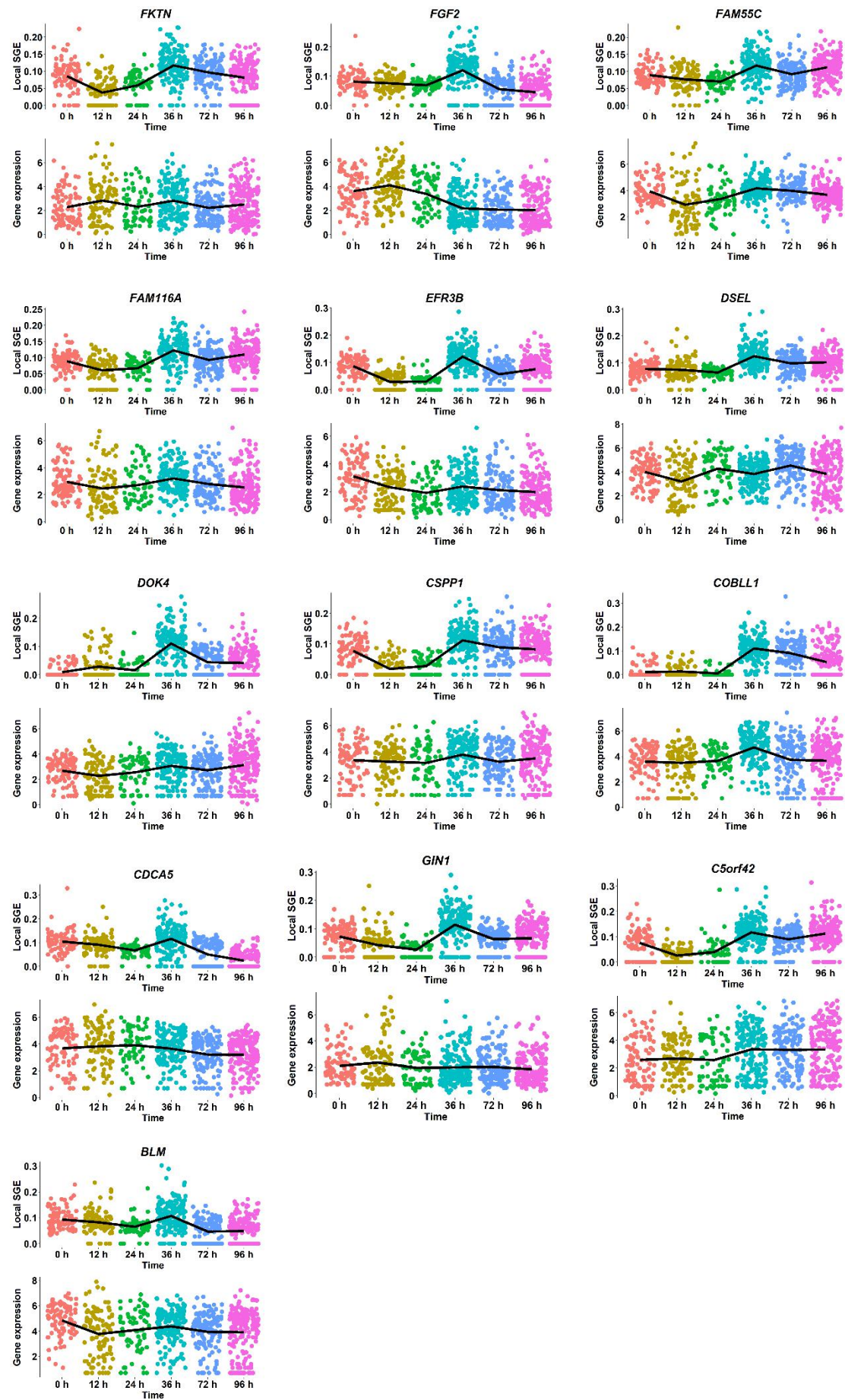

Supplement: Supplementary Figure S11 [file mmc13.pdf]

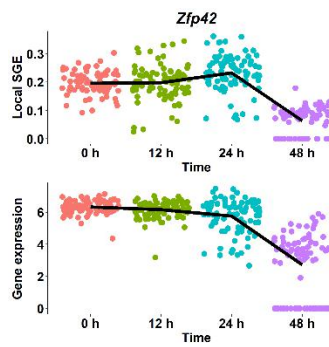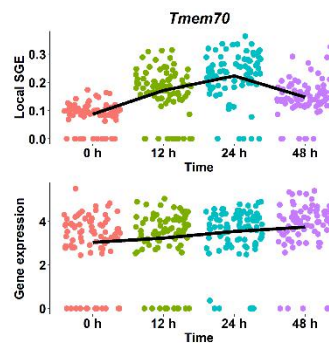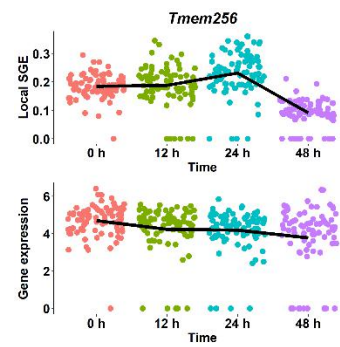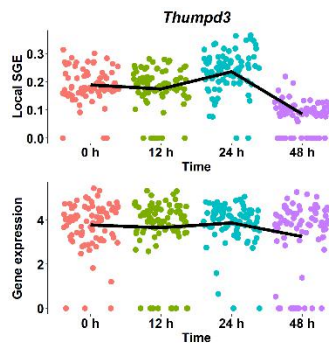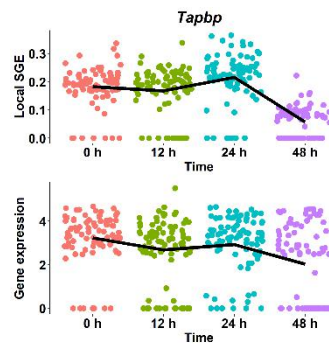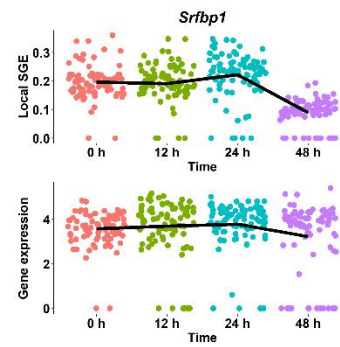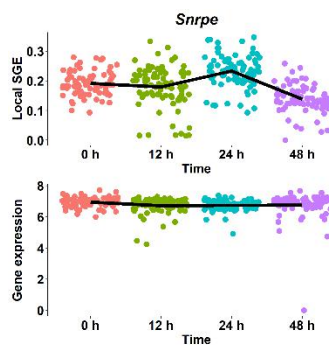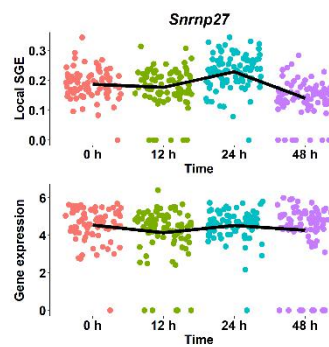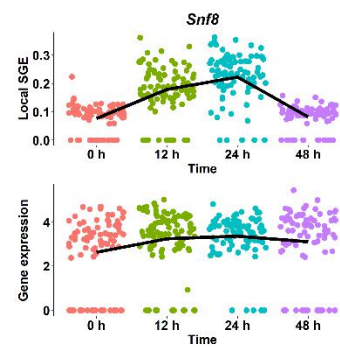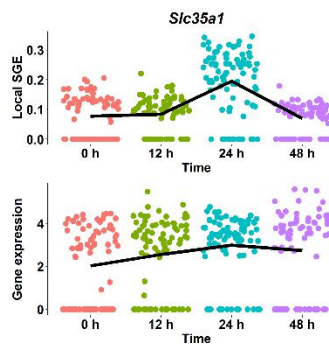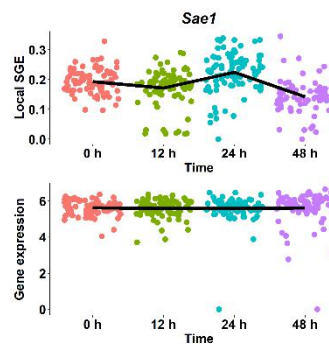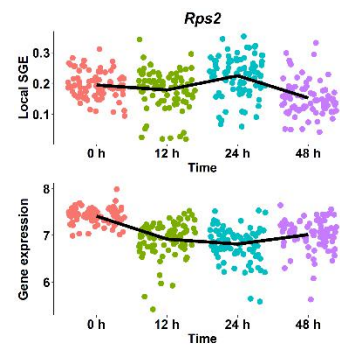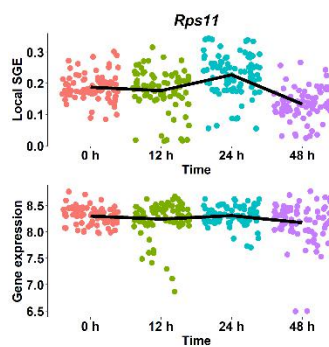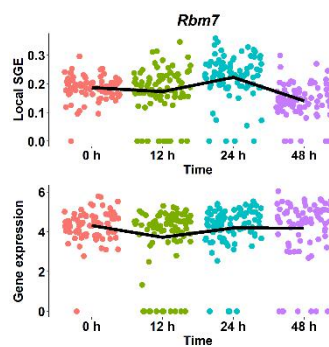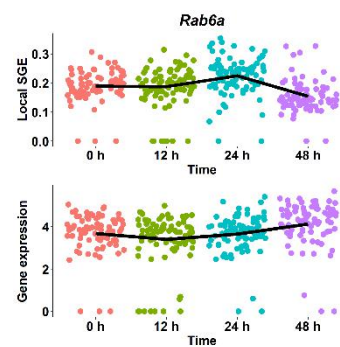

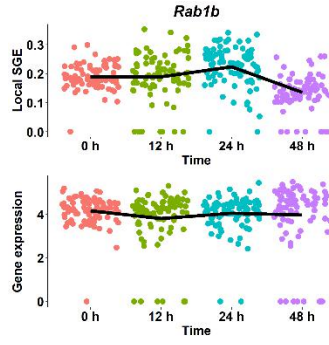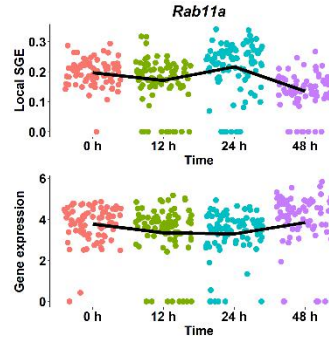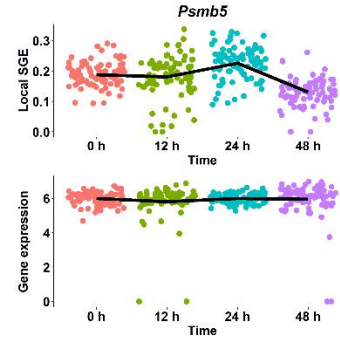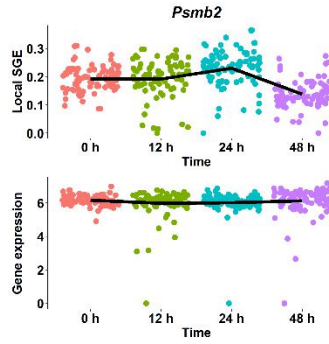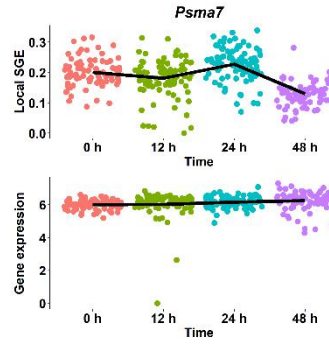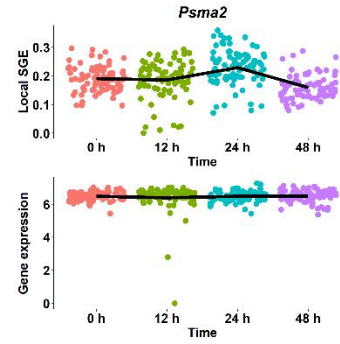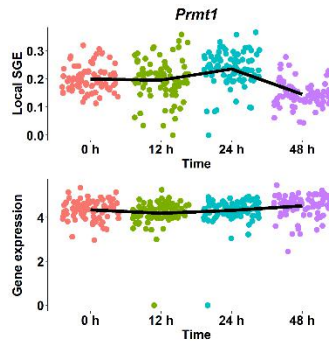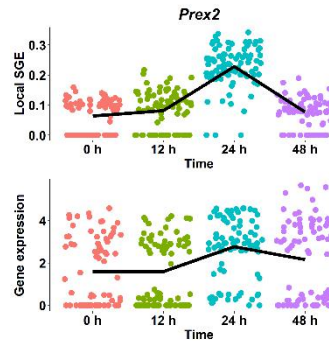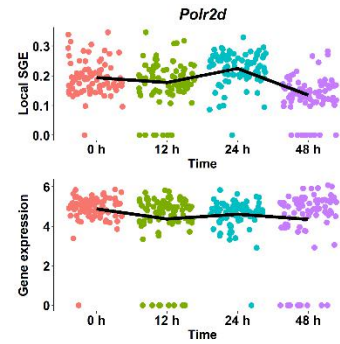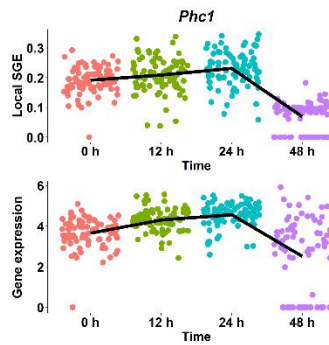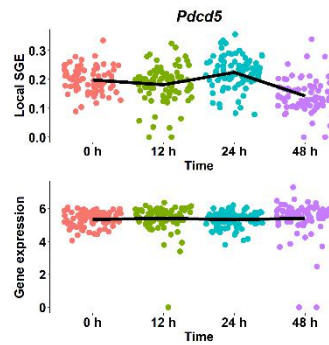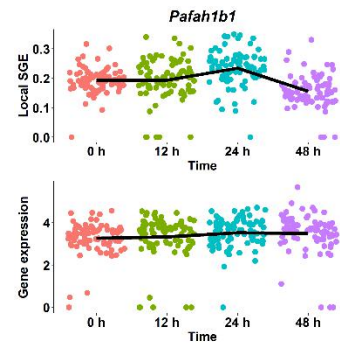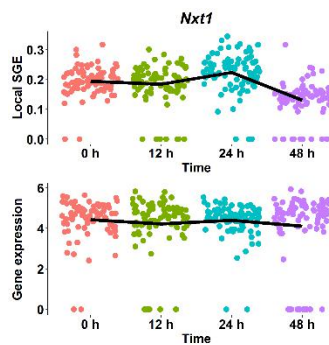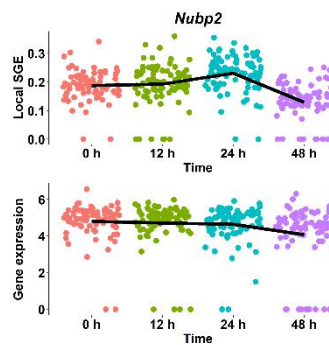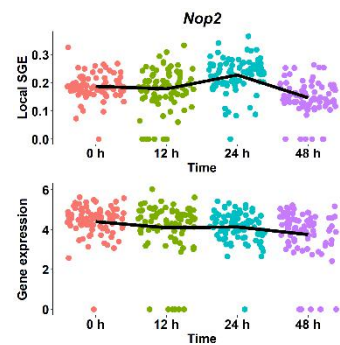

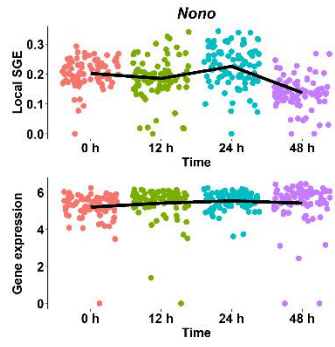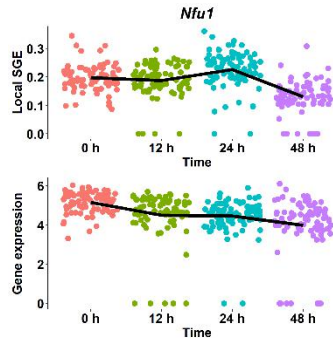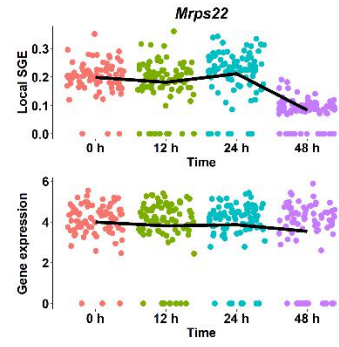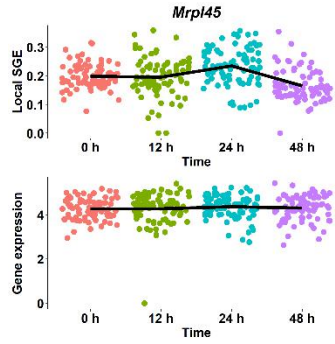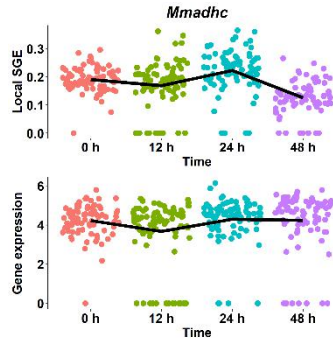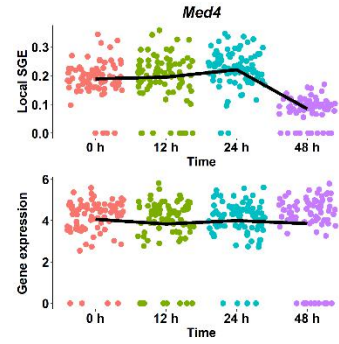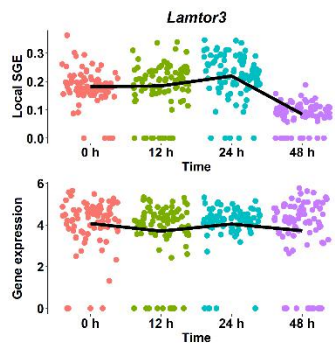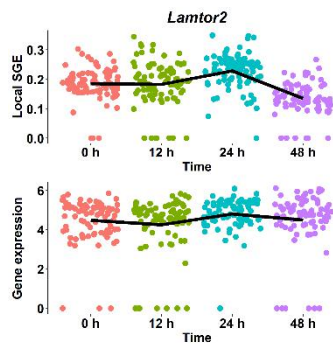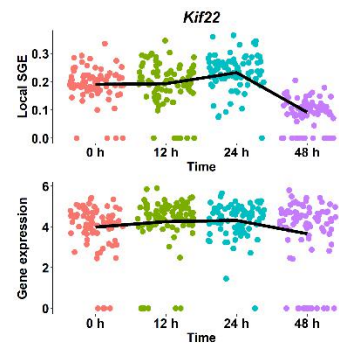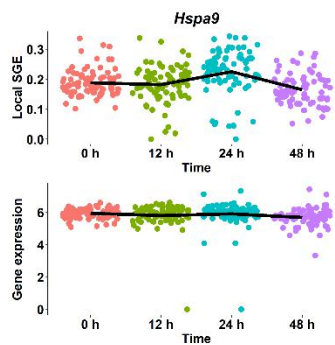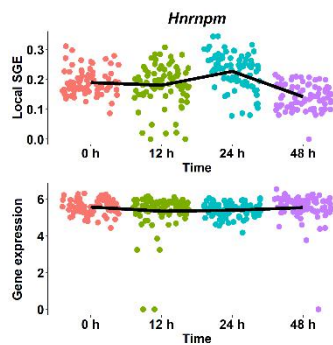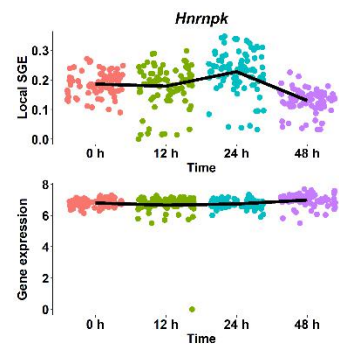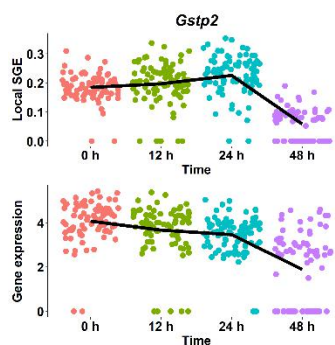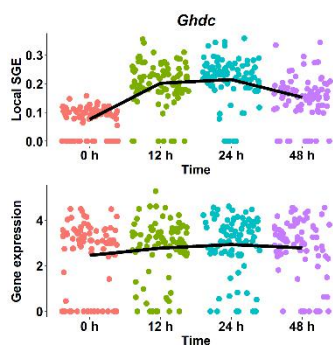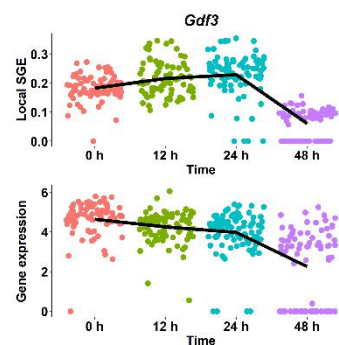

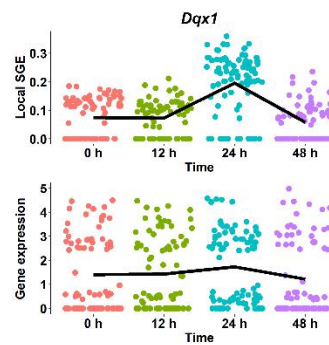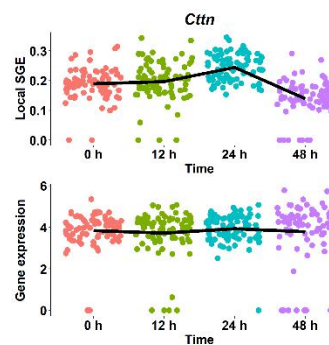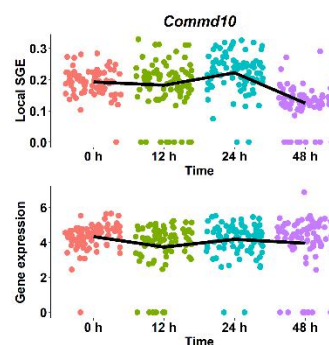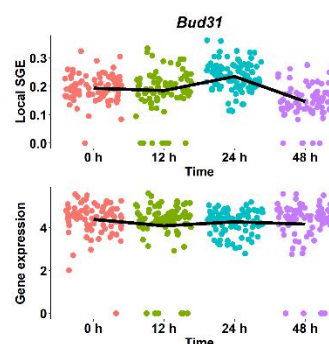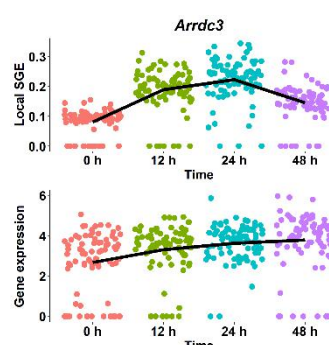

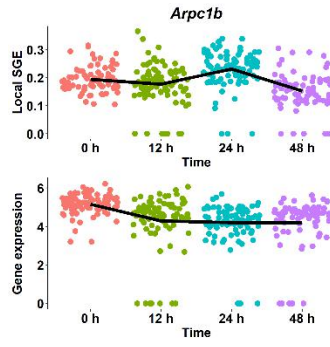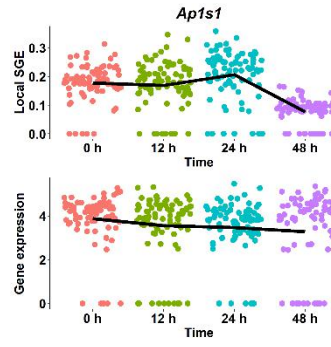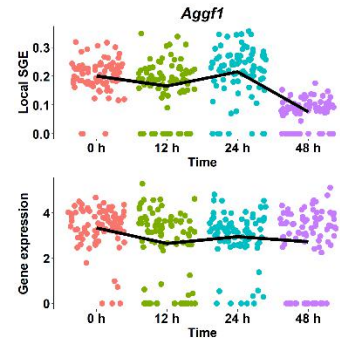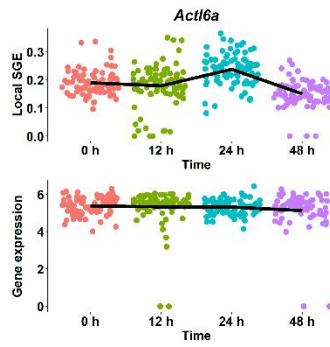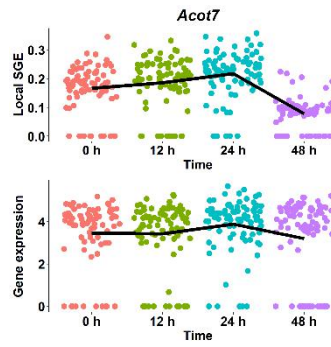

Supplement: Supplementary Figure S12 [file mmc14.pdf]

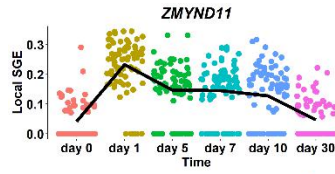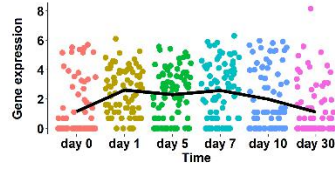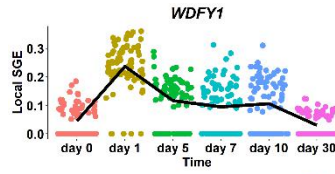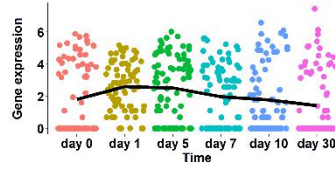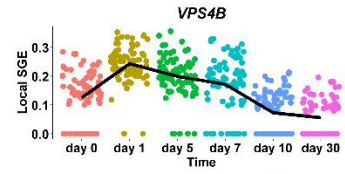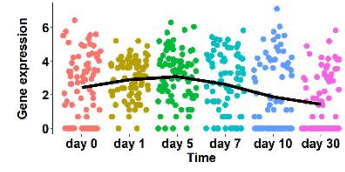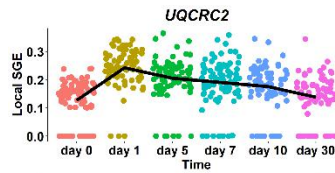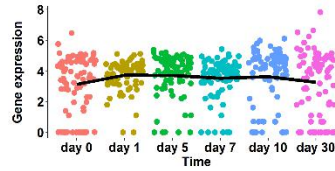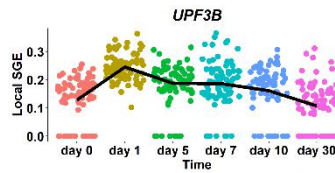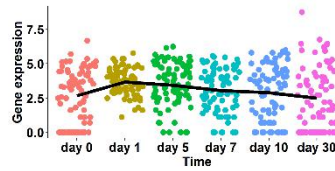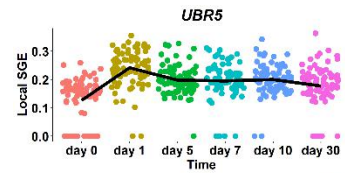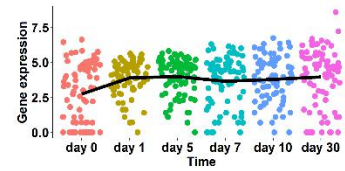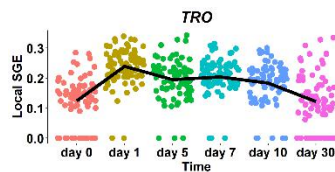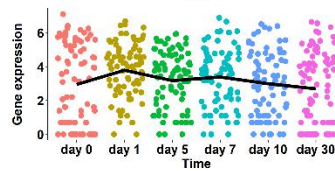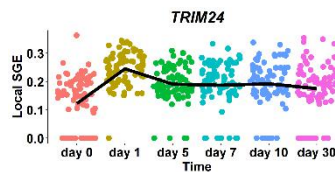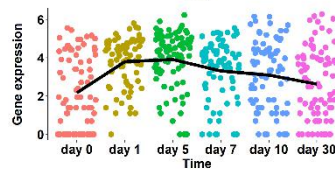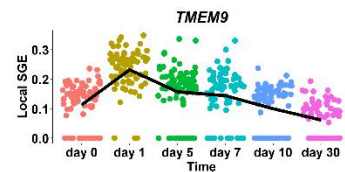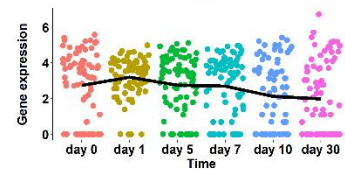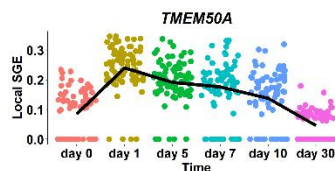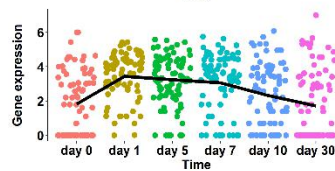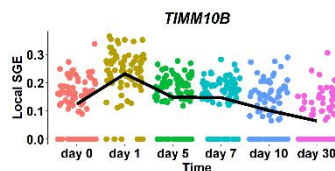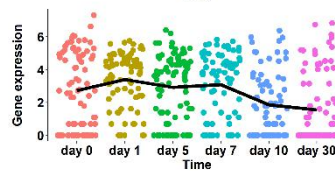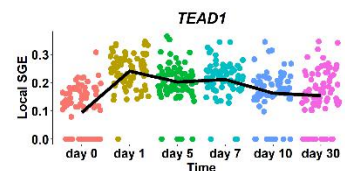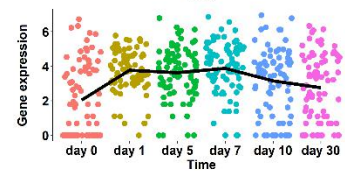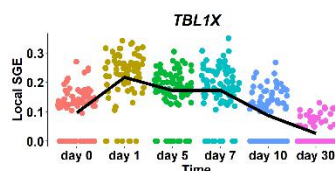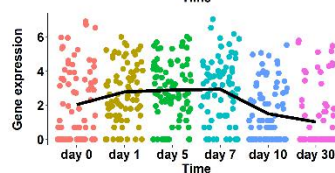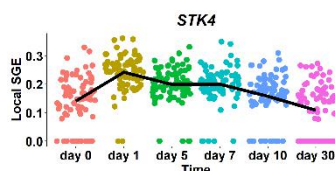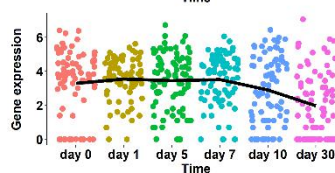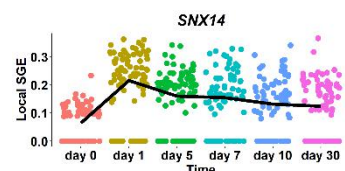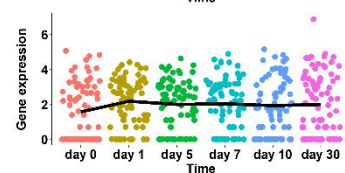

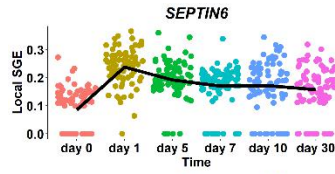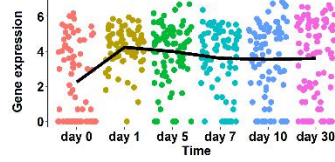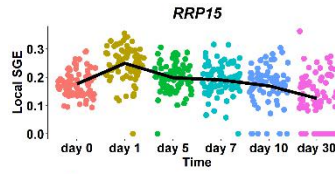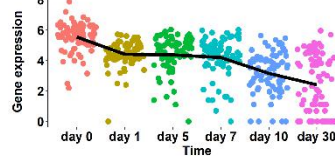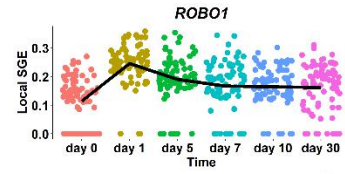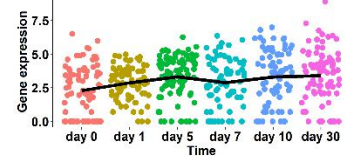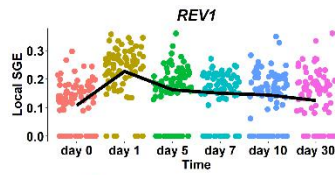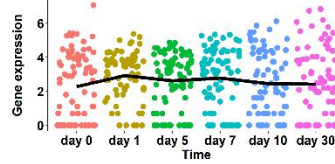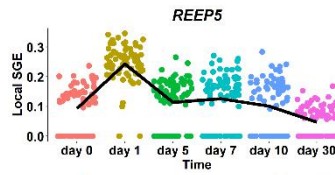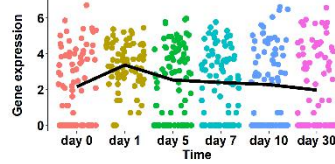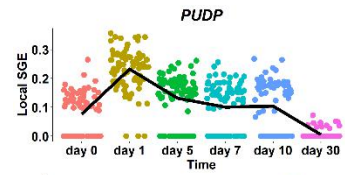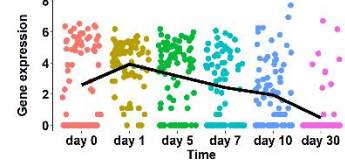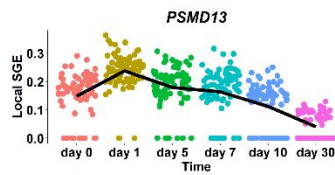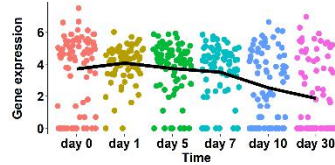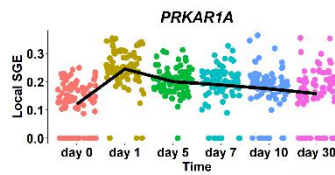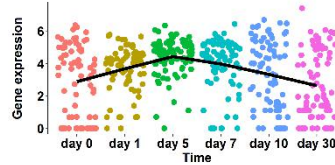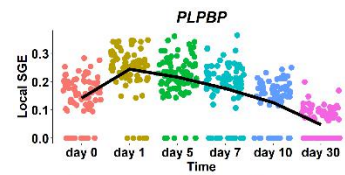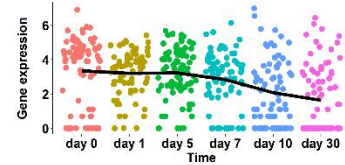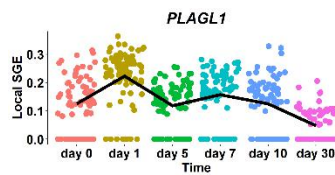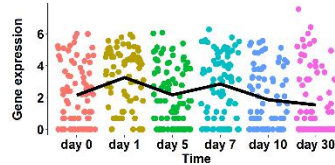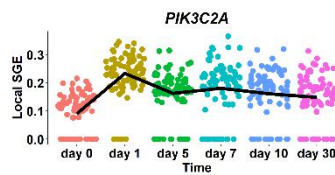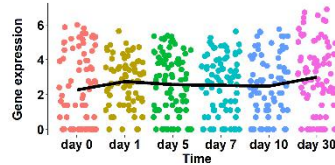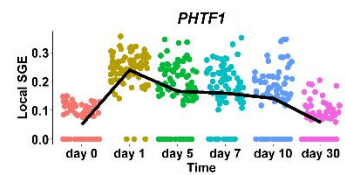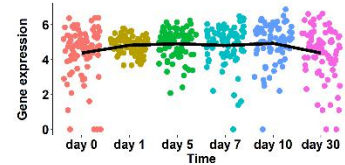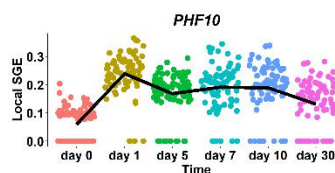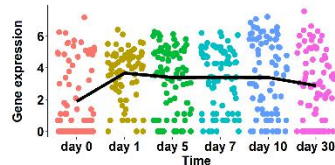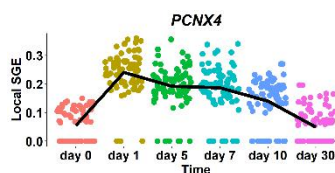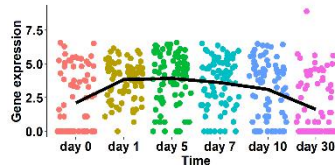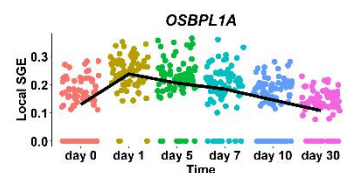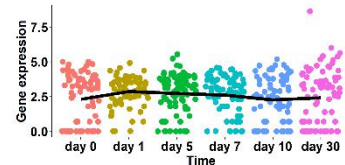

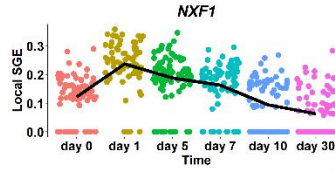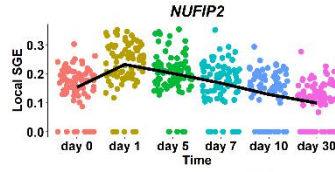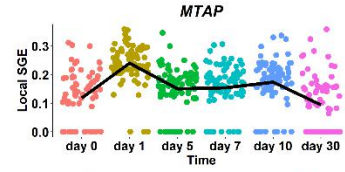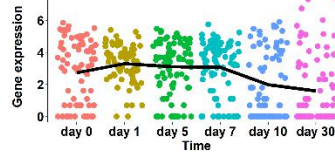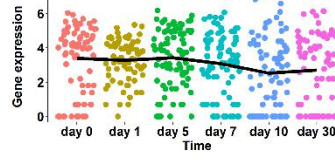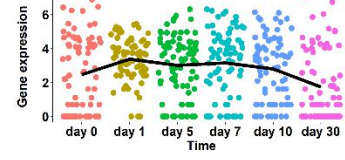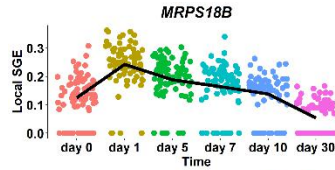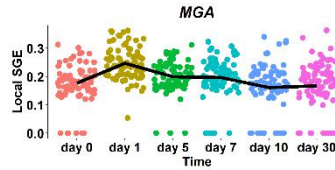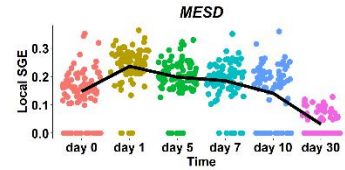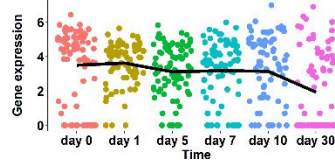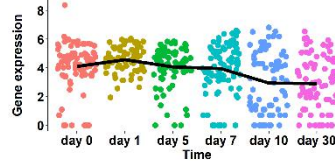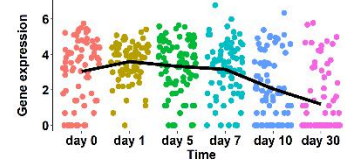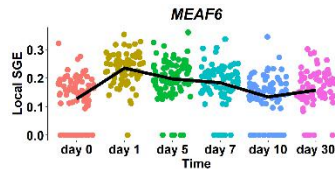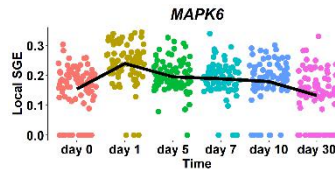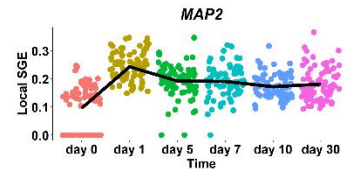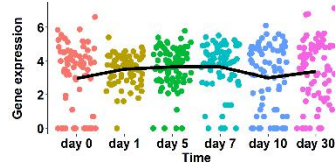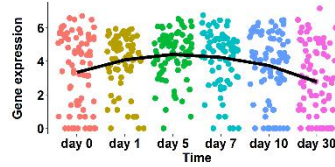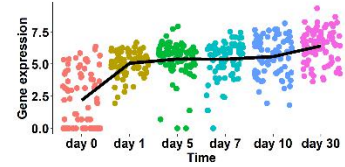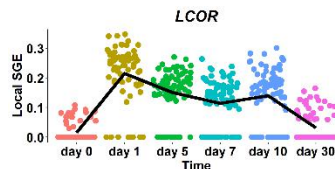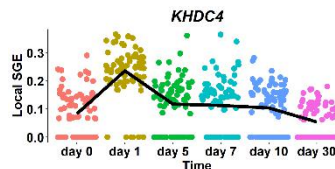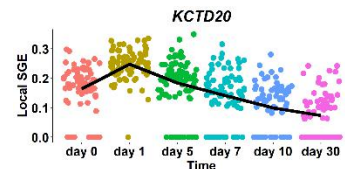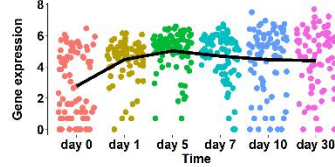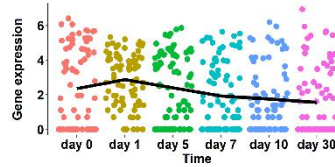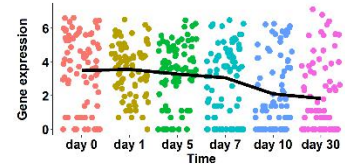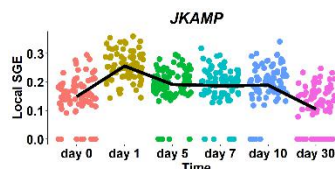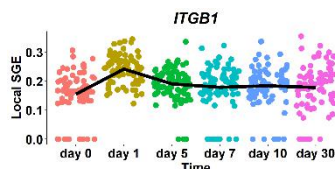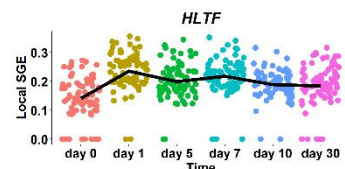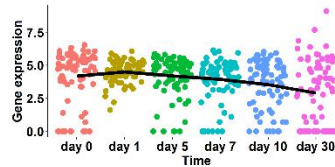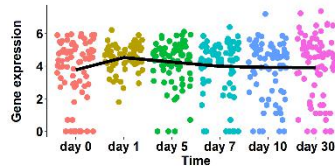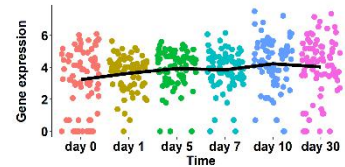

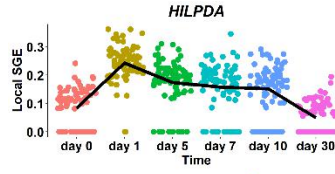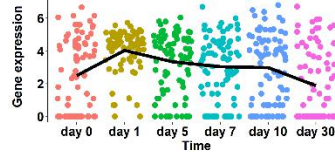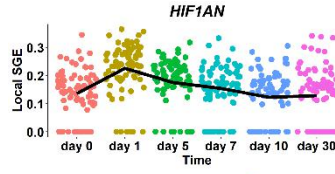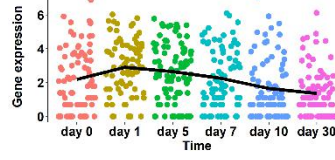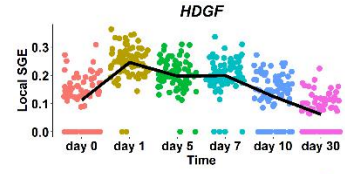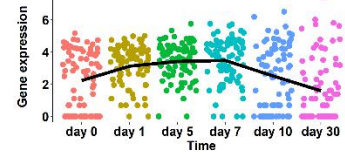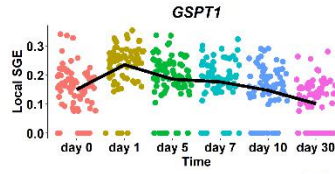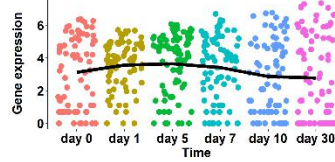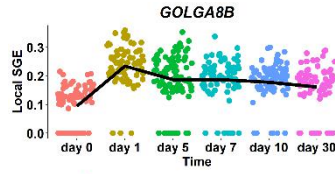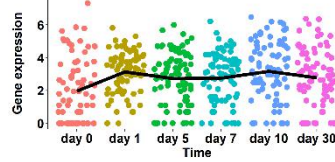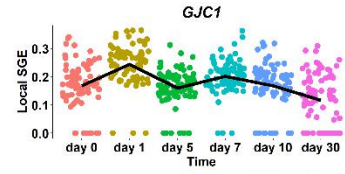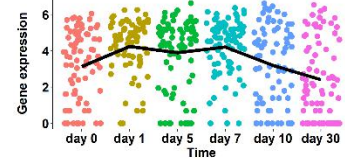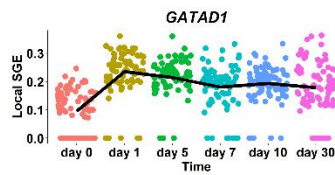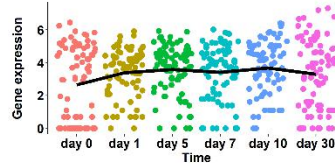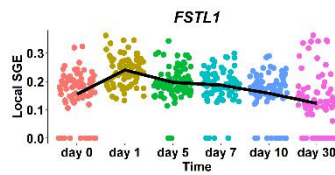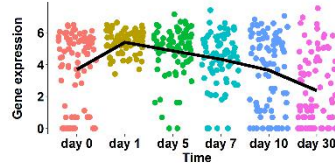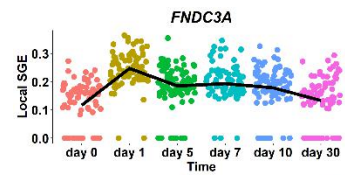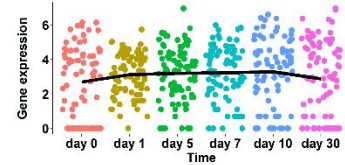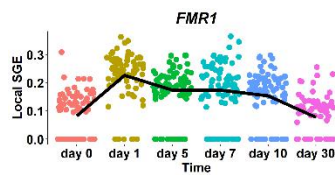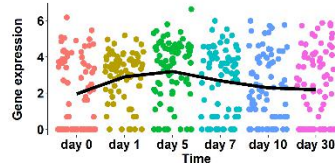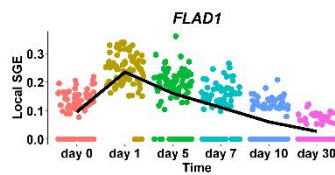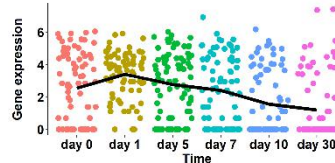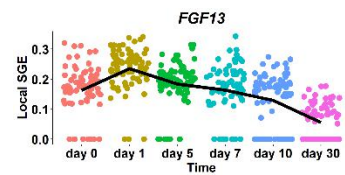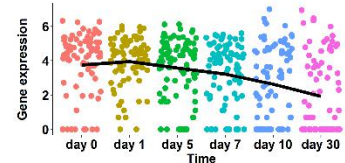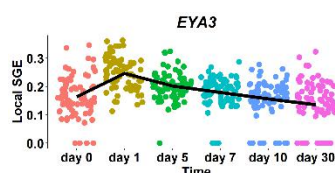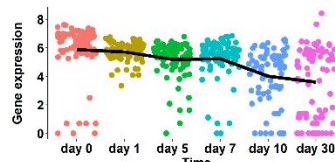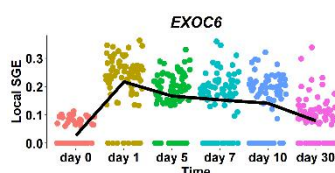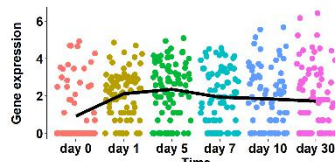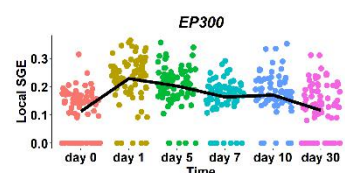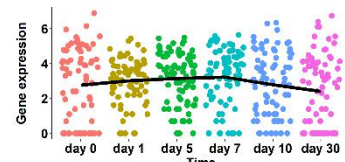

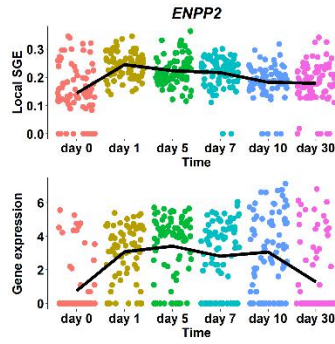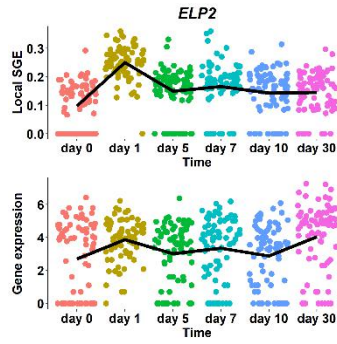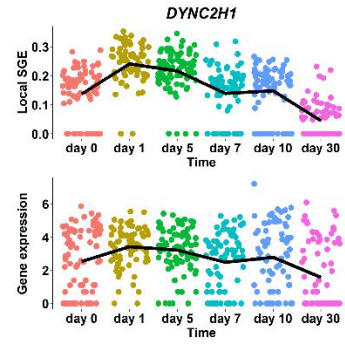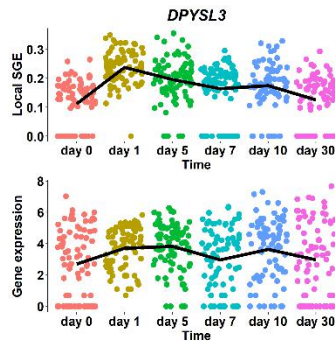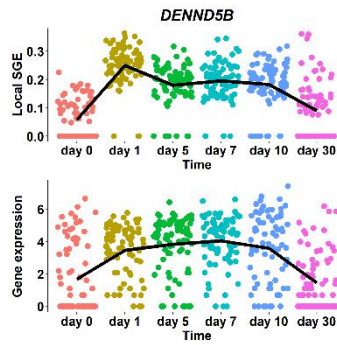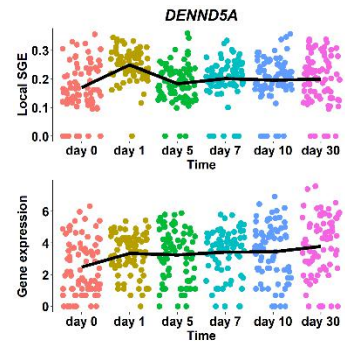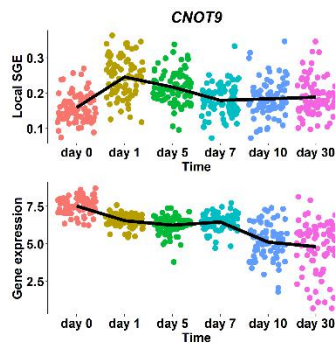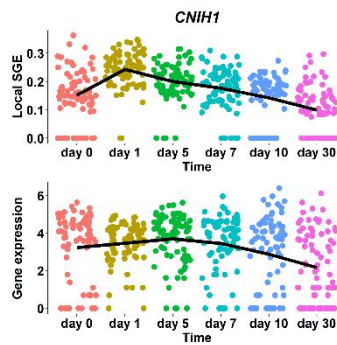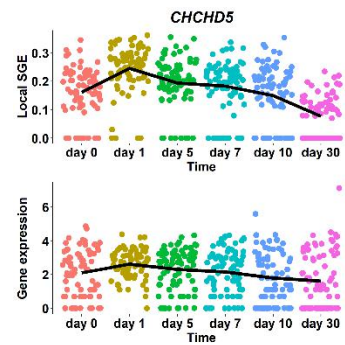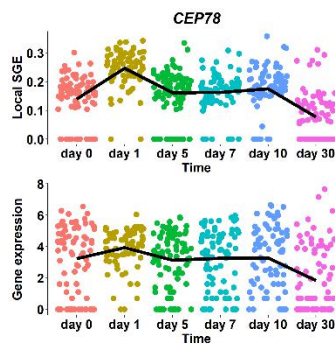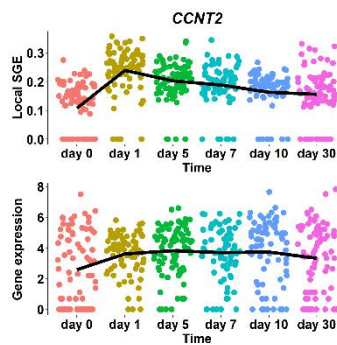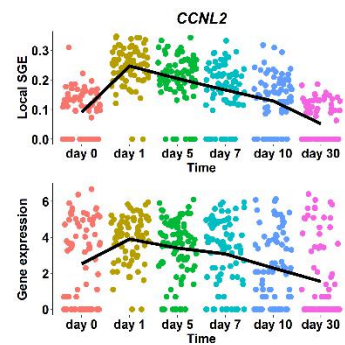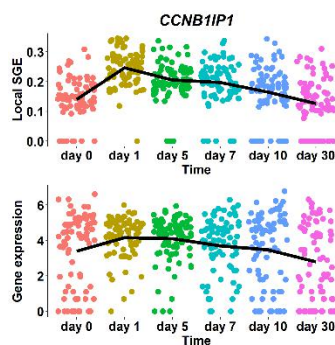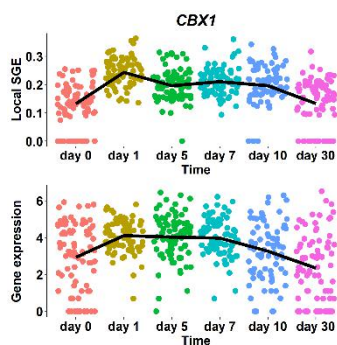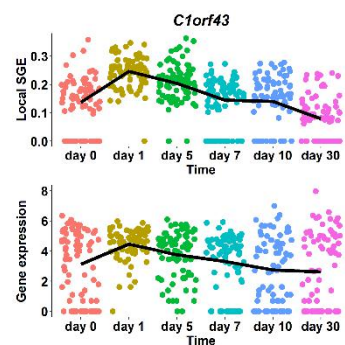

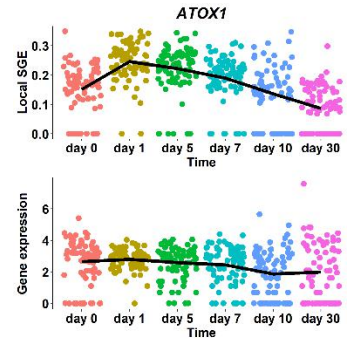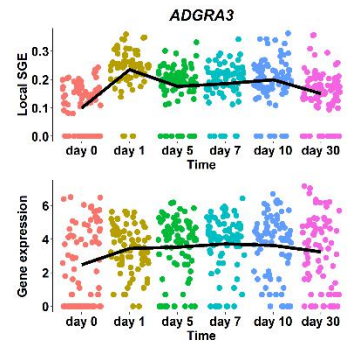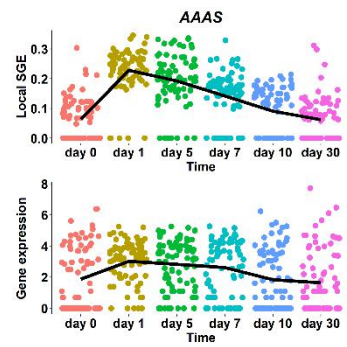

Supplement: Supplementary Figure S13 [file mmc15.pdf]
