# Supplementary material for: scGET: Predicting Cell Fate Transition During Early Embryonic Development by Single-cell Graph Entropy
Source: Genomics Proteomics Bioinformatics. 2021 Dec 24;19(3):461–74. doi: 10.1016/j.gpb.2020.11.008 (PMC8864248; doi:10.1016/j.gpb.2020.11.008)
Supplement: Supplementary Table S14 [file mmc16.pdf]

A The overlap of signaling genes of human datasets

B The overlap of signaling genes of mouse datasets

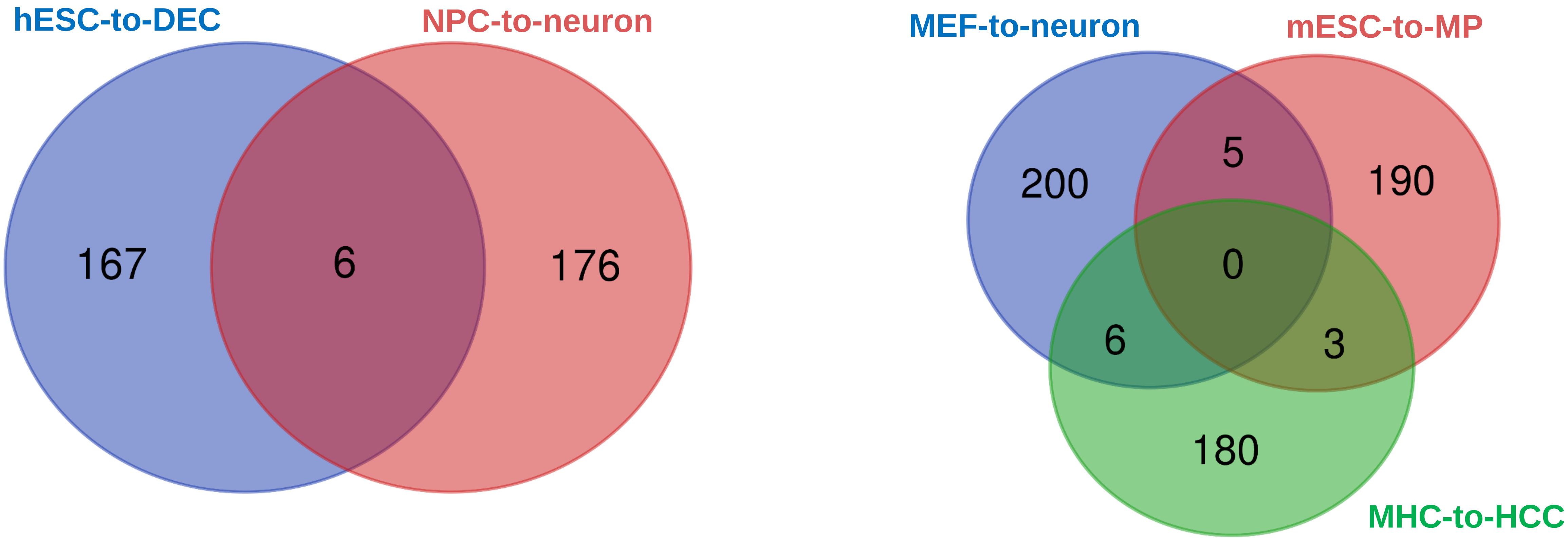

The 6 common signaling genes: *HLTF, LCOR, MGA, RAB3B, KCTD20, PHTF1*

The 14 common signaling genes: *Wtap, Dpp3, 2410015M20Rik, Gtpbp4, Bud31, Pdcd5, Polr2d, Cttn, Azi2, Bag1, Phldb2, H2-D1, Atp6v1b2, Kdelr2*
