# Supplementary material for: scGET: Predicting Cell Fate Transition During Early Embryonic Development by Single-cell Graph Entropy
Source: Genomics Proteomics Bioinformatics. 2021 Dec 24;19(3):461–74. doi: 10.1016/j.gpb.2020.11.008 (PMC8864248; doi:10.1016/j.gpb.2020.11.008)
Supplement: Supplementary Table S2 [file mmc18.docx]

**Table S2** **Signaling dark genes in MEF-to-neuron data**

| Gene | Location | IPA gene family | Relation with embryonic development | PMID |
| --- | --- | --- | --- | --- |
| *Poldip2* | Cytoplasm | Other | Knockout results in perinatal lethality, reduced cellular growth, and increased autophagy of MEF | 24797518 |
| *Hint1* | Nucleus | Enzyme | Deletion enhances the growth of MEF | 12810953 |
| *Eif4g2* | Cytoplasm | Translation regulator | Eifg42 is crucial for cellular differentiation and its function is as an essential determinant of specific gene expression pathways | 16410684 |
| *Egln1* | Cytoplasm | Enzyme | Required for embryonic development | 24990963 |
| *Dad1* | Cytoplasm | Other | *Dad1* deletion induces an apoptosis-associated embryonic death | 10748466 |
| *Becn1* | Cytoplasm | Other | An autophagy gene essential for early embryonic development | 14657337 |
| *Bag1* | Cytoplasm | Other | Essential for differentiation and survival of hematopoietic and neuronal cells | 16116448 |
| *Ppp2r2d* | Nucleus | Other | Expressed in neural precursor and immature neurons of mouse neocortex | 19448628 |

*Note*: Signaling dark genes are dark genes that also belong to the signaling gene set. Dark genes refer to genes with non-differential expression (*P* ≥ 0.05; t-test) but differential SGE value (*P* < 0.05; t-test). Signaling genes are defined as the top 5% genes with the highest local SGE values at the tipping point. IPA, Ingenuity Pathway Analysis (<http://www.ingenuity.com/products/ipa>); MEF, mouse embryonic fibroblast.
