# Supplementary material for: scGET: Predicting Cell Fate Transition During Early Embryonic Development by Single-cell Graph Entropy
Source: Genomics Proteomics Bioinformatics. 2021 Dec 24;19(3):461–74. doi: 10.1016/j.gpb.2020.11.008 (PMC8864248; doi:10.1016/j.gpb.2020.11.008)
Supplement: Supplementary Table S3 [file mmc19.docx]

**Table S3** **Signaling dark genes in MHC-to-HCC data**

| Gene | Location | IPA gene family | Relation with embryonic development | PMID |
| --- | --- | --- | --- | --- |
| *Wtap* | Nucleus | Other | *Wtap* is required for differentiation of endoderm and mesoderm in the mouse embryo | 18224709 |
| *Tcea3* | Nucleus | Transcription regulator | *Tcea3* can regulate the pluripotent differentiation potential of mouse embryonic stem cells via the lefty1-nodal-smad2 pathway | 23169579 |
| *Rab14* | Cytoplasm | Enzyme | *Rab14* is critical for early embryonic development by transporting FGF receptor | 21238925 |
| *Lgr4* | Plasma membrane | Transmembrane receptor | Lgr4 is closely related to the process of embryonic development in Lgr4 knockout mouse model | 15192078 |
| *Cdk6* | Nucleus | Kinase | *Cdk6* has sub-type speciﬁc and cell cycle regulation-independent functions utilized during embryonic development and differentiation of stem cells | 19040567 |
| *Casp3* | Cytoplasm | Peptidase | *Casp3* promotes the differentiation of murine embryonic stem cell by cleaving the pluripotency factor Nanog | 18522852 |
| *Arpc3* | Cytoplasm | Other | *Arpc3* is essential for mouse preimplantation embryo development | 22951093 |
| *Uap1* | Nucleus | Enzyme | Defective *Fancd2* regulated by *Uap1* leads to the increase in chromosomal instability in mESCs and mouse embryonic lethality | 24001775 |

*Note*: Signaling dark genes are dark genes that also belong to the signaling gene set. Dark genes refer to genes with non-differential expression (*P* ≥ 0.05; t-test) but differential SGE value (*P* < 0.05; t-test). Signaling genes are defined as the top 5% genes with the highest local SGE values at the tipping point. IPA, Ingenuity Pathway Analysis (<http://www.ingenuity.com/products/ipa>); MHC, mouse hepatoblasts cell; HCC, hepatocytes and cholangiocytes cell; FGF, fibroblast growth factor; mESC, mouse embryonic stem cell.
