# Supplementary material for: scGET: Predicting Cell Fate Transition During Early Embryonic Development by Single-cell Graph Entropy
Source: Genomics Proteomics Bioinformatics. 2021 Dec 24;19(3):461–74. doi: 10.1016/j.gpb.2020.11.008 (PMC8864248; doi:10.1016/j.gpb.2020.11.008)
Supplement: Supplementary Table S4 [file mmc20.docx]

**Table S4** **Signaling dark genes in hESC-to-DEC data**

| Gene | Location | IPA gene family | Relation with embryonic development | PMID |
| --- | --- | --- | --- | --- |
| *TMOD1* | Cytoplasm | Enzyme | The absence of *TMOD1* in differentiating embryonic stem cells leads to delayed myofibril assembly | 21078668 |
| *LAMC2* | Extracellular space | Other | *LAMC2* is an epithelial basement membrane protein implicated in cell proliferation and differentiation | 27333824 |
| *ZEB2* | Nucleus | Translation regulator | *ZEB2* expression is essential for embryonic hematopoietic stem and progenitor cell differentiation in the fetal liver | 28126955 |
| *ZNF521* | Nucleus | Transcription regulator | *ZNF521* can efficiently drive embryonic stem cells to neural progenitors | 21326203 |
| *IGF1* | Extracellular space | Growth  factor | *IGF1* supports the derivation and maintenance of human pluripotent stem cells, together with Activin | 32034154 |
| *COL4A1* | Extracellular space | Other | The balance of *COL4A1* and other ECM gene expression is essential to support human embryonic stem cell maintenance | 23658023 |
| *SCML2* | Nucleus | Transcription regulator | *SCML2* plays an essential role in the modulation of self- renewal and differentiation of embryonic stem cells | 24358021 |
| *FGF2* | Extracellular space | Growth factor | *FGF2* switches the outcome of BMP4-induced human embryonic stem cell differentiation | 21362572 |

*Note*: Signaling dark genes are dark genes that also belong to the signaling gene set. Dark genes refer to genes with non-differential expression (*P* ≥ 0.05; t-test) but differential SGE value (*P* < 0.05; t-test). Signaling genes are defined as the top 5% genes with the highest local SGE values at the tipping point. IPA, Ingenuity Pathway Analysis (<http://www.ingenuity.com/products/ipa>); hESC, human embryonic stem cell; DEC, definitive endoderm cell; ECM, extracellular matrix.
